# Encounter Complex of Adenine with Carboplatin and Oxaliplatin Anticancer Drugs Elucidated by IRMPD Spectroscopy and Theoretical Study

**DOI:** 10.1021/acs.inorgchem.4c04731

**Published:** 2025-03-06

**Authors:** Barbara Chiavarino, Lucretia Rotari, Maria Elisa Crestoni, Davide Corinti, Debora Scuderi, Jean-Yves Salpin

**Affiliations:** † Dipartimento di Chimica e Tecnologie del Farmaco, Università di Roma “La Sapienza”, P.le A. Moro 5, I-00185 Roma, ITALY; ‡ Université Paris-Saclay, CNRS, Institut de Chimie-Physique, 91405 Orsay, France; § Université Paris-Saclay, Univ Evry, CY Cergy Paris Université, CNRS, LAMBE, 91025 Evry-Courcouronnes, France

## Abstract

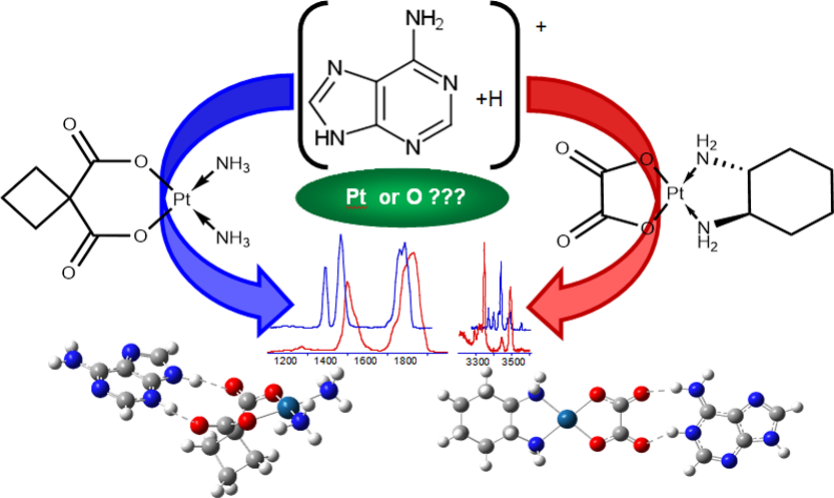

Ionic complexes containing the nucleobase adenine and either carboplatin (CarboPt) or oxaliplatin (OxaliPt) were generated in solution and subsequently studied in the gas phase by combining tandem mass spectrometry, infrared multiple photon dissociation (IRMPD) spectroscopy, and density functional theory (DFT) calculations. The protonated complexes of the general formula [Pt drug+H+adenine]^+^ were first analyzed by collision-induced dissociation (CID). Their CID mass spectra show only one fragment, corresponding to the loss of neutral adenine. The structure of these complexes was elucidated by comparing their IRMPD spectra recorded in the fingerprint and H-X stretching ranges with DFT-calculated IR spectra. Unexpectedly, the IRMPD spectra of both complexes were not consistent with the calculated vibrational spectra of structures characterized by direct platinum–adenine coordination. All spectroscopic evidence suggest that each sampled [Pt drug+H+adenine]^+^ ion population comprises multiple proton-bound complexes stabilized by hydrogen bonds between the drug carboxylate groups and protonated adenine. Interestingly, while calculations support an external binding scheme in protonated adenine-oxaliplatin complexes, in the case of carboplatin, a direct monodentate interaction of Pt with N1, N3, or N7 positions of adenine turns out to be energetically favored. This study adds further evidence of the intrinsic lower affinity of platinum for adenine relative to guanine.

## Abstract 2

The structure of gaseous [CarboPt+H+adenine]^+^ and [OxaliPt+H+adenine]^+^ complexes were investigated by combining collision-induced dissociation (CID) experiments, infrared multiple photon dissociation spectroscopy (IRMPD), and density functional theory (DFT) calculations. These complexes strongly differ from those obtained with guanine as there is no direct coordination of the platinum atom to the nucleobase, but instead a mixture of several proton-bound adducts. This finding is further evidence of the intrinsic lower affinity of platinum for adenine.

## 1 Introduction

**Scheme 1:**
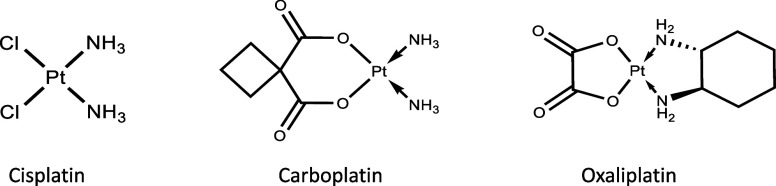
Chemical Structures of Cisplatin, Carboplatin, and Oxaliplatin

Carboplatin (cis-diammine-[1,1-cyclobutanedicarboxylato]platinum(II)), (CarboPt), and oxaliplatin [(1R,2R-diamminocyclohexane)oxalatoplatinum(II)] (OxaliPt) are the only second- and third-generation platinum compounds used worldwide in cancer therapy. They were designed in the early 1970s, following the significant therapeutic success of cisplatin (cis-diaminedichloroplatinum(II)) in the treatment of many cancers to reduce the serious side effects and acquired tumor cell resistance of the first-generation platinum drug.^1^ Both drugs share the same final mechanism of action as cisplatin, exerting their cytotoxic activity through the coordination, in two consecutive steps, to the N7 atoms of two adjacent purines of the same DNA strand.^2^ The formation of intrastrand cross-links, predominantly, 1,2-d(GpG) or 1,2-d(ApG), which together represent 80–90% of the bound Pt,^3^ causes a strong distortion of the DNA helix, inhibits its replication, and finally leads to cell death by apoptosis.^4^ Like cisplatin, they have a square planar geometry around the platinum atom (Scheme 1), wherein the two chlorido ligands of cisplatin were replaced by a bulkier chelating ligand, the cyclobutanedicarboxyl group in carboplatin and the oxalato ligand in oxaliplatin.

This ligand substitution was designed by researchers to slow down the first step of cisplatin activation, namely, the nucleophilic substitution of chloride with a water molecule that yields the reactive intermediate, cis-[Pt(NH_3_)_2_(H_2_O)Cl]^+^. This monoaqua complex forms only in the cytoplasm, where it is favored by a lower concentration of chloride anions. Further ligand exchange of water/purine leads to the formation of the first bond between the platinum drug and DNA and also to a variety of biological targets, ultimately responsible for toxic side effects and resistance.

Consequently, the presence of a bulkier and more strongly bound ligand than chloride is expected to modify the initial interactions between DNA and second- and third-generation platinum drugs. From a pharmacological point of view, carboplatin is particularly effective against testicular and ovarian tumors, showing the same spectrum of activity as cisplatin but with lower neurotoxicity and nephrotoxicity.^5^ However, carboplatin shows the same drug resistance as cisplatin. In contrast, oxaliplatin has demonstrated *in vitro* and *in vivo* efficacy against many cisplatin- and carboplatin-resistant tumor cell lines and is very effective in the treatment of colorectal cancer.^6^ The different spectrum of activity and the lower drug resistance of OxaliPt are attributed to the presence of the ligand 1,2-diaminocyclohexane (DACH), which allows the formation of OxaliPt-DNA adducts more cytotoxic and efficient in blocking DNA replication relative to cisplatin.^7^

Despite numerous studies on the antineoplastic activity of CarboPt and OxaliPt, the first phase of activation of these drugs is still unclear, and the details of how they exert their cytotoxic effects are not completely understood. In our previous studies on the interactions occurring between cisplatin and nucleobases^8^ or deoxynucleotides,^9^ the complexes containing adenine have offered us some unexpected results. The combination of these two considerations prompted us to characterize the structure of the naked ionic complexes between oxaliplatin and carboplatin and their primary targets, namely, the guanine and adenine residues. After a first study describing their reactivity toward guanine,^10^ the present work reports the detailed structural description of the complexes generated in the presence of adenine. To elucidate the binding motif inside the complexes of interest, we combined the results obtained from tandem mass spectrometry experiments and infrared multiple photon dissociation (IRMPD) action spectroscopy with density functional theory (DFT) calculations. IRMPD is recognized to be an effective technique for the structural characterization of biomolecular ions,^11^ notably arising from the interaction of DNA building blocks with metal ions.^12^ Our results again point to a net difference in the interaction process of the two nucleobases toward the two platinum drugs.

## 2 Experimental Section

### 2.1 Materials

All reagents and solvents used in this work, namely, 2′-deoxyadenosine-5′-monophosphate (5′-dAMP), oxaliplatin, carboplatin, methanol, and water, were research-grade products from commercial sources (Merck s.r.l. Milan, Italy) and were used as received. Stock water solutions of 5′-dAMP and the two Pt-based drugs were prepared at a 10^–3^ M concentration. To overcome the poor solubility of adenine in any solvent, 5′-dAMP was utilized as a source of nucleobase to obtain the complexes of interest. In water solution, hydrolysis promotes the glycosidic bond cleavage of 5′-dAMP, yielding adenine as previously reported for adenosine.^13^ Hence, the stock solutions of 5′-dAMP and the selected Pt drug were mixed together in the molar ratio of 1:1 in a water/methanol solution (final concentration of 10^–5^ M) and then allowed to react for 24 h at room temperature.

### 2.2 CID Experiments

The CID experiments were carried out using a linear ion trap mass spectrometer (LTQ XL from Thermo Scientific) coupled to an electrospray ionization (ESI) source. The solutions were infused to the ESI source using a syringe pump at a flow rate of 10 μL min^–1^. Parameters for CID experiments were set as follows: temperature, 250 °C; spray voltage, 5000 V; sheath gas flow rate, 10 (arbitrary units); aux gas flow rate, 2 (arbitrary units); and tube lens voltage, 50 V.

### 2.3 IRMPD Experiments

Two spectral ranges were explored by IRMPD experiments. The fingerprint range (800–2000 cm^–1^) was investigated with the beamline of the free electron laser (FEL) of the Centre Laser Infrarouge d’Orsay (CLIO).^14^ Sample solutions were directly infused in a hybrid FT-ICR tandem mass spectrometer (APEX-Qe Bruker), equipped with a 7.0 T actively shielded magnet and a quadrupole-hexapole interface for mass-filtering and ion accumulation and coupled to the beamline of the CLIO FEL. Details of this experimental setup can be found in a previous publication.^15^ Positively charged ions generated by ESI were mass-selected in the quadrupole, accumulated, and collisionally cooled with argon buffer gas for 300 ms in the hexapole and finally transferred into the ICR cell prior to irradiation with the IR FEL light at a repetition rate of 25 Hz for 180–500 ms. Each macropulse has a typical energy of 40 mJ. For this study, the electron energy of the FEL was adjusted at 44.4 MeV. Saturation effects of the most intense absorptions were avoided by recording IRMPD spectra with the use of one or two attenuators to reduce saturation effects.

The X-H (X = C, N, O) stretching range (2900–3700 cm^–1^) was probed by recording IRMPD spectra using an optical parametric oscillator/amplifier (OPO/OPA, LaserVision) system coupled to a Paul ion trap mass spectrometer (Esquire 6000+, Bruker Daltonics), as already reported.^16^ The OPO/OPA system is pumped by the 1064 nm fundamental of a nonseeded Nd:YAG laser (Continuum Surelite II) operating at 9 Hz repetition rates and produces an output energy of ca. 19 mJ/pulse with 3–4 cm^–1^ bandwidth, in the spectral range of investigation. In the Paul trap, mass-selected ions were accumulated for 5–50 ms and then irradiated with an IR light for 0.5–4 s.

In a typical IRMPD experiment, the absorption of multiple resonant IR photons leads to an increase in the internal energy of the mass-selected ion, causing its unimolecular dissociation and the appearance of product ions in the mass spectrum. The IRMPD spectrum is obtained by plotting the photofragmentation yield *R* (*R* = −ln[*I*_precursor_/(*I*_precursor_ + Σ*I*_product_)], where *I*_precursor_ and *I*_product_ are the integrated intensities of the mass peaks of the precursor and of the product ions, respectively) as a function of the wavenumber of the IR radiation.^14b,17^

### 2.4 Theoretical Study

To optimize the structures of the various complexes presently studied, we used the Gaussian-16 set of programs^18^ and performed DFT calculations by using the hybrid B3LYP functional.^19^ This particular functional has been shown to be very efficient to compute both position and relative intensities of the IR band^20^ and is notably suitable for both the structural and IR characterization of Pt complexes, provided the use of appropriate scaling factors.^21^ We carried out geometry optimization without any symmetry constraint by describing C, H, N, and O atoms with the 6-311G** basis set. To describe the metallic center, we used the Los Alamos effective core potential (ECP) in combination with the LACV3P** basis set.^22^ For the sake of simplicity, this combined basis set is termed 6-311G** in the text. Harmonic vibrational frequencies were also computed at this level to estimate the zero-point vibrational energy corrections and to characterize the stationary points as local minima or saddle points. The successful combination of B3LYP/Los Alamos ECP+basis sets for the investigation of electronic properties, vibrational properties, and reaction pathways of platinum-containing compounds, including cisplatin, carboplatin, and oxaliplatin, has been well documented by different groups,^23^ as well as by ourselves.^8−10,24^ Presently, we have calculated the infrared absorption spectra of the various structures within the harmonic approximation by using a scaling factor of 0.974 in the fingerprint region and 0.957 in the X-H stretching region, in agreement with previous studies.^8−10^ So far, we systematically obtained, for the three platinum drugs, an overall good agreement between the computed experimental IRMPD spectra. For comparison purposes with the experimental spectra, the calculated spectra have been convoluted with a Lorentzian profile of 5 cm^–1^ fwhm (full width at half-maximum) in the 2900–3700 cm^–1^ frequency range, while a fwhm of 15 cm^–1^ was used for the 800–2000 cm^–1^ range. Throughout this paper, total energies are expressed in Hartrees and relative energies in kJ mol^–1^.

## 3 Results and Discussion

### 3.1 Collision-Induced Dissociation Experiments

When analyzing the solution containing both 5′-dAMP and OxaliPt by ESI-MS, a cluster of major peaks at *m*/*z* 532/534 appears in the mass spectrum whose isotopic pattern conforms to the theoretical one for the [OxaliPt+H+A]^+^ complex, as reported in Figure S1. The accurate ion mass spectrum recorded with the hybrid FT-ICR tandem mass spectrometer (Figure S1b) reveals the exact mass of the monoisotopic peak of [OxaliPt+H+A]^+^ at *m*/*z* 532.12070, confirming the expected elemental composition, [C_13_H_20_N_7_O_4_Pt]^+^ (experimental error of 1.7 ppm). Similarly, the ESI mass spectrum of the 5′-dAMP and CarboPt solution presents a cluster of major peaks at *m*/*z* 506/508, perfectly consistent with the formation of a [CarboPt+H+A]^+^ complex (its high-resolution mass spectrum reported in Figure S2b shows the monoisotopic peak at *m*/*z* 506.10503 in perfect agreement with the simulated elemental composition for [C_11_H_18_N_7_O_4_Pt]^+^). These complexes might be considered the counterparts of those containing guanine (G), previously observed when 5′-dGMP (2′-deoxyguanosine-5′-monophosphate) was used instead of 5′-dAMP.^10^ However, both [OxaliPt+H+A]^+^ and [CarboPt+H+A]^+^ complexes have shown a different dissociation behavior by the CID assay in comparison to that observed with their guanine analogues. Figures S3a and S4a display the CID spectra recorded for both complexes in a linear ion trap mass spectrometer at a 10 eV collision energy (laboratory frame). These CID spectra show only one product ion, namely, the protonated platinum-based drug, centered at *m*/*z* 397 [OxaliPt+H]^+^ and *m*/*z* 371 [CarboPt+H]^+^, respectively, by loss of neutral adenine. The release of the neutral nucleobase, i.e., guanine, was also observed in the CID experiments of [OxaliPt+H+G]^+^ and [CarboPt+H+G]^+^ complexes, although to a much lesser extent.^10^ In fact, the fragmentation paths of guanine complexes mainly involved the platinum-drug moiety, in particular through the elimination of CO_2_ in [OxaliPt+H+G]^+^ and of NH_3_ in [CarboPt+H+G]^+^, as depicted in Figures S3b and S4b, which report the CID spectra of the corresponding guanine adducts recorded at 12 eV collision energy (laboratory frame). Such a substantial difference in the fragmentation pattern may suggest a different binding mode of the two platinum-based drugs with either adenine or guanine. The analogous cisplatin complexes, i.e., *cis-*[Pt(NH_3_)_2_Cl+A]^+^ and *cis-*[Pt(NH_3_)_2_Cl+G]^+^, where both nucleobases were found to bind directly to the platinum atom, showed comparable fragmentation schemes, although the presence of protonated adenine (*m*/*z* 136) indicated a greater tendency of the Pt–A bond to break than the Pt–G bond.^8a^ Herein, the presence of bulkier ligands may result in a further decrease of the affinity for platinum of the adenine ligand, which could explain the different CID fragmentation of [OxaliPt+H+A]^+^ and [CarboPt+H+A]^+^.

To verify which hypothesis (either a different coordination scheme or a lower affinity for adenine by the two platinum-based drugs) better explains the CID results, IRMPD experiments were carried out on [OxaliPt+H+A]^+^ and [CarboPt+H+A]^+^ as isolated complexes.

### 3.2 IRMPD Spectroscopy of the [OxaliPt+H+A]^+^ Complex

To characterize the structure of the adducts [OxaliPt+H+A]^+^ and [CarboPt+H+A]^+^, a comparison between experimental IRMPD spectra and calculated IR spectra of the optimized geometries of potential candidates is needed.

**Figure 1:**
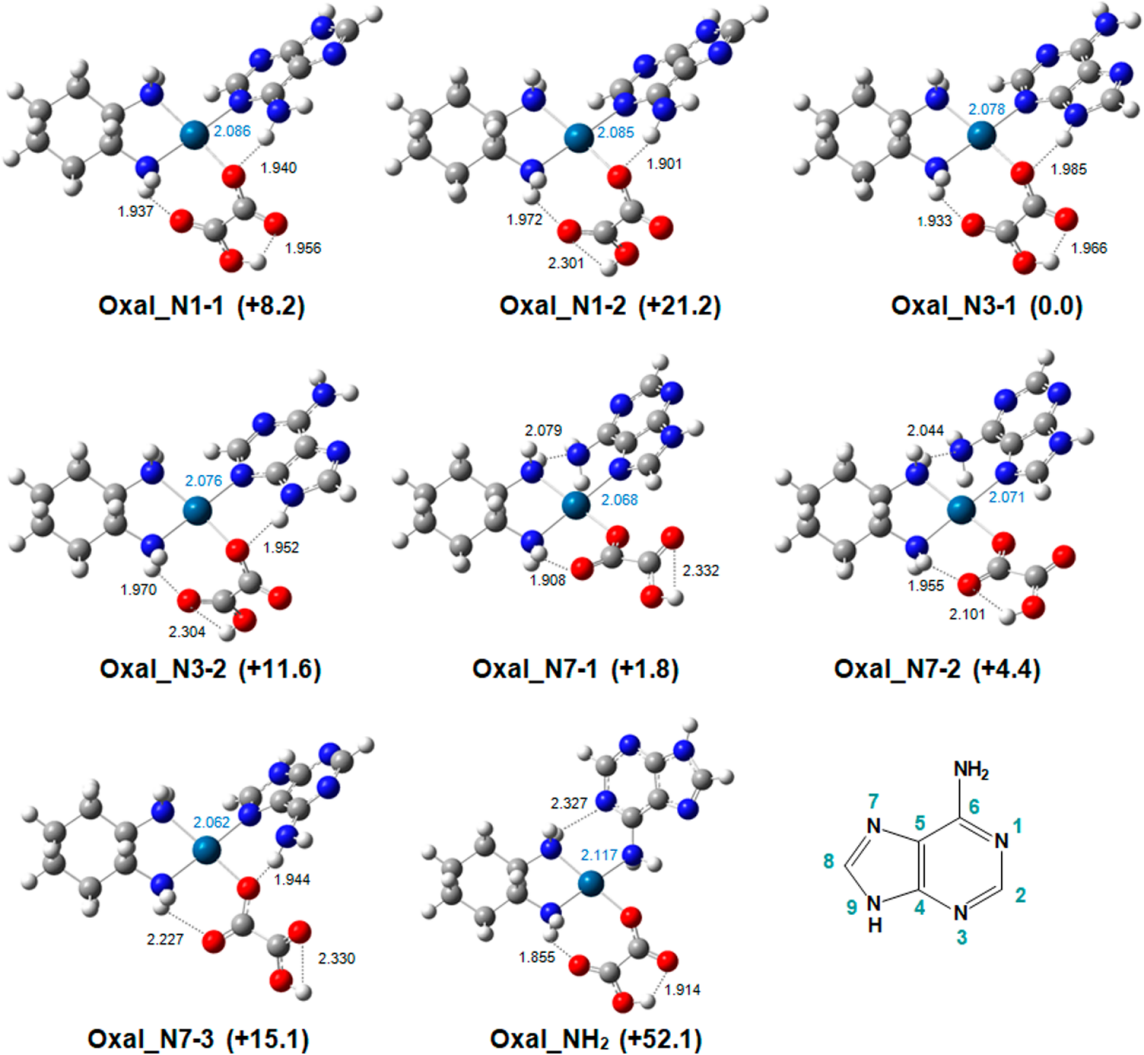
Representative structures computed for the [OxaliPt+H+A]^+^ complex in which platinum binds directly to adenine. Bond lengths are given in Å; relative free energies are given in kJ mol^–1^ with respect to **Oxal_N3–1**.

Based on previous IRMPD results,^8a,9b^ where we found that sampled cisplatin-adenine complexes are mainly formed by two isomers, with platinum binding adenine at N3 in the major species and at N1 in the second one, we first focused the theoretical survey on structures where the metal directly binds adenine at either the N3 or N1 positions, and the protonation occurs on one of the oxygen atoms. Furthermore, isomers characterized by a direct platinum–adenine bond at the N7 or NH_2_ position were also considered. Figure 1 displays representative optimized geometries of [OxaliPt+H+A]^+^ obtained following the aforementioned criteria.

The labels used henceforth to describe the different geometries consist of three parts: (i) the name of the Pt drug (Oxal/Carbo), (ii) the platinum binding site of adenine, and (iii) an additional number to distinguish different forms or conformations of a particular isomer. Other optimized geometries are reported in the Supporting Information (Figure S5).

In all of the structures considered, platinum maintains a square planar coordination scheme, as already reported for [OxaliPt+H+G]^+^. Different platination sites allow the formation of distinct arrangements between adenine and oxaliplatin. In particular, platination at either the N3 or N1 positions favors H-bonding between the adenine hydrogen donors N9–H or NH_2_, respectively, and the oxalate acceptor. Platination at the N3 position of adenine yielded the most stable isomer, **Oxal_N3–1**. This structure is characterized by the presence of three hydrogen bonds. The first one is established between the two COO groups of the protonated oxalate moiety. The oxalate ligand is also H-bonded to an amino group of the DACH cycle and with the N9–H bond of adenine. These three weak interactions lead to a stiffening of the structure. Among conformers characterized by metal coordination at N7, **Oxal_N7–1** and **Oxal_N7–2** lie only 1.8 and 4.4 kJ mol^–1^ higher in relative free energy, respectively, and both present the adenine amino group donor H-bonded with the DACH amino group acceptor. The lowest-energy N1-platinated structure, **Oxal_N1–1**, is located at 8.2 kJ mol^–1^ relative to the global minimum, while the binding of the metal to the adenine amino group, **Oxal_NH**_**2**_ (52.1 kJ mol^–1^), is energetically less favorable.

**Figure 2:**
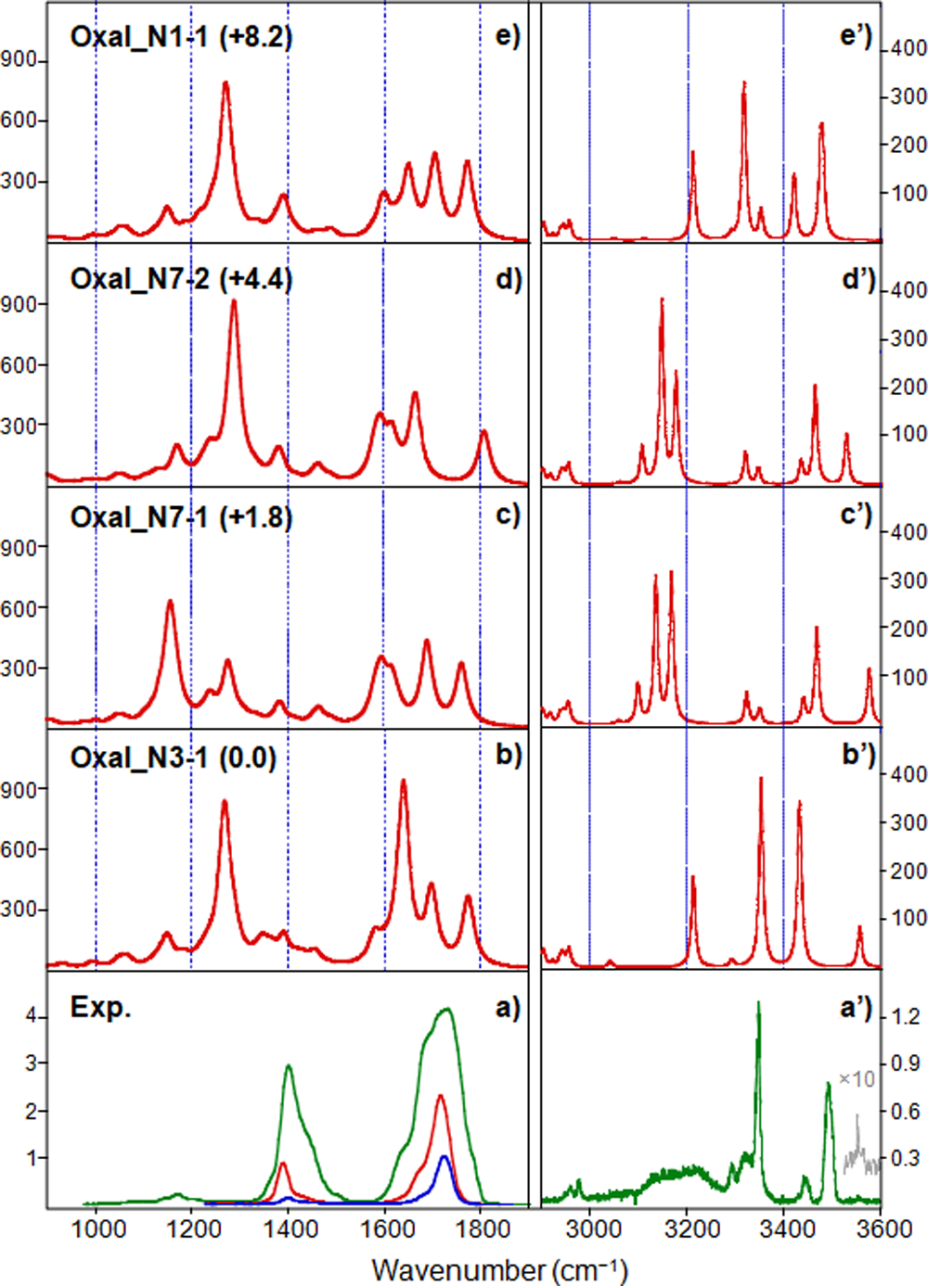
Experimental IRMPD spectrum of the [OxaliPt+H+A]^+^ complex (lower panel) in both the (a) fingerprint range (irradiation time of 300 ms, no attenuation in the green trace, or with the use of one or two attenuators, in the red and blue traces, respectively) and (a’) X–H range with an irradiation time of 1 s, compared with the computed IR spectra (b–e) and (b’**–**e’) are reported for structures representative of different binding schemes.

The IRMPD spectra of mass-selected [OxaliPt+H+A]^+^ ions (*m*/*z* 532/534) were recorded by using two different experimental platforms in both the mid-IR (950–1900 cm^–1^) and X-H (C,N,O) stretching (2900–3600 cm^–1^) ranges. As observed by the CID assay, the formation of an ion at *m*/*z* 397 by release of neutral adenine is the exclusive photofragmentation channel in both the explored regions, in contrast with the low propensity of [OxaliPt+H+G]^+^ to lose the neutral nucleobase. Experimental IRMPD spectra of [OxaliPt+H+A]^+^ are plotted in Figure 2a/a’ together with the IR spectra calculated for species representative of the different binding schemes (Figure 2b/b’–e/e’).

In the fingerprint range, the experimental spectrum is characterized by two prominent, broad signals (∼180 cm^–1^ fwhm). The major one spans from 1600 to 1790 cm^–1^, with a shoulder at approximately 1640 cm^–1^ and a maximum centered at 1720 cm^–1^. The second one appears at 1338/1556 cm^–1^, with the maximum centered at 1388 cm^–1^ and a shoulder at 1441 cm^–1^. Both signals presumably result from a combination of several absorptions. Some small features at around 1140, 1172, 1252, and 1297 cm^–1^ are also detected. Inspection of Figure 2 points out that none of the computed structures match the experimental spectrum in the fingerprint range. In fact, while the experimental feature centered at 1720 cm^–1^ could be approximately explained by the combination of the calculated IR bands of all reported isomers, the IRMPD band centered at 1388 cm^–1^ is in no way reproduced in the computed spectra. Also noteworthy is the absence in the experimental spectrum of detectable bands where intense absorptions are expected at 1268, 1270, or 1288 cm^–1^ for **Oxal_N3–1, Oxal_N7–1,** and **Oxal_N1–2,** respectively, due to OH-bending and C–O(Pt)-stretching vibrational modes of the protonated oxalate group, (PtO)O=C–C=O(OH). Similarly, the high-energy range of the IRMPD spectrum (Figure 2a’) does not relate with the computed spectra of the structures reported in Figure 2b’–e’, even if the contribution of different isomers in the sampled ionic complex is assumed.

**Figure 3:**
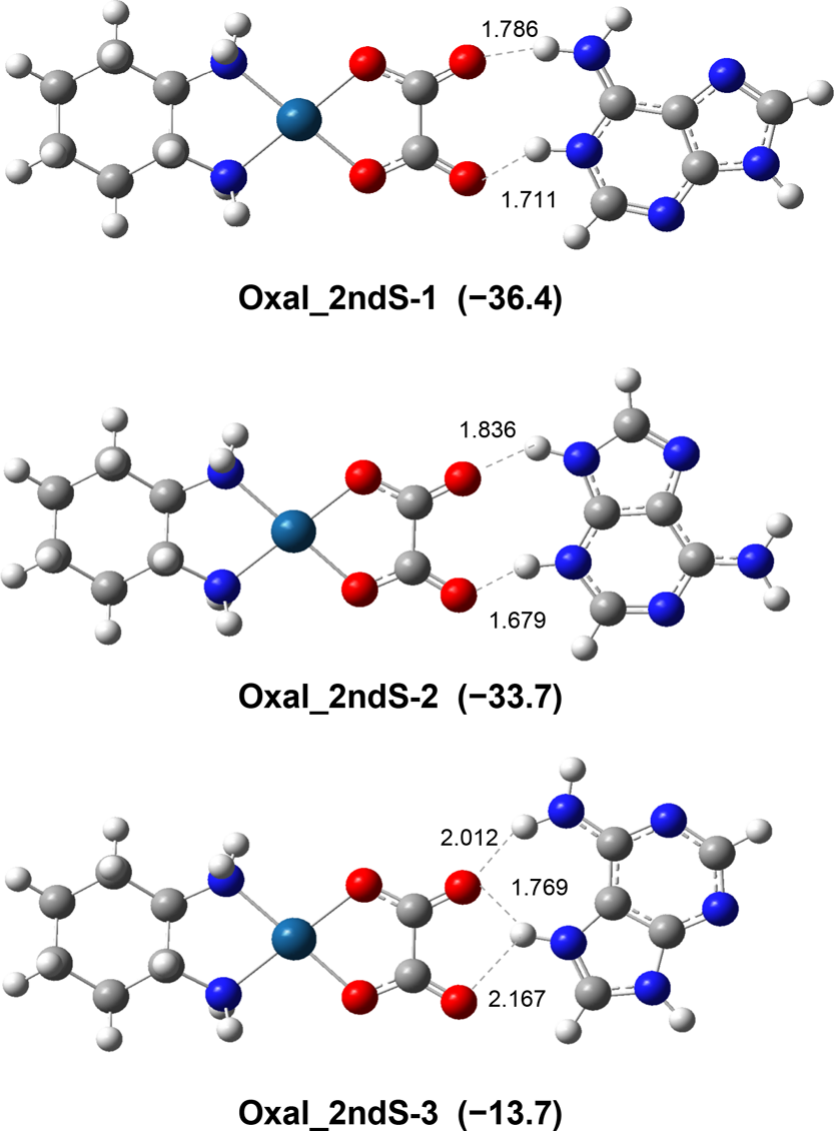
Optimized geometries of the lowest-energy second-shell structures for the [OxaliPt+H+A]^+^ complex. Bond lengths are given in Å. The relative free energies (in kJ mol^–1^) are reported with respect to **Oxal_N3–1**.

In consideration of the poor match that emerged from the spectral comparison (Figure 2), as well as of the distinct (photo)fragmentation behavior by loss of neutral adenine, other geometries with different binding motifs were envisaged. In these so-called ≪ second-shell ≫ structures, the nucleobase does not directly interact with the metallic center but is noncovalently bound to the ligand(s) in the first coordination sphere of Pt. Remarkably, the initial protonation of oxaliplatin was almost systematically followed by a proton transfer toward adenine during the geometry optimization. The optimized geometries of low-lying ≪ second-shell ≫ structures of [OxaliPt+H+A]^+^ are given in Figure 3. Surprisingly, all of these complexes were found to be more energetically stable than **Oxal_N3–1**. In all of the geometries, protonation on adenine allows a dual H-bond interaction with oxygen atoms of the two carboxyl groups of the oxaliplatin. In the (new) global minimum **Oxal_2ndS-1**, the N1 position of adenine is protonated and this proton forms a strong H-bond with one of the oxygens of the dicarboxylato ligand, which is also involved in a second H-bond with the adenine NH_2_ as a donor. This proton-bound complex is 36.4 kJ mol^–1^ more stable than **Oxal_N3–1**. The **Oxal_2ndS-2** complex lies only 2.7 kJ mol^–1^ higher in relative energy compared to **Oxal_2ndS-1** and presents protonated N3 and N9–H donors hydrogen-bonded with the oxalato ligand. Interestingly, the energy gap between **Oxal_2ndS-1** and **Oxal_2ndS-2** is practically identical to that calculated for the two most stable proton-bound adenine dimers protonated in N1 and N3.^25^ In **Oxal_2ndS-3,** 22.7 kJ mol^–1^ above **Oxal_2ndS-1**, the extra proton located onto N7 is engaged in a bifurcated hydrogen-bonding motif with the oxalate oxygens, one of which benefits from a third H-bond with the adenine NH_2_ donor. In these three structures, adenine and oxaliplatin moieties lie in the same plane, and adenine interacts with the two nonmetal-bonded carboxylate groups. When the adenine amino group interacts with one oxygen atom coordinated to Pt, while keeping the same N1H interaction described in **Oxal_2ndS-1**, the stability of the ensuing complex (**Oxal_2ndS-4**, Figure S5) decreases by 44.2 kJ mol^–1^ relative to the new global minimum.

**Figure 4:**
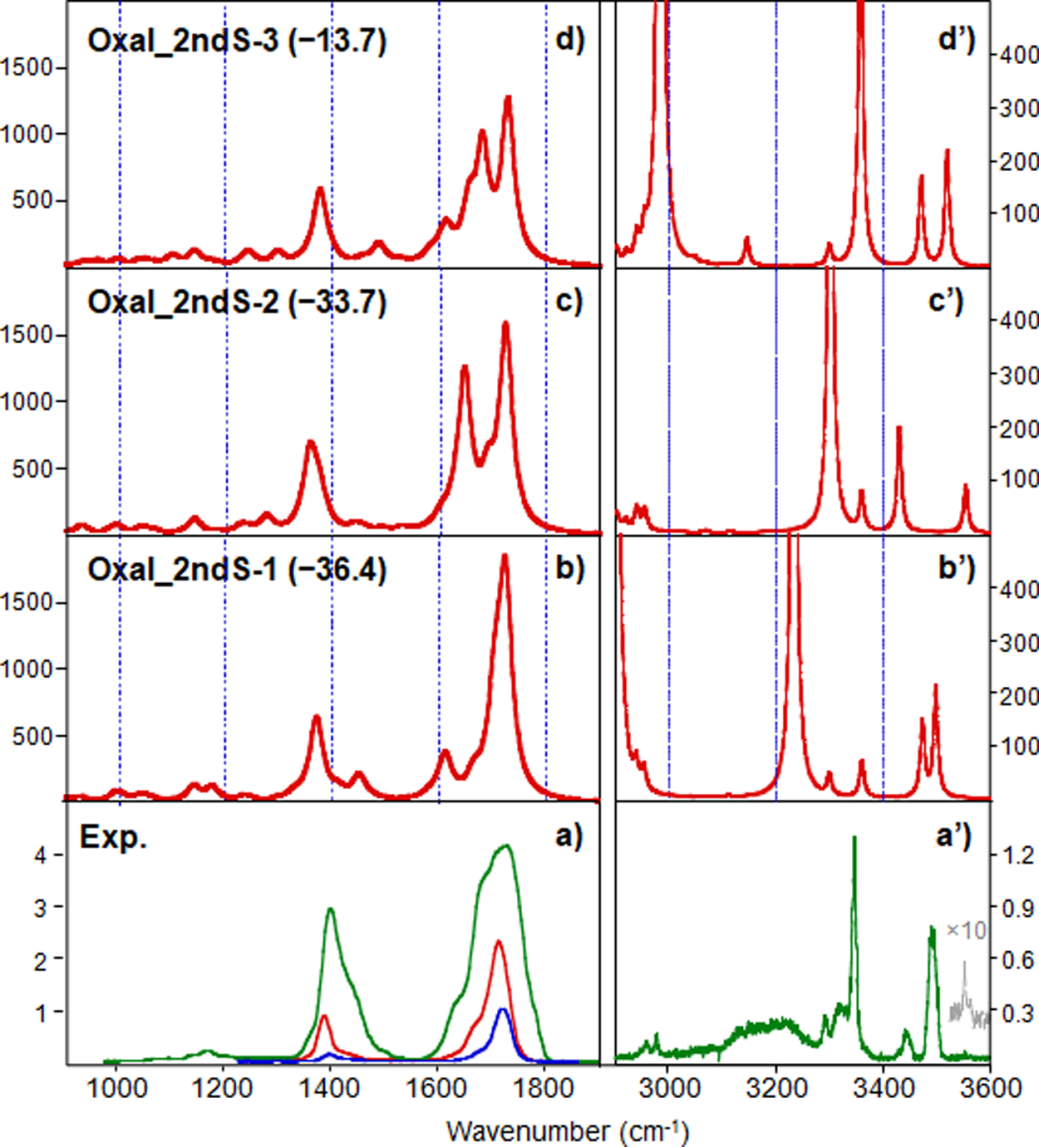
Experimental IRMPD spectrum of the [OxaliPt+H+A]^+^ complex (lower panel) in both the (a) fingerprint range (irradiation time of 300 ms, no attenuation in the green trace, or with the use of one or two attenuators, in the red and blue traces, respectively) and (a’) X–H range with an irradiation time of 1 s. Computed IR spectra (b–d) and (b’–d’) are reported for second-shell geometries. See the text for details.

The comparison between the experimental IRMPD spectra of [OxaliPt+H+A]^+^ and the computed IR spectra of these lowest-energy second-shell structures is presented in Figure 4. A good agreement appears in the fingerprint range when considering the contribution of both close low-lying isomers **Oxal_2ndS**-1 and **Oxal_2ndS-2**, although the presence of **Oxal_2ndS-3** in the sampled adenine-oxaliplatin complex cannot be excluded spectroscopically.

Accordingly, the vibrational modes of [OxaliPt+H+A]^+^ can be interpreted as detailed in Table S2, which reports the main experimental IRMPD features and the IR bands calculated for **Oxal_2ndS-1, Oxal_2ndS-2,** and **Oxal_2ndS-3.** In particular, the shoulder observed at 1640 cm^–1^ can be associated with the NH_2_ scissoring mode of either DACH or adenine, and the maximum at 1720 cm^–1^ is due to the oxalate C=O stretching modes. The broad absorption centered at 1388 cm^–1^ can be ascribed to the contribution of several C–H and N–H bending modes of adenine and DACH and to CC and CN stretching modes of adenine and the oxalate ligand. The IRMPD spectrum recorded in the X-H stretching range confirms the presence of the three second shell proton-bound adducts in the sampled [OxaliPt+H+A]^+^ complex. In particular, the two features at ca. 3430 and 3553 cm^–1^ predicted only in the computed spectrum of **Oxal_2ndS-2** (3430 and 3554 cm^–1^) are attributed to the symmetric and asymmetric NH_2_ stretching modes of adenine, whereas the broad absorption centered at 3491 cm^–1^ corresponds to the contribution of two H-stretching modes: N9–H and NH_2_ of the free H (not involved in the H-bond with oxalate) observed only in the computed spectra of **Oxal_2ndS-1** and **Oxal_2ndS-3** isomers, and expected at 3473 and 3498 cm^–1^ for the former and at 3471 and 3520 cm^–1^ for the latter. It is worth noting that these features were also observed in the IRMPD spectra of the proton-bound dimers of adenine reported in the literature as further support for the presence of second-shell complexes.^25^ Another significant aspect of these proton-bound species concerns the protonation site of adenine. In a previous IRMPD study on protonated adenine, it was shown that protonation is predominantly directed at the N1 position of the *N9H* tautomer.^26^ The formation of the noncovalent adduct with oxaliplatin, instead, seems to make the N1, N3, and N7 positions of adenine equally predisposed to proton transfer from the oxalate moiety.

### 3.3 IRMPD Spectroscopy of the [CarboPt+H+A]^+^ Complex

**Figure 5:**
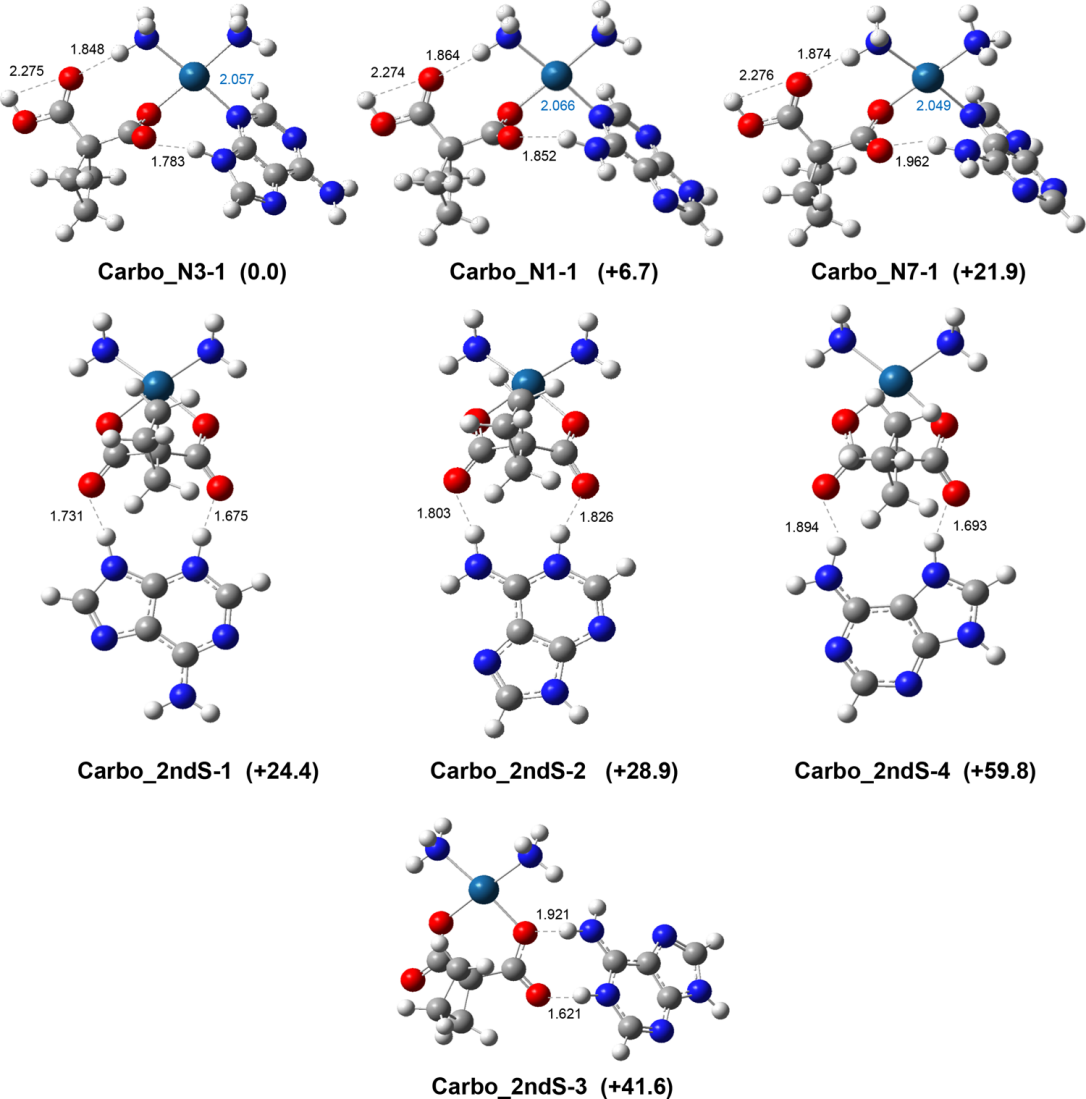
Representative structures computed for the [CarboPt+H+A]^+^ complex. Bond lengths are given in Å; relative free energies in kJ mol^–1^ are reported with respect to **Carbo_N3–1**.

Given the results obtained for [OxaliPt+H+A]^+^, computational investigation of the [CarboPt+H+A]^+^ complex was carried out by considering both the platinum-bound and second-shell complexes. The optimized geometries of the lowest energy conformers, representative of different binding schemes, are reported in Figure 5. Additional conformers can be found in the SI (Figure S5).

Unlike what was just observed for [OxaliPt+H+A]^+^, the platinum-bound geometries are found to be lower in energy than second-shell complexes. The former structures are protonated on a carboxylate group of carboplatin, and protonation results in the weakening of the Pt–O bond, promoting the direct interaction of platinum with neutral adenine. In the global minimum, **Carbo_N3–1**, the platinum atom, binds to adenine at the N3 center, favoring the formation of a H-bond between the N9–H donor and the carboxylate acceptor still bound to the platinum atom. **Carbo_N1–1**, at 6.7 kJ mol^–1^ of relative free energy compared to **Carbo_N3–1**, and **Carbo_N7–1**, at 21.9 kJ mol^–1^, are characterized by a Pt–adenine interaction onto the N1 and N7 positions, respectively, and are stabilized by H-bonding between the adenine NH_2_ group and the COO group coordinated to the metallic center. Regarding the second-shell geometries, as in the case of [OxaliPt+H+A]^+^, the entering proton moved onto adenine, allowing a proton-bound interaction with either one or two carbonyl oxygen atoms of the carboplatin malonate moiety. Protonation at N3 yields the most stable second-shell adduct, **Carbo_2ndS-1**, at 24.4 kJ mol^–1^, characterized by an additional H-bond between the N9–H donor and the carbonyl oxygen of the second malonic carboxylate. When protonation takes place at N1 (**Carbo_2ndS-2**), the second-shell form is found slightly less stable lying at 28.9 kJ mol^–1^. Again, hydrogen bonds are established with each carboxylate group involving the N1 center and the amino group of adenine. The **Carbo_2ndS-2/Carbo_2ndS-3** comparison illustrates the situation when the same positions of adenine interact with either two or one carboxylate group. The interaction with a single carboxylate group (**Carbo_2ndS-3**) is energetically disfavored, as already observed with oxaliplatin (*vide supra*). Finally, in **Carbo_2ndS-4**, at 59.8 kJ mol^–1^, the proton transfer occurs onto N7 and both carbonyl groups of carboplatin interact with protonated adenine, one with the proton in N7 and the second with the NH_2_ group. This interaction mode turned out to be less favorable.

**Figure 6:**
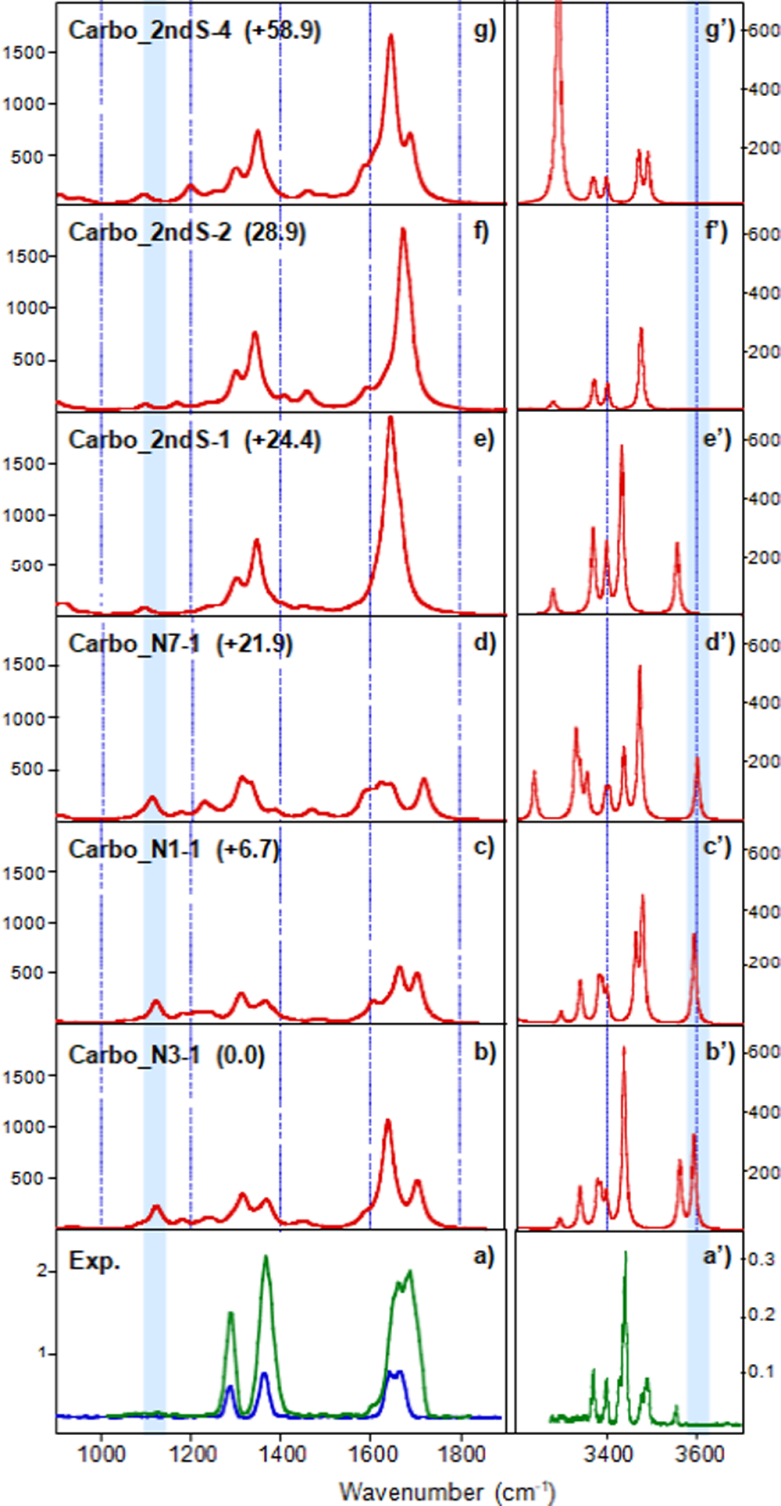
Experimental IRMPD spectrum of the [CarboPt+H+A]^+^ complex (lower panel) in both the (a) fingerprint range (irradiation time of 500 ms, green trace, or 180 ms, blue trace) and (a’) X–H range with an irradiation time of 1 s, compared with the computed IR spectra (b–g) and (b’–g’) of selected geometries reported in Figure 5. See the text for details.

Figure 6 shows the comparison of the experimental spectrum of [CarboPt+H+A]^+^ with the computed IR spectra of the isomers presented in Figure 5. In the fingerprint range, the experimental spectrum presents three prominent absorptions, the first two at 1288 and 1369 cm^–1^ and the broadest one centered at 1675 cm^–1^, which is preceded by a shoulder at 1611 cm^–1^. Unexpectedly, despite being energetically disfavored, the second-shell structures **Carbo_2ndS-1/2ndS-2/2ndS-4** provide a better match with the experimental spectrum compared to the products of substitution (Figure 6). On the other hand, a significant contribution of **Carbo_2ndS-3** can be excluded based on the comparison with the experimental spectrum reported in Figure S6. It is worth noting the absence in the experimental spectrum of the C–OH stretching mode of the protonated carboxylate group of the 1,1-cyclobutanedicarboxylato moiety, expected at 1122 cm^–1^ (highlighted in blue in Figure 6) for the three platinum-bound geometries, **Carbo_N1–1**, **Carbo_N3–1,** and **Carbo_N7–1.** Since such a vibrational mode is a signature of protonation on the carboplatin ligand, this result suggests that protonation rather occurs on adenine. Further confirmation of this assumption is obtained by comparing the experimental and computed spectra in the X–H stretching range. The IRMPD spectrum of [CarboPt+H+A]^+^ presents several features at 3300, 3369, 3398, 3426–3440, 3477, 3491, and 3554 cm^–1^, but not the one predicted at 3588 cm^–1^ for **Carbo_N1–1**, **Carbo_N3–1,** and **Carbo_N7–1**, which corresponds to the O–H stretching of the protonated carboxylate group (highlighted in blue in Figure 6). Instead, again the presence of the multiple noncovalent forms **Carbo_2ndS-1, Carbo_2ndS-2,** and **Carbo_2ndS-4** may nicely reproduce the features of the IRMPD spectrum of [CarboPt+H+A]^+^ in both spectral ranges.

According to this comparison, the vibrational modes of [CarboPt+H+A]^+^ have been interpreted as described in Table S3, which reports both the experimental IRMPD features and the IR bands calculated for **Carbo_2ndS-1**, **Carbo_2ndS-2,** and **Carbo_2ndS-4**. The band centered at 1288 cm^–1^ can be associated with the umbrella mode of both ammonia ligands, calculated at 1286 and 1301 cm^–1^ for **Carbo_2ndS-4**, 1297 and 1300 cm^–1^ for **Carbo_2ndS-2**, and at 1300–1304 cm^–1^ for **Carbo_2ndS-1.** The C–O–Pt stretching mode predicted at 1343 and 1348 cm^–1^ for **Carbo_2ndS-2** and **Carbo_2ndS-4**, respectively, and combined with the bending C2–H expected at 1345 and 1348 cm^–1^ for **Carbo_2ndS-1,** can be associated with the intense feature between 1330 and 1408 cm^–1^ and centered at 1369 cm^–1^. The broad absorption centered at 1675 cm^–1^ can be assigned to several intense vibrational modes including the NH_2_ scissoring mode associated with the C6-NH_2_ stretching mode computed at 1645 cm^–1^ (**Carbo_2ndS-1**) and 1646 cm^–1^ (**Carbo_2ndS-4**); the combined C=O stretching modes predicted at 1666 cm^–1^ (**Carbo_2ndS-1**) and 1671 cm^–1^ (**Carbo_2ndS-2**); the N1–H bending mode engaged in the proton-bound complex with carboplatin, expected at 1686 cm^–1^ (**Carbo_2ndS-2**); and the C=O stretching mode involved in the H-bond with NH_2_ of adenine computed at 1690 cm^–1^ (**Carbo_2ndS-4**). The shoulder at 1611 cm^–1^ may be mostly associated with the adenine NH_2_ scissoring in **Carbo_2ndS-4** (1583 cm^–1^) and to the NH_2_ scissoring mode of NH_3_ calculated at 1606 and 1610 cm^–1^ for **Carbo_2ndS-4** and at 1615 and 1617 cm^–1^ for **Carbo_2ndS-1**.

In the X-H stretching range, the two features at ca. 3426–3440 and 3554 cm^–1^ can be attributed to the symmetric and asymmetric NH_2_ stretching modes of adenine predicted at 3428 and 3551 cm^–1^ for **Carbo_2ndS-1.** The vibrational stretching modes of the N9–H and of the free H of NH_2_ are associated with the band observed at 3477 cm^–1^ and calculated at 3470 and 3471 cm^–1^ for **Carbo_2ndS-2**. This experimental signal may also be associated with the N9–H stretch of **Carbo_2ndS-4** computed at 3467 cm^–1^. Other bands observed at 3369 and 3398 cm^–1^ are assigned to the asymmetric NH_3_ stretches of the three forms, as reported in Table S3. The remaining experimental band detected at 3491 cm^–1^ suggests the contribution of **Carbo_2ndS-4** due to the absence of any detectable features in this part of the spectrum for **Carbo_2ndS-1** and **Carbo_2ndS-2** and can be associated with the vibrational stretching mode of the free H of NH_2_ computed at 3487 cm^–1^.

To simulate the attack of adenine to protonated carboplatin [CarboPt+H]^+^, we carried out a series of mod-redundant scans at the B3LYP/6-311G** level. Two scenarios were considered. In the first one, the attack of adenine occurs directly on the platinum center. We assumed an initial approach of adenine below the square planar environment of platinum. Results are summarized in Figures S7 and S8 of the Supporting Information, as two initial orientations of adenine were considered. These figures show that the reduction of the Pt······N3 distance leads to a progressive and significant increase in destabilization of the system. At a short Pt······N3 distance, the Pt–O=C(OH) is cleaved. A further decrease in the Pt······N3 distance defines an activation barrier, in both cases above 95 kJ mol^–1^, as it induces an important reorganization around Pt, finally leading to “Carbo-N3” structures. In the second scenario (Figure S9), we considered the approach of adenine toward the carboxylate groups of the 1,1-cyclobutanedicarboxylato moiety, which is associated with a proton transfer toward adenine. In this case, the system is continuously stabilized and ultimately evolves into “second-shell”-like structures. This last pathway is kinetically favored with no activation barrier. The fact that experimentally we presently exclusively observed second-shell structures with CarboPt would suggest that the reaction of [CarboPt+H]^+^ with adenine is under kinetic control.

As additional support for these findings, we have performed CID experiments on both [CarboPt+H+A]^+^ and [OxaliPt+H+A]^+^ complexes mass-isolated from solutions where the reactants are allowed to interact for 7 days after mixing. The CID experiments were conducted with two different MS instruments: the LTQ-XL as previously, and with the ESI-FT-ICR where the ions are accumulated in the quadrupole and the major peaks of the clusters are mass-isolated. The results are reported in Figure S10a,b for [CarboPt+H+A]^+^ and Figure S11a,b for [OxaliPt+H+A]^+^. Even after an incubation time of 7 days, no changes were observed in the CID spectra of the complexes, both exclusively showing the loss of adenine. These provide clear evidence that only second-shell adenine adducts are formed in our solutions.

### 3.4 Does Guanine Also Form Second-Shell Complexes with Carboplatin and Oxaliplatin?

Considering the somewhat unexpected results obtained with adenine, an additional computational investigation of the [CarboPt+H+G]^+^ and [OxaliPt+H+G]^+^ complexes was performed in order to verify if we had underestimated the presence of second-shell complexes in our previous report.^10^Figure S12 displays the most stable second-shell forms computed for the [CarboPt+H+G]^+^ and [OxaliPt+H+G]^+^ species, together with the geometries of the most stable N7-platinated complexes previously reported. Unlike adenine, these second-shell forms turn out to be markedly less stable than the N7 platinum-bound geometries: **Oxal_G_2ndS-1** and **Carbo_G_2ndS-1** are indeed calculated to be 40.4 and 62.5 kJ mol^–1^ higher in energy than their respective global minimum. In the case of carboplatin, another relevant feature concerns the protonation site of the second-shell complexes. Despite the higher proton affinity of guanine as compared to adenine,^27^ in the most stable geometries obtained for the [CarboPt+H+G]^+^ complex, the proton remains located on the carboplatin moiety as in the cases of **Carbo_G_2ndS-1** and **Carbo_G_2ndS-2**.

Figures S13 and S14 show the IRMPD spectrum of the [CarboPt+H+G]^+^ and [OxaliPt+H+G]^+^ recorded in the fingerprint range, as reported earlier,^10^ and presently compared to the most stable second-shell structures and to their respective global minimum. The IRMPD spectra of [CarboPt+H+G]^+^and [OxaliPt+H+G]^+^ match well with the vibrational spectra calculated for the global minimum structures **Carbo G_N7–1,Oxal_G_N7–1,** and **Oxal_G_N7–2**, characterized by a direct complexation of Pt onto the N7 position of guanine, excluding at the same time any contribution of the second-shell forms in the sampled ionic population.

## 4 Conclusions

Our previous results obtained on the cisplatin-adenine complex were characterized by a slow isomerization within the complex highlighted by the inversion of the intensities of two experimental bands, noted by comparing the IRMPD spectra of the *cis*-[Pt(NH_3_)_2_(A)Cl]^+^ complex isolated from two solutions, one prepared 24 h before the analysis and the other 2 weeks before.^8a^ Once again, interactions between adenine and platinum-based drugs, i.e., oxaliplatin and carboplatin, reported in this paper, result in a rather unexpected behavior. [OxaliPt+H+A]^+^ and [CarboPt+H+A]^+^ adducts were generated in solution by following the same procedure adopted for the preparation of the corresponding complexes with guanine, namely, [OxaliPt+H+G]^+^ and [CarboPt+H+G]^+^. These adducts were then analyzed by electrospray ionization tandem mass spectrometry (ESI-MS/MS). However, in contrast to the MS/MS spectra of the guanine complexes, the CID spectra of [CarboPt+H+A]^+^ and [OxaliPt+H+A]^+^ complexes show only one product ion obtained by the loss of neutral adenine. Structural characterization of the adenine complexes was performed by combining IRMPD action spectroscopy with quantum chemical calculations. Interestingly, comparisons between experimental and calculated IR spectra reveal that in both the sampled [CarboPt+H+A]^+^ and [OxaliPt+H+A]^+^ complexes there is no direct coordination of the platinum atom to the nucleobase, as previously observed for cisplatin/adenine or guanine complexes and for the guanine complexes of CarboPt and OxaliPt. The IRMPD spectra of [OxaliPt+H+A]^+^ indeed indicate the presence of three proton-bound adducts in the sampled ionic population, in which protonated adenine at the N1, N3, or N7 position forms H-bonds with the oxalate ligand of oxaliplatin. These second-shell complexes are found to be significantly more stable than those arising from a direct coordination of the platinum atom at the N3, N1, or N7 position of adenine. Conversely, DFT calculations predict for the [CarboPt+H+A]^+^ complex a lower stability of the external coordination adducts compared with the geometries presenting direct platinum–adenine coordination. Nevertheless, its IRMPD spectra again point to the almost exclusive presence of three proton-bound isomers in the ionic population of [CarboPt+H+A]^+^. Similar types of complexes are therefore observed with both drugs, which are consistent with the loss of adenine during CID experiments. Consequently, it is clear that the binding motifs of the sampled adenine complexes with carboplatin and oxaliplatin are remarkably different from that observed with cisplatin or in the presence of guanine, both characterized by direct platination on the nucleobase. As a possible explanation, the presence of bulkier substituents in the platinum coordination sphere of carboplatin and oxaliplatin shields the platinum atom and prevents the easy formation of a platinum–adenine bond. This tendency further evidences the intrinsic lower affinity of platinum for adenine, as previously observed in the CID experiments on cisPt/A and cisPt/G complexes.^8a^ The results presently obtained are consistent with the lower predisposition of the platinum-based drugs to form 1,2-d(ApG) cross-links with DNA during their pharmacological activity.

As a final note, we were able, for the first time in this study, to isolate and characterize the encounter complexes formed by a platinum-based drug and a nucleobase. In previous studies, we had already reported the structural characterization of such elusive intermediates resulting from the reaction of the active aqua complex of cisplatin, cis-[PtCl(NH_3_)_2_(H_2_O)]^+^, with amino acids such as methionine^28^ and cysteine^29^ or with other biological ligands,^30^ but never with nucleotides or nucleobases.

These results once again demonstrate the relevance of the CID/IRMPD/DFT combination for the structural characterization of simple gaseous ionic complexes. This strategy is an effective tool to clarify and predict the reactivity and affinity of platinum agents with biomolecules in general and particularly with DNA nucleotides.

## Supporting Information Available

Detailed experimental mass spectra, CID, optimized geometries, and IRMPD spectra of ions under examination; tables with experimental and computed vibrational bands for relevant structures of [OxaliPt+H+A]^+^ and [CarboPt+H+A]^+^ ions; and Cartesian coordinates of all of the computed structures and complete ref (21) (PDF)
