# Supplementary material for: Encounter Complex of Adenine with Carboplatin and Oxaliplatin Anticancer Drugs Elucidated by IRMPD Spectroscopy and Theoretical Study
Source: Inorg Chem. 2025 Mar 6;64(10):4873–83. doi: 10.1021/acs.inorgchem.4c04731 (PMC11920941; doi:10.1021/acs.inorgchem.4c04731)
Supplement: Supplementary file 1 — ic4c04731_si_001.pdf [file ic4c04731_si_001.pdf]

Encounter Complex of Adenine with Carboplatin and Oxaliplatin Anticancer Drugs Elucidated by IRMPD Spectroscopy and Theoretical Study

Barbara Chiavarino[1], Lucretia Rotari[1], Maria Elisa Crestoni[1], Davide Corinti[1], Debora Scuderi[2] and Jean-Yves Salpin[3*]*

1) Dipartimento di Chimica e Tecnologie del Farmaco, Università di Roma "La Sapienza", P.le A. Moro 5, I-00185 Roma, ITALY

2) Université Paris-Saclay, CNRS, Institut de Chimie Physique, 91405, Orsay, France.

3) Université Paris-Saclay, Univ Evry, CY Cergy Paris Université, CNRS, LAMBE, 91025, Evry-Courcouronnes, France

Corresponding authors:

Dr Barbara Chiavarino
e-mail: barbara.chiavarino@uniroma1.it

Dr Jean-Yves Salpin
e-mail: jeanyves.salpin@univ-evry.fr

SUPPORTING INFORMATION: a total of 64 pages

Figure S1 (a). Full scan positive-ion ESI mass spectrum of a 5 µM oxaliplatin and dAMP (1:1) mixture in a water/methanol (1:1) solution. **(b).** High resolution ESI FT-ICR mass spectrum of [OxaliPt+H+A]$^+$ ion (in red) compared to the calculated isotopic pattern for [$C_{13}H_{20}N_7O_4Pt$] (in black).

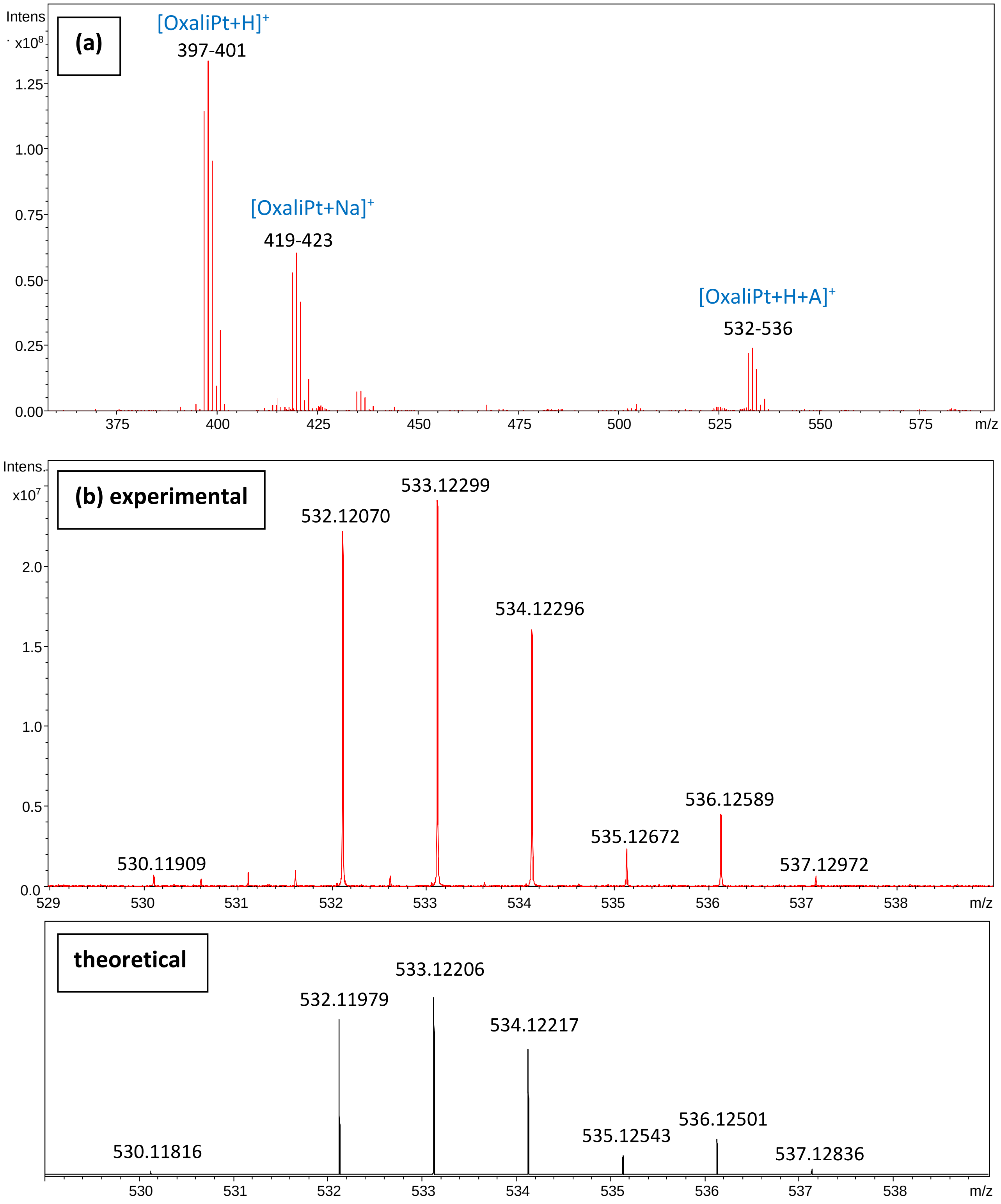

Figure S2 (a) Full scan positive-ion ESI mass spectrum of a 5 μM carboplatin and dAMP (1:1) mixture in a water/methanol (1:1) solution **(b)** High resolution ESI FT-ICR mass spectrum of $[CarboPt+H+A]^+$ ion (in red) compared to the calculated isotopic pattern for $[C_{11}H_{18}N_7O_4Pt]$ (in black).

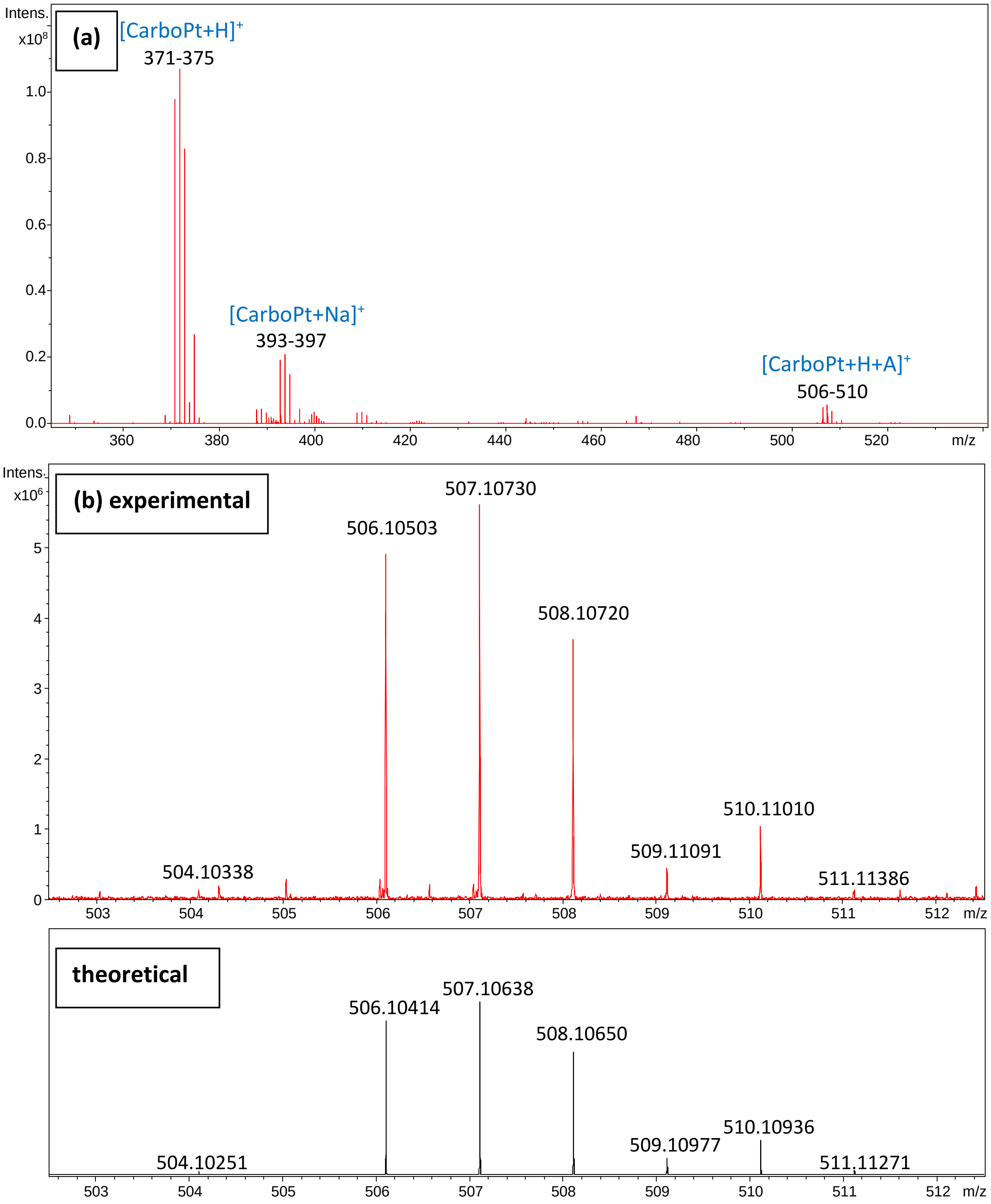

Figure S3. CID mass spectrum of: **(a)** [OxaliPt+H+A]$^+$ ion recorded at CE 10 eV (upper pannel) and **(b)** [OxaliPt+H+G]$^+$ ion recorded at CE 12 eV (see ref 10 for more details) (lower pannel) .

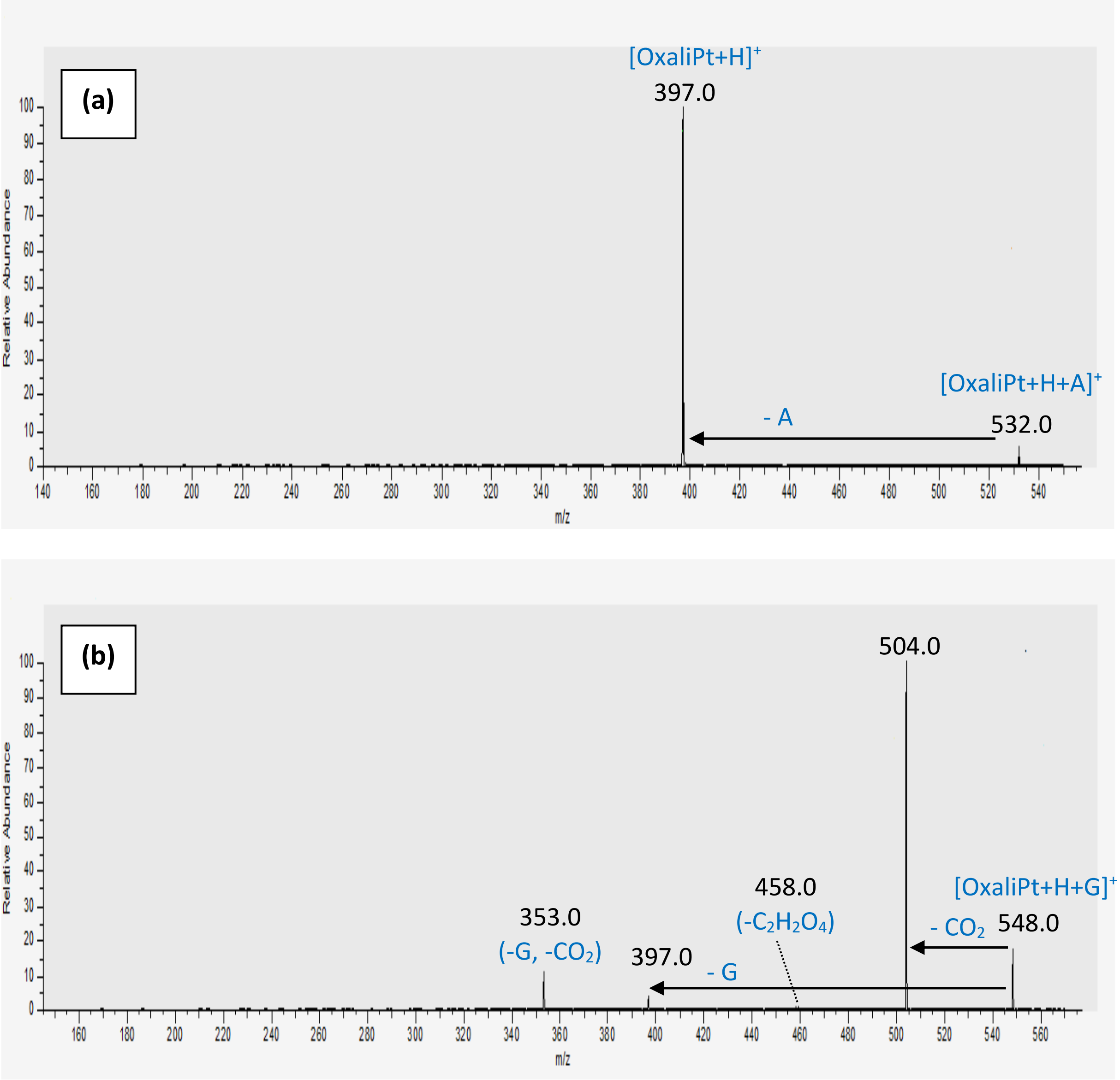

Figure S4. CID mass spectrum of **(a)** [CarboPt+H+A]$^+$ ion recorded at CE 10 eV (upper pannel) and **(b)** [CarboPt+H+G]$^+$ ion recorded at CE 12 eV (see ref 10 for more details) (lower pannel).

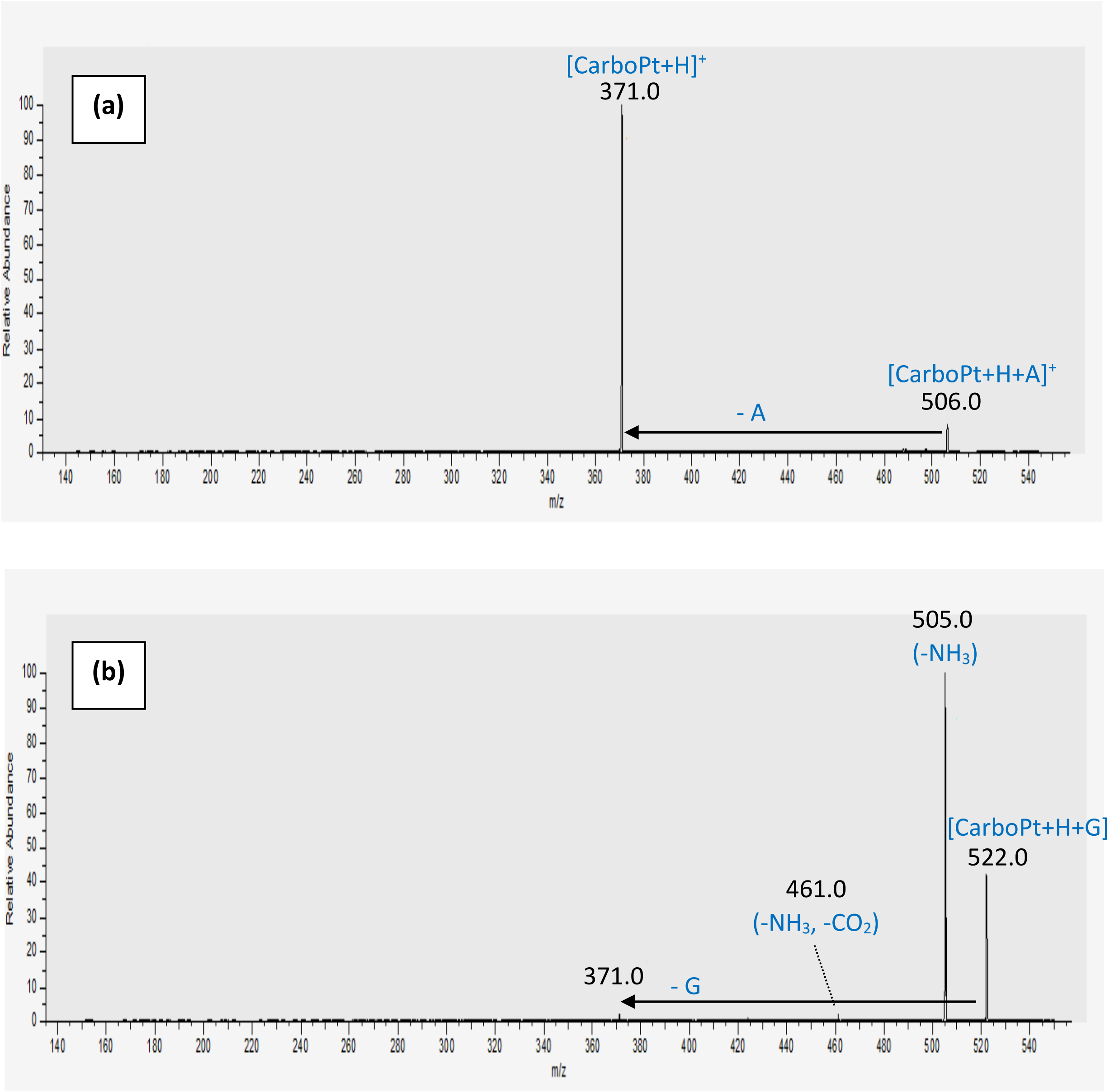

Figure S5. Additional structures computed for the $[CarboPt{+}H{+}A]^{+}$ and $[OxaliPt{+}H{+}A]^{+}$ systems. Relative free energies in kJ mol^{-1}are reported with respect to **Carbo_N3-1** and **Oxal_N3-1**.

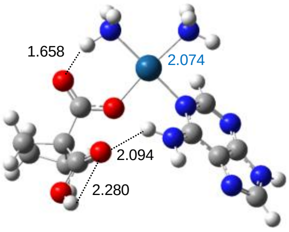

Carbo_N1-2 +13.4

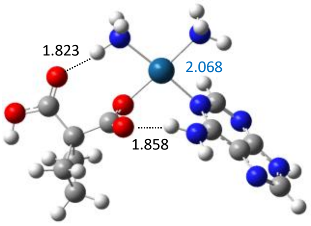

Carbo_N1-3 + 29.7

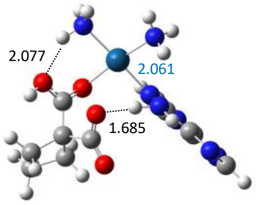

Carbo_N1-4 +160.5

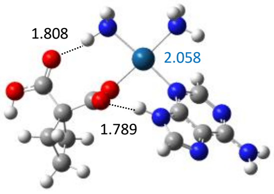

Carbo_N3-2 + 25.2

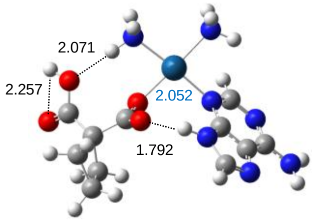

Carbo_N3-3 +19.7

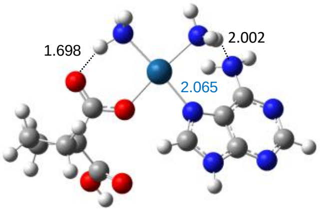

Carbo_N7-2 +27.8

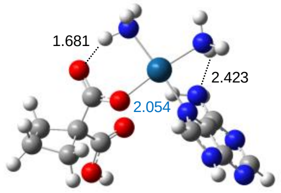

Carbo_N7-3 +37.7

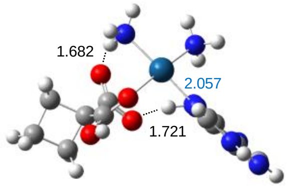

Carbo_N7-4 +126.1

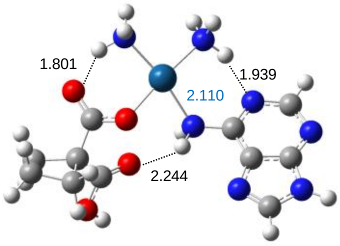

Carbo_NH2 +53.7

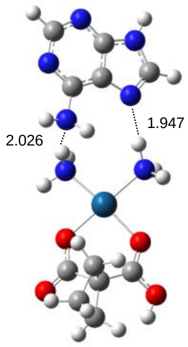

Carbo_2ndS-5 + 99.9

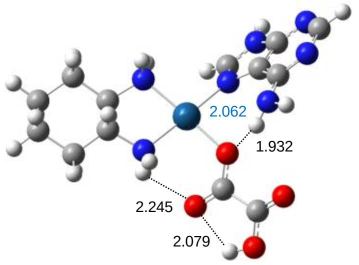

Oxal_N7-4 +18.5

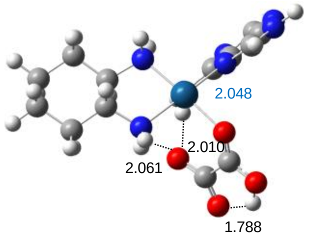

Oxal_N7-5 +53.4

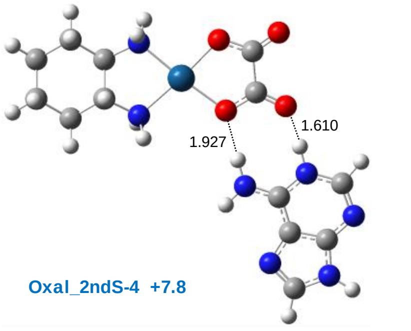

Oxal_2ndS-4 +7.8

Figure S6. Experimental IRMPD spectrum of the [CarboPt+H+A]$^+$ complex (lower panel) in both **a)** fingerprint range (irradiation time of 500 ms 5, green trace, or 180 ms,blue trace) and **a')** X−H range with a irradiation time of 1 s, compared with the computed IR spectrum of **Carbo_2ndS-3 (b-b').**

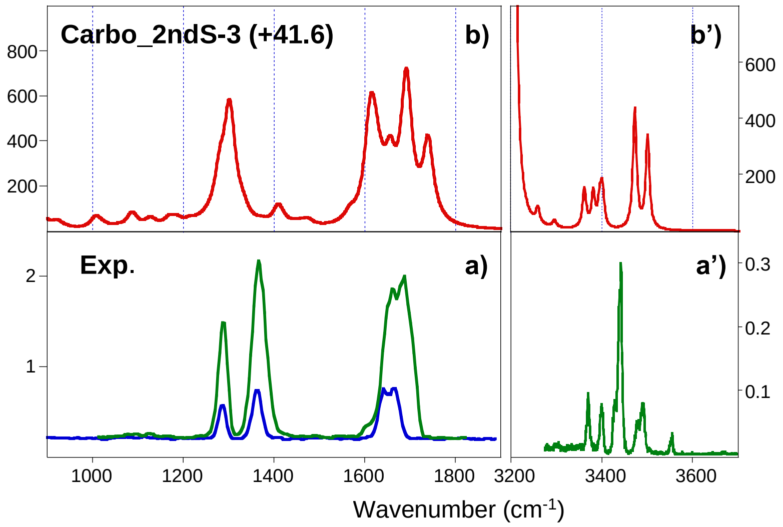

Figure S7. ModRedundant scan calculation associated with the reaction between $[CarboPt+H]^+$ ion with adenine, leading to the formation of Carbo_N3 like structures, through direct attack of adenine onto the platinum center.

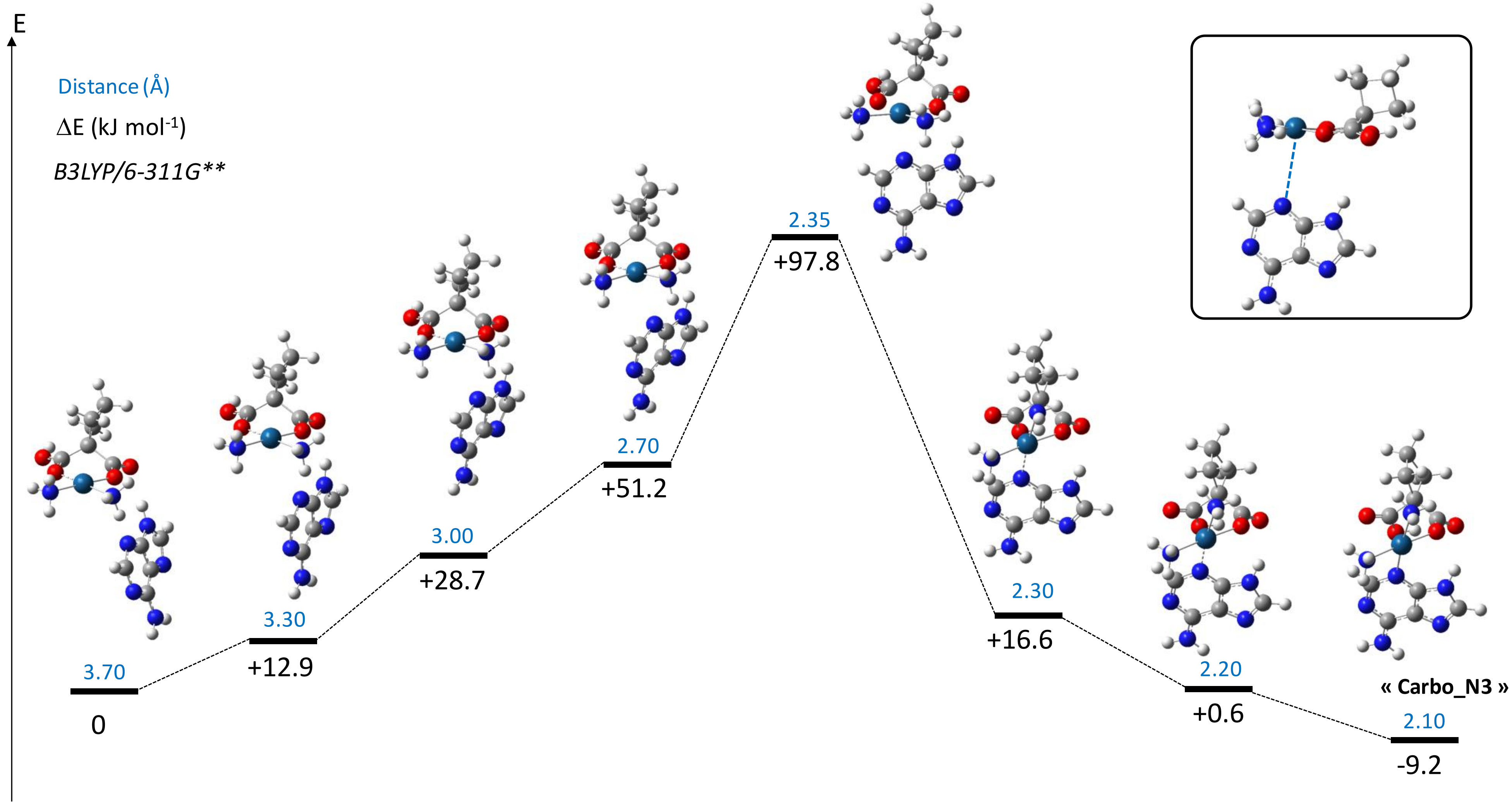

Figure S8. ModRedundant scan calculation associated with the reaction between [CarboPt+H]$^+$ ion with adenine, leading to the formation of **Carbo_N3** like structures through direct attack of adenine onto the platinum center. The initial orientation of adenine is different with respect to Figure S7.

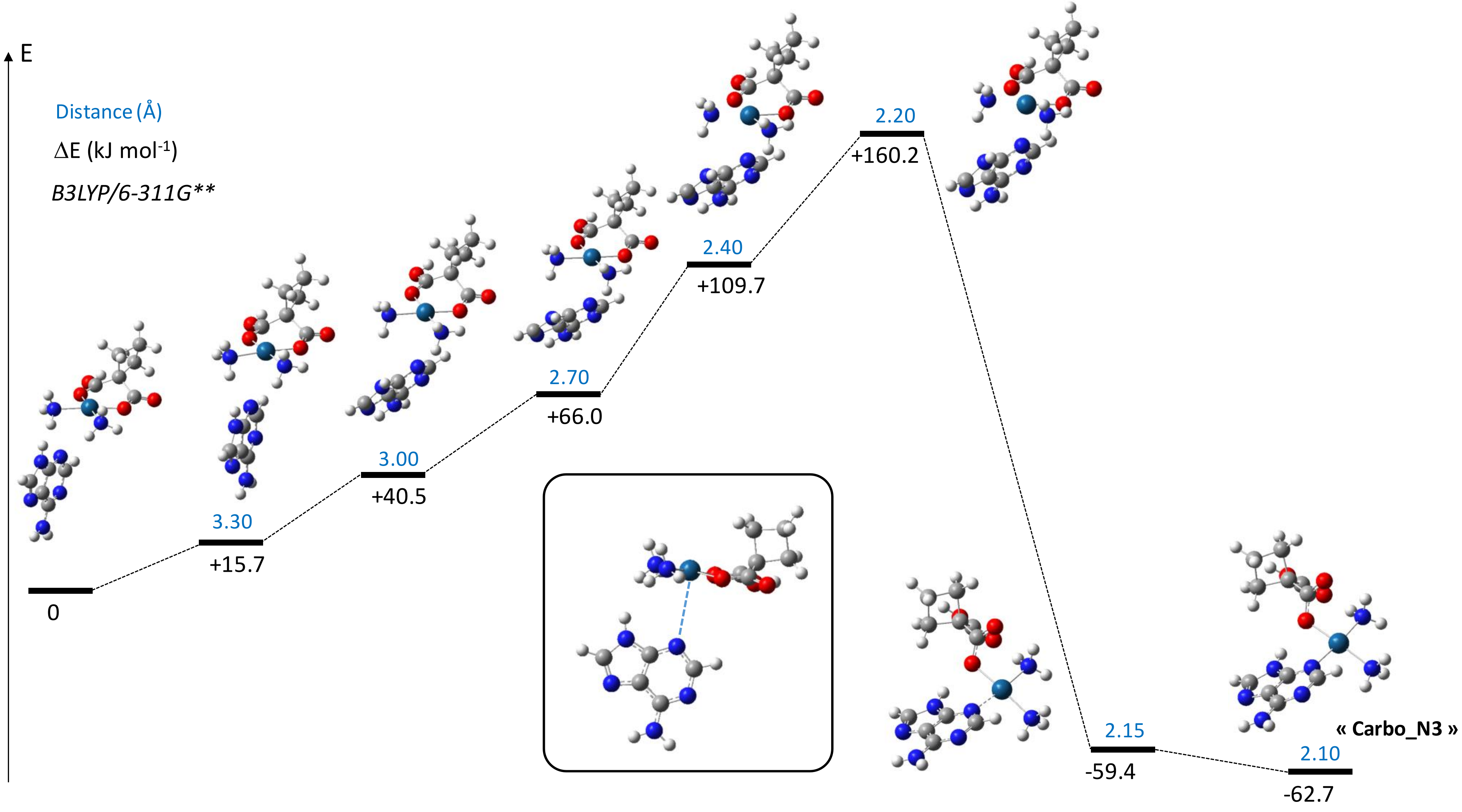

Figure S9. ModRedundant scan calculation associated with the reaction between [CarboPt+H]$^+$ ion with adenine, leading to the formation of second shell structures. This figure is the combination of two scans, the first following the proton transfer towards adenine, and the second associated with the combination of protonated adenine and carboplatin.

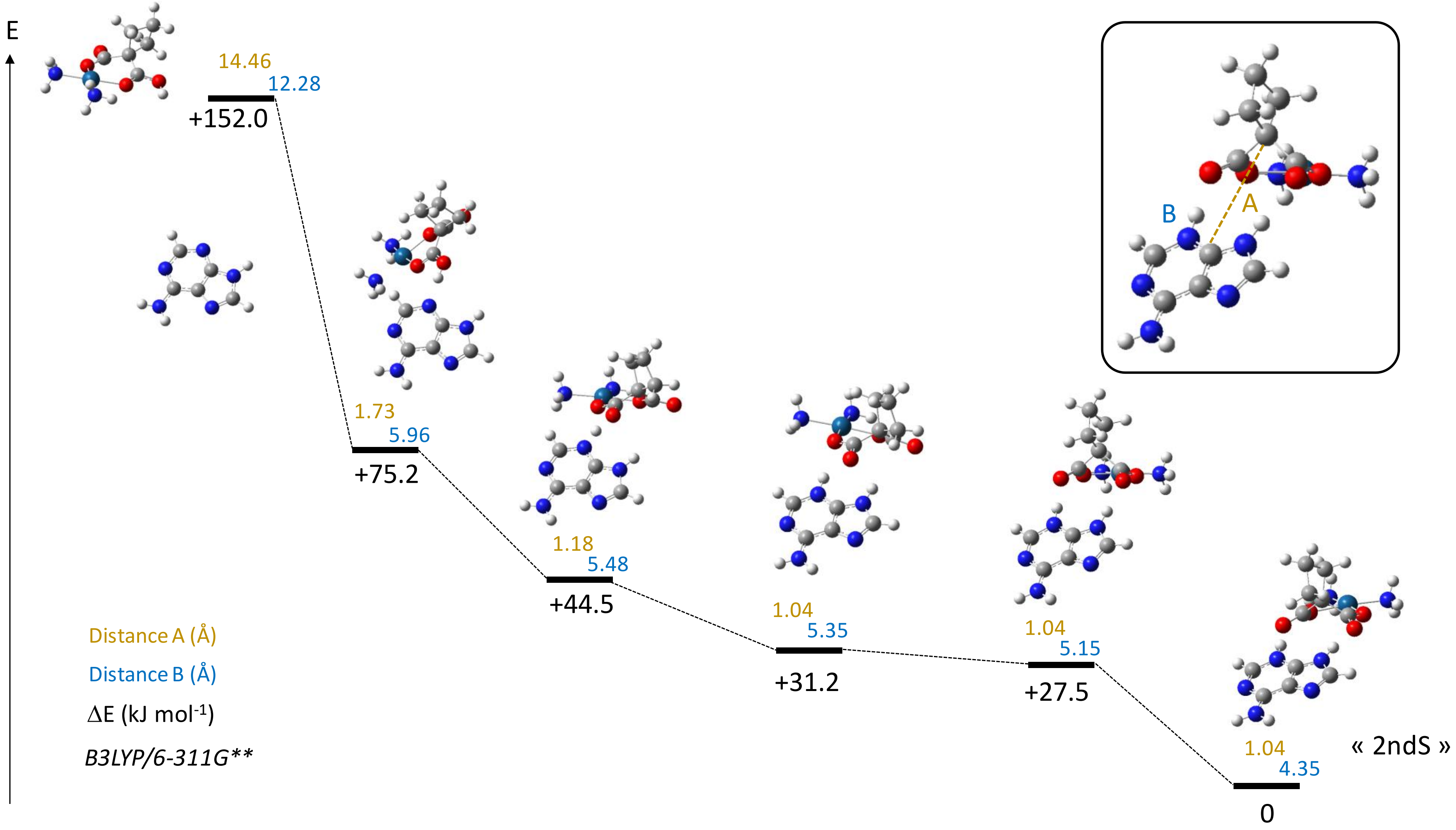

Figure S10 CID mass spectrum of [CarboPt+H+A]$^+$ ion, mass-isolated from a 7 days after mixing solution, and **(a)** mass isolated in the quadrupole of the FT-ICR-MS and recorded at CE -3.5 eV or **(b)** recorded at CE 10 eV with the LTQ XL MS.

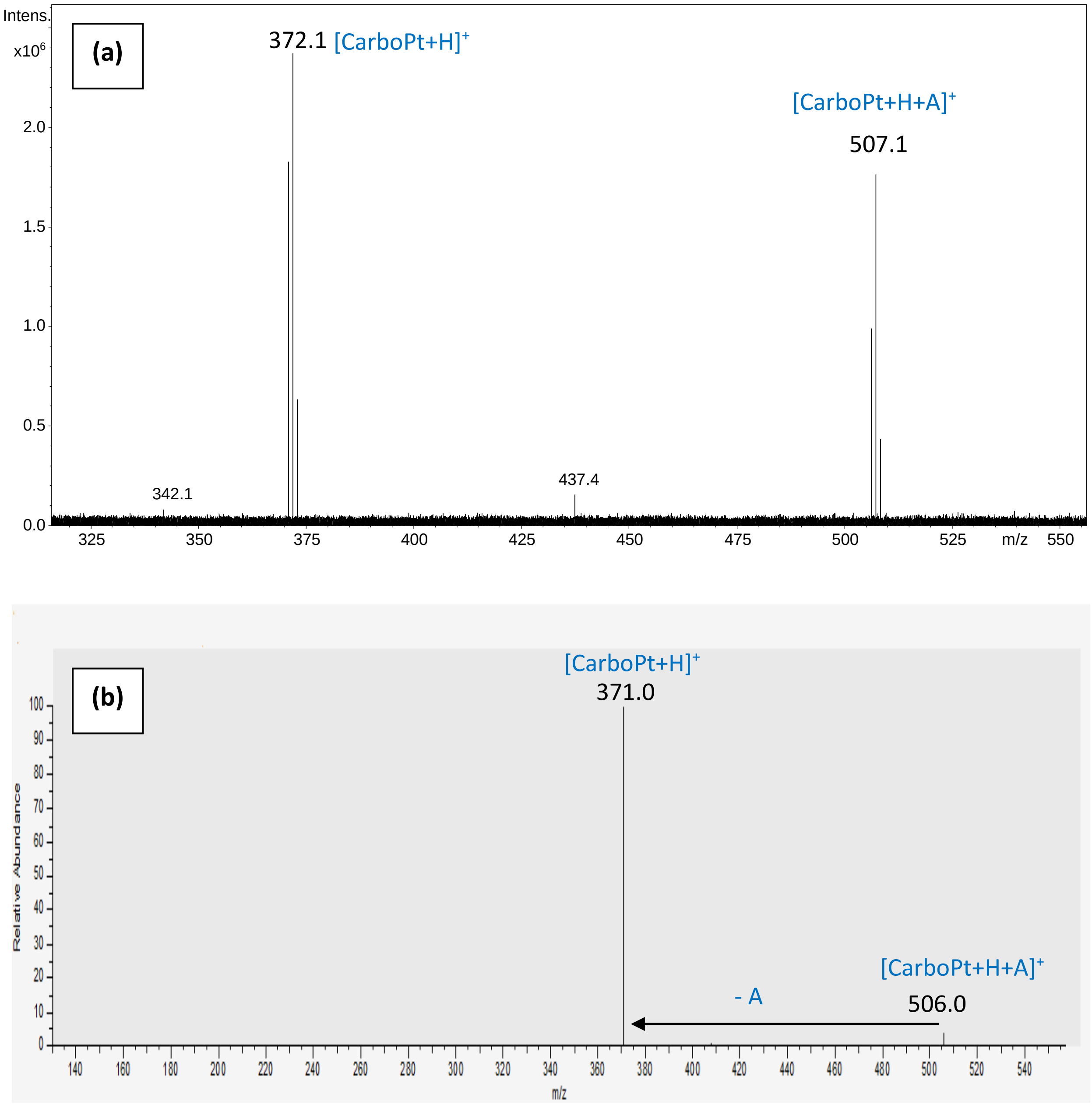

Figure S11(a). CID mass spectrum of [OxaliPt+H+A]$^+$ ion mass-isolated from a 7 days after mixing solution and (**a**) mass-isolated in the quadrupole of the FT-ICR-MS recorded at CE - 5.5 eV or (**b**) recorded at CE 10 eV with the LTQ XL MS.

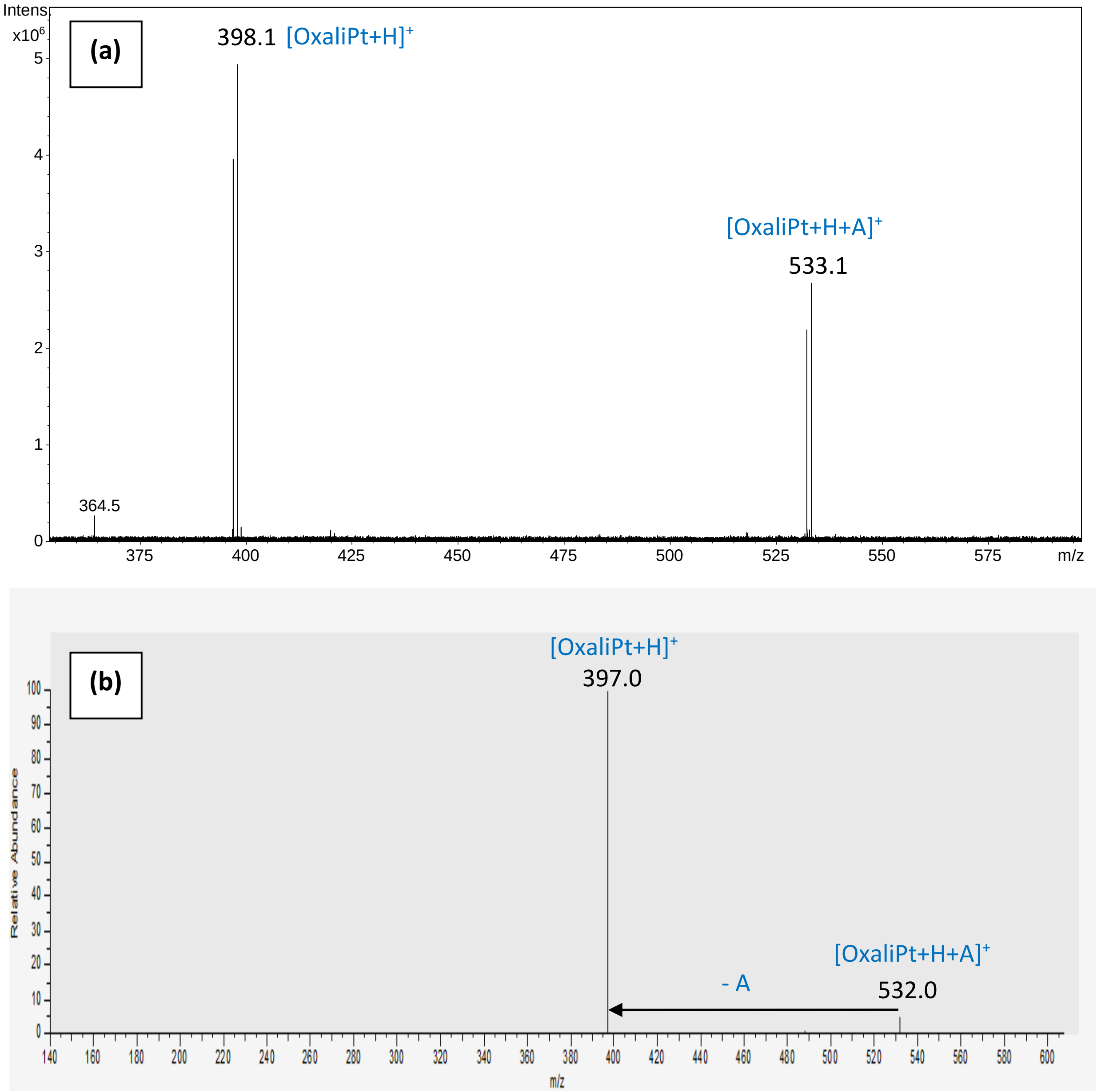

Figure S12. Most stable second shell forms computed for the $[CarboPt+H+G]^+$ and $[OxaliPt+H+G]^+$ systems. See reference 10 for more details.

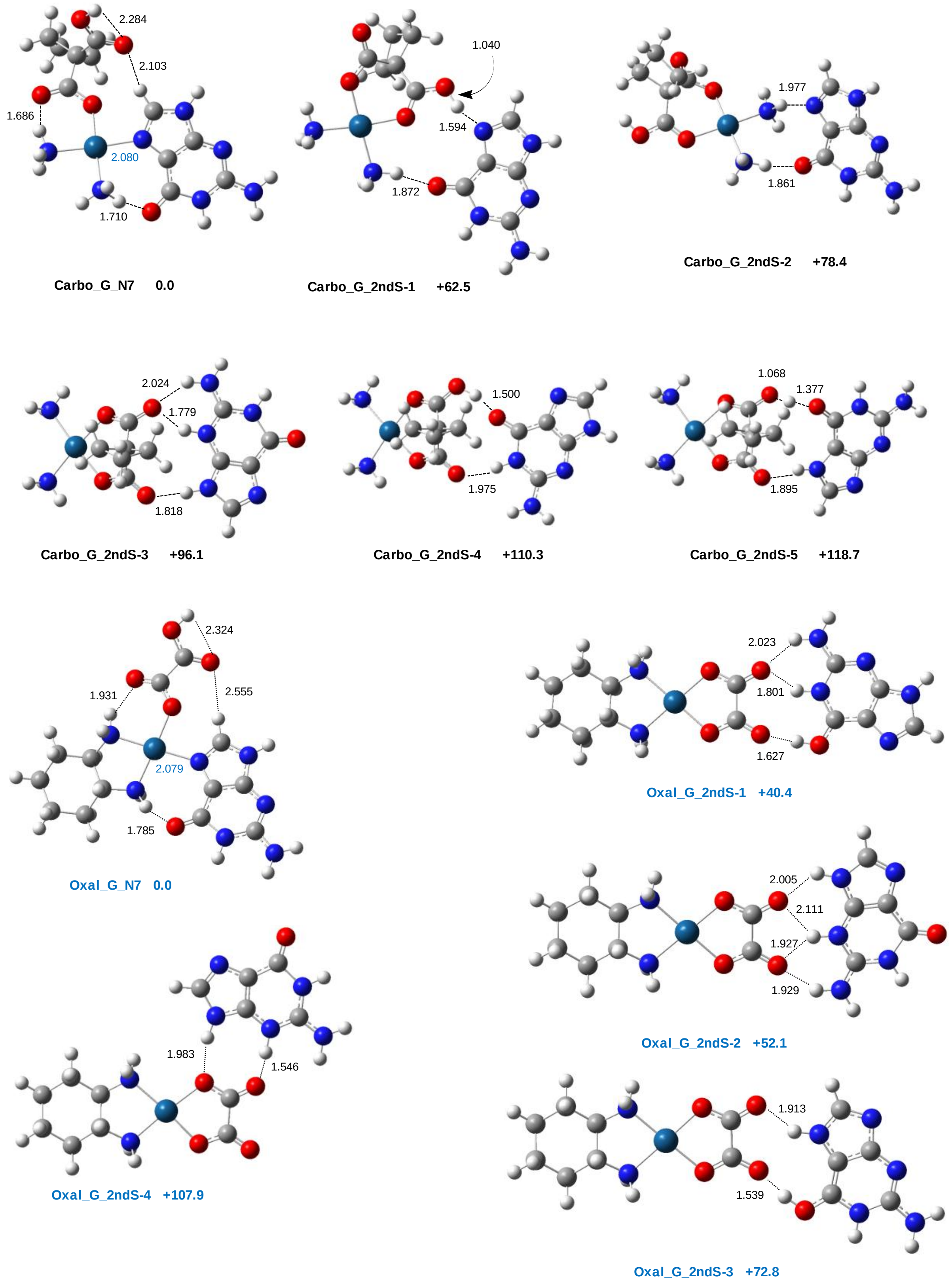

Figure S13. Experimental IRMPD spectrum of the $[CarboPt+H+G]^+$ complex (lower panel) in fingerprint range (see reference 10) compared with the computed IR spectra of the geometries reported in Figure S12.

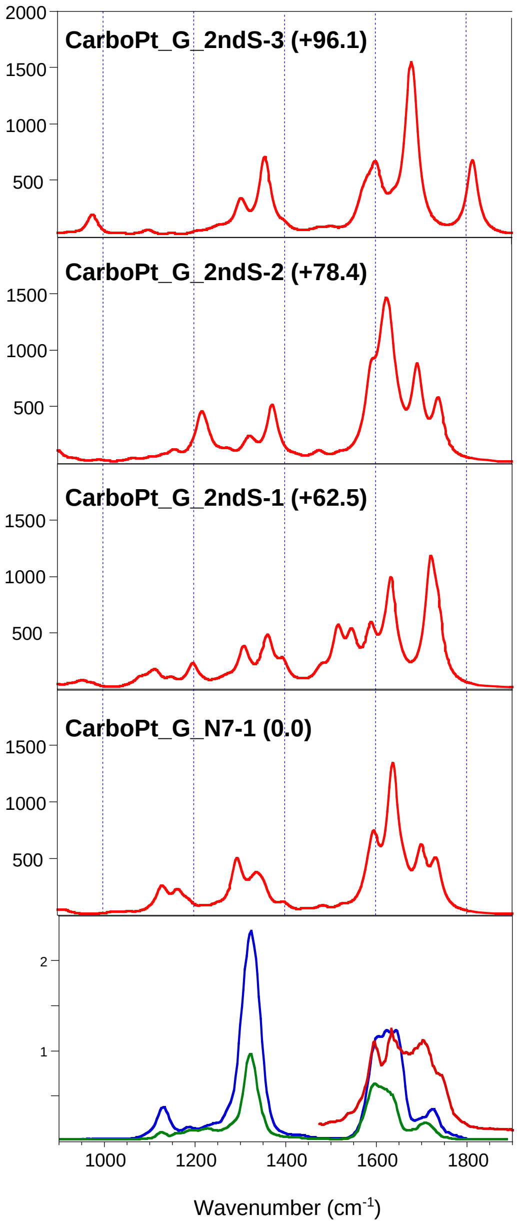

Figure S14. Experimental IRMPD spectrum of the $[OxaliPt+H+G]^+$ complex (lower panel) in the fingerprint range (see reference 10) compared with the computed IR spectra of the geometries reported in Figure S12.

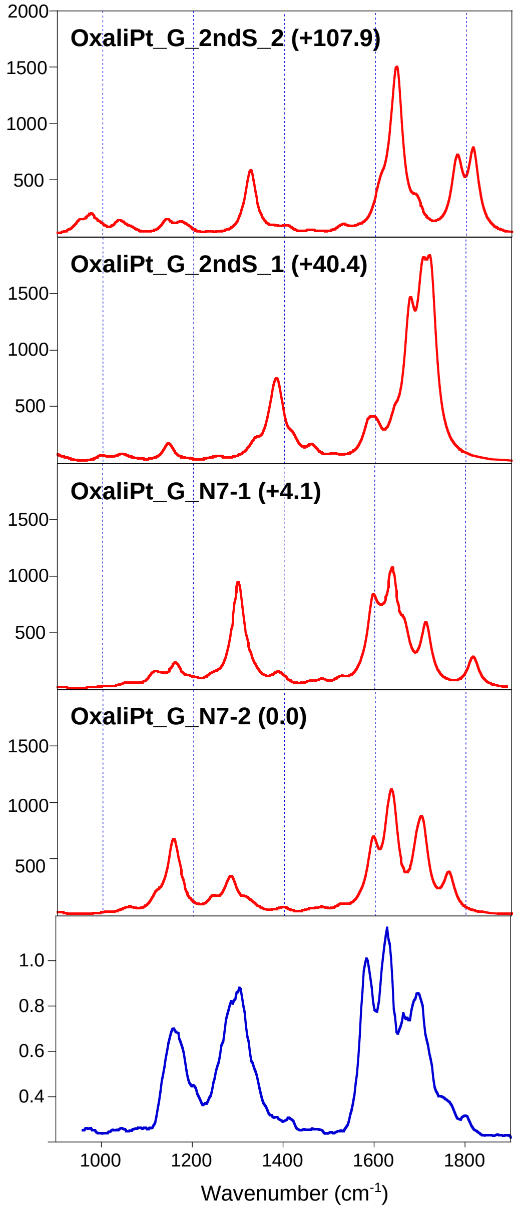

Table S1. Computational data associated with the complexes studied, obtained at the B3LYP/6-311G** level.

Structure	E+ZPE (Hartree)	ΔE (kJ mol^{-1})	H°$_{298}$ (Hartree)	ΔH°$_{298}$ (kJ mol^{-1})	G°$_{298}$ (Hartree)	ΔG°$_{298}$ (kJ mol^{-1})
[CarboPt+H+A]$^+$						
**Carbo_N1-1**	-1233.245891	+7.4	-1233.233966	+7.0	-1233.313691	+6.7
**Carbo_N1-2**	-1233.254992	+16.4	-1233.230470	+16.2	-1233.311138	+13.6
**Carbo_N1-3**	-1233.249808	+30.0	-1233.225298	+29.8	-1233.304897	+29.7
**Carbo_N1-4**	-1233.200217	+160.2	-1233.175641	+160.2	-1233.255104	+160.5
**Carbo_N3-1**	-1233.261247	0.0	-1233.236644	0.0	-1233.316226	0.0
**Carbo_N3-2**	-1233.252418	+23.2	-1233.227875	+23.0	-1233.306638	+25.2
**Carbo_N3-3**	-1233.253155	+21.2	-1233.228295	+21.9	-1233.308710	+19.7
**Carbo_N7-1**	-1233.252626	+22.6	-1233.228112	+22.4	-1233.307885	+21.9
**Carbo_N7-2**	-1233.251408	+25.8	-1233.27406	+24.3	-1233.305646	+27.8
**Carbo_N7-3**	-1233,246829	+37.9	-1233.222557	+37.0	-1233.301873	+37.7
**Carbo_N7-4**	-1233.215339	+120.5	-1233.191895	+117.5	-1233.268207	+126.1
**Carbo_NH$_2$**	-1233.239628	+56.8	-1233.215689	+55.0	-1233.295777	+53.7
**Carbo_2ndS-1**	-1233.252156	+23.9	-1233.227687	+23.5	-1233.306920	+24.4
**Carbo_2ndS-2**	-1233.251021	+29.1	-1233.225594	+29.0	-1233.305219	+28.9
**Carbo_2ndS-3**	-1233.243384	+46.9	-1233.218769	+46.9	-1233.300396	+41.6
**Carbo_2ndS-4**	-1233.238219	+60.5	-1233.213736	+60.1	-1233.293500	+59.8
**Carbo_2ndS-5**	-1233.221149	+105.3	-1233.196912	+104.3	-1233.278170	+99.9
[OxaliPt+H+A]$^+$						
**Oxal_N1-1**	-1310.664405	+8.4	-1310.640332	+7.8	-1310.718867	+8.2
**Oxal_N1-2**	-1310.658588	+23.7	-1310.634300	+23.7	-1310.713715	+21.2
**Oxal_N1-3**	-1310.645913	+56.9	-1310.621726	+56.7	-1310.700857	+53.7
**Oxal_N3-1**	-1310.667597	0.0	-1310.643320	0.0	-1310.722124	0.0
**Oxal_N3-2**	-1310.662341	+13.8	-1310.637841	+14.4	-1310.717514	+11.6
**Oxal_N3-3**	-1310.647460	+52.9	-1310.623245	+52.7	-1310.701857	+51.2
**Oxal_N7-1**	-1310.667270	+0.9	-1310.643248	+0.2	-1310.721419	+1.8
**Oxal_N7-2**	-1310.664410	+3.1	-1310.642501	+2.2	-1310.720376	+4.4
**Oxal_N7-3**	-1310.660546	+18.5	-1310.636138	+18.9	-1310.716147	+15.1
**Oxal_N7-4**	-1310.659904	+20.2	-1310.635652	+20.1	-1310.714810	+18.5
**Oxal_N7-5**	-1310.647111	+53.8	-1310.621374	+52.9	-1310.700990	+53.4
**Oxal_NH$_2$**	-1310.645825	+57.2	-1310.621856	+56.4	-1310.701513	+52.1

**Oxal_2ndS-1**	-1310.679814	–32.1	-1310.655661	–32.4	-1310.736531	–36.4
**Oxal_2ndS-2**	-1310.679315	–30.8	-1310.655102	–30.9	-1310.735462	–33.7
**Oxal_2ndS-3**	-1310.597	–10.5	-1310.647424	–10.8	-1310.727565	–13.7
**Oxal_2ndS-4**	-1310.661897	+15.0	-1310.637677	+14.8	-1310.719020	+7.8
[CarboPt+H+G]$^+$ *						
**Carbo_G_N7**	-1308.532916	0.0	-1308.507335	0.0	-1308.590291	0.0
**Carbo_G_2ndS-1**	-1308.508945	+62.9	-1308.483108	+63.6	-138.566485	+62.5
**Carbo_G_2ndS-2**	-1308.224462	+79.6	-1308.477001	+79.6	-1308.560426	+78.4
**Carbo_G_2ndS-3**	-1308.497251	+93.6	-1308.471358	+94.5	-1308.553680	+96.1
**Carbo_G_2ndS-4**	-1308.490451	+115.1	-1308.464567	+112.3	-1308.548263	+110.3
**Carbo_G_2ndS-5**	-1308.488677	+122.6	-1308.463043	+116.3	-1308.545069	+118.7
[OxaliPt+H+ G]$^+$ *						
**Oxal_G_N7**	-1385.948756	0.0	-1385.923172	0.0	-1386.004708	0.0
**Oxal_G_2ndS-1**	-1385.932271	+43.3	-1385.907088	+42.2	-1385.89328	+40.4
**Oxal_G_2ndS-2**	-1385.924520	+63.6	-1385.902010	+55.6	-1385.984858	+52.1
**Oxal_G_2ndS-3**	-1385.919422	+77.0	-1385.894041	+76.5	-1385.976986	+72.8
**Oxal_G_2ndS-4**	-1385.904745	+115.5	-1385.878897	+116.2	-1385.963598	+107.9

* For more details , go to reference 10

Table S2. Experimental spectrum of the $[OxaliPt+H+A]^+$ complex and computed vibrational modes for the lowest-energy « second shell » structures **Oxal_2ndS-1**, **Oxal_2ndS-2** and **Oxal_ 2ndS-3**

Wavenumber (cm-1)				Vibrational mode
IRMPD	Calculated[a]			
Fingerprint range (FEL)				
	Oxal_ 2ndS-1	Oxal_2ndS-2	Oxal_2ndS-3	
1140		1039 (42)		twist NH_2 (DACH) + β CH (DACH)
	1140 (124)	1140 (123)	1140 (117)	wag NH_2 (DACH) + β CH (DACH)
1172	1173 (102)			β H (A) + Rock NH2 (A) + σ N1-C2
1252		1225 (49)		β H (A) + deform Ring A
			1238 (69)	σ C-O(Pt)
1297		1275 (126)		σ N7C5 + β H (A)
			1296 (97)	def Ring A + β H (A)
1388 + 1441 (1338-1556)	1326 (41)			β CH (A) + def Ring A
		1354 (150)		β CH (DACH)
	1367 (216)	1356 (445)		σ C8-N9 + β N9H
	1371 (461)	1372 (275)	1375 (585)	σ C-C (oxa) + β N9-H
		1385 (56)		β N3-H + σ C4-C5
	1410 (61)			σ C4-N9 + σ C8-N7
		1441 (57)		Def Ring A (σ N1-C6 and σ N9-C4)
	1448 (174)			β N1-H + σ C6-NH2
			1486 (169)	def Ring A (σ C6-NH2) + β H (A)
			1582 (70)	Sciss NH2 (A) + σ C6-C5
1640 + 1720 (1600-1790)	1604 (70)	1603 (73)	1603 (70)	Sciss NH2 (DACH)
	1612 (290)		1612 (213)	Sciss NH2 (A) + def Ring A (σ C6-C5 and σ N3-C2)
		1626 (85)		σ N3-C4 + H (A)
		1645 (1240)		Sciss NH2 (A)
			1653 (404)	Sciss NH2 (A) + def Ring A (σ C6-NH2)
			1680 (884)	σ C=O (Oxalate) + β N7-H involved in the same H Bonding
		1689 (344)		β N3-H + σ C=O (Oxalate) involved in the same H Bonding
	1705 (773)			σ C=O (Oxalate) + β N1-H involved in the same H Bonding + σ C6-NH2
	1724 (1710)	1722 (1625)	1727 (1295)	σ C=O Oxalate free
X-H stretch range (OPO)				
2958	2953 (19)	2953 (20)	2953 (25)	σ CH sym (DACH)
2977	2955 (24)	2955 (23)	2955 (23)	σ CH asym (DACH)
2995-3049			2984 (2387)	σ N7-H (H bonding with C=O)
3111-3261	3234 (1855)			σ NH2 asym of A (H bonding with C=O)
			3145 (53)	σ C8H
3290	3300 (30)	3299 (86)	3299 (23)	σ NH2 sym (DACH)
		3300 (56)	3300 (16)	σ NH_2 sym (DACH)
3321		3302 (1119)		σ N9-H (H-bonded)
3345*			3358 (672)	σ NH2 asym of A (H bonding with C=O)
	3361 (32)	3360 (37)	3359 (46)	σ NH2 asym (DACH)
		3361 (33)	3361 (34)	σ NH2 asym (DACH)
3428-3442		3430 (196)		σ NH2 sym of A
3491 (3471-3512)	3473 (142)		3471 (166)	σ N9-H
	3498 (206)		3520 (218)	σ NH2 asym of A (H free)
3553		3554 (88)		σ NH2 asym of A

[a]]Vibrational frequencies calculated at the B3LYP/6-311++G(d,p) level of theory are scaled by a factor of 0.974 and 0.957 in the fingerprint and X-H stretch range respectively. The computed intensities (km mol^{-1}) are given in parenthesis. Bands with intensity lower than 40 km mol^{-1} are not reported in the fingerprint range. [b] β = bending; σ = stretching; Rock= rocking; Twist = twisting; Wag = wagging; Sciss: Scissoring, A = adenine, DACH ((1,2 diammino-cyclohexane), the numbering of the atoms follows Figure 1 of adenine.

Table S3. Experimental spectrum of the [CarboPt+H+A]$^{+}$ complex and computed vibrational modes for the lowest-energy « second shell » structures **Carbo_2ndS-1**, **Carbo_2ndS-2** and **Carbo_2ndS-4**.

Wavenumber (cm^{-1})				Vibrational mode
IRMPD	Calculated[a]			
Fingerprint range (FEL)				
	Carbo_2ndS-1	Carbo_ 2ndS-2	Carbo_ 2ndS-4	
1097	1096 (69)	1098 (58)	1099 (58)	σ C-C cyclobutane ring
1288 (1255-1305)	1289 (64)			σ N7-C5 (def ring of A)
	1300 (130)	1297 (137)	1286 (167)	NH_3 umbrella mode
	1304 (153)	1300 (152)	1301 (128)	NH_3 umbrella mode
1369 (1330-1408)		1329 (40)	1335 (54)	β C2-H
		1343 (692)	1348 (653)	σ C-O-Pt
	1345 (541)			Combined β C2-H + σ C-O-Pt
	1348 (224)			β C2-H
	1380 (56)		1375 (66)	Def ring (A)+ β H (A)
	1408 (49)			β H (A)
		1410 (61)	1410 (72)	σ N9-C4
1422	1452 (51)			σ N1-C6 + β C-H Adenine
			1460 (90)	σ C4-C5 + β C-H Adenine
1611			1583 (219)	NH2 sciss + σ C5-C6
		1590 (143)		β H (N1 and C2)+ σ C2-N3
			1606 (145)	sciss NH_2 (A) + (NH_3)
	1615 (107)		1610 (67)	sciss NH_2 of NH_3
	1617 (75)			sciss NH_2 of NH_3
1675 (1622-1725)			1627 (103)	σ C=O (H-bonded with N7H) + β N7-H
	1636 (51)	1633 (51)	1634 (48)	sciss NH_2 of NH_3
		1637 (65)		sciss NH_2 (A) + β H N1
	1637 (253)			σ N3-C4 + β N3-H
	1645 (1642)		1646 (1492)	sciss NH_2 (A) + σ C6-NH_2
	1666 (642)			Combined σ C=O(O)
		1671 (1472)		Combined σ C=O(O)
		1686 (535)		σ C6-NH_2 + β N1-H
			1690 (536)	σ C=O (H-bonded with NH2) + sciss NH_2 (A)
X-H stretch range (OPO)				
3369	3364 (32)	3364 (37)	3362 (46)	σ NH_3 (2H) asym (one NH_3)
	3365 (76)	3366 (71)	3367 (58)	σ NH_3 (2H) asym (one NH_3)
3398	3393 (9)	3396 (82)		σ NH_3 (2H) asym both NH_3
	3394 (77)			σ NH_3 (2H) sym both NH_3
			3394 (15)	σ NH_3 (2H) asym (one NH_3)
			3395 (70)	σ NH_3 (2H) sym both NH_3
3426 - 3440	3428 (209)			σ NH_2 sym of A
3477		3470(142)		σ NH_2 Of A (H free)
		3471 (149)	3467 (181)	σ N9-H
3491			3487 (173)	σ NH_2 asym of A (H free)
3554	3551 (90)			σ NH_2 asym of A

[a]Vibrational frequencies calculated at the B3LYP/6-311++G(d,p) level of theory are scaled by a factor of 0.974 and 0.957 in the fingerprint and X-H stretch range respectively. The computed intensities (km mol^{-1}) are given in parenthesis. Bands with intensity lower than 40 km mol^{-1} are not reported in the fingerprint range. [b] β = bending; σ = stretching; sciss= scissorimg; A = adenine; the numbering of the atoms follows Figure 1.

Cartesian coordinates of the various computed structures in the xyz format

[CarboPt+H+A]$^+$ complex

```
41
Carbo_N1-1
 Pt                 0.00000000    0.00000000    0.00000000
 N                  0.00000000    0.00000000    2.10979550
 N                  2.08972849    0.00000000   -0.18324569
 H                  2.54825487    0.78795506    0.27078578
 H                  2.31104584    0.06669097   -1.19336019
 H                  2.52781700   -0.85299321    0.16018446
 H                  0.53442499   -0.76501941    2.51956152
 H                 -0.95361264   -0.08382522    2.46120285
 H                  0.37267888    0.87154433    2.48521375
 O                  0.04803667   -0.05919170   -2.02634550
 C                  0.19495945    1.06664641   -2.67369622
 O                 -0.01451910    2.18216715   -2.21065353
 C                  2.14259737    0.37009597   -4.00703487
 O                  2.79288273    0.24020829   -2.98515174
 O                  2.67056090    0.11147734   -5.20786108
 C                  0.71058650    0.85247583   -4.09854231
 C                 -0.27427605    0.01669610   -4.99502780
 C                  0.44342968    2.06985445   -5.04580953
 C                 -0.80414021    1.33916656   -5.60529073
 H                 -0.96943052   -0.62273533   -4.45265019
 H                  0.27645632   -0.58194092   -5.71960271
 H                  0.29439531    3.01546728   -4.52851897
 H                  1.23220892    2.17156839   -5.79020134
 H                 -1.72619610    1.67689138   -5.12836596
 H                 -0.94331819    1.35876246   -6.68562619
 H                 -6.74616782   -0.84336753    0.47364334
 N                 -6.06248328   -0.11919803    0.30533758
 C                 -6.30321671    1.21682451    0.05402885
 H                 -7.30554544    1.61742825    0.03200465
 N                 -5.20887840    1.90667587   -0.14650758
 C                 -4.20079794    0.98017340   -0.01725093
 C                 -2.80458918    1.12259009   -0.15338990
 N                 -2.06566395   -0.01320633    0.05003086
 C                 -2.69817260   -1.20701584    0.29491604
 N                 -3.98610823   -1.41135863    0.41590620
 C                 -4.70570637   -0.28744658    0.26053256
 N                 -2.22764771    2.29283714   -0.43639308
 H                 -2.86173719    3.05489616   -0.63600213
 H                 -1.30644613    2.32080254   -0.89157143
 H                 -2.03816163   -2.06051718    0.39174915
 H                  3.59027501   -0.16634393   -5.07595733
```

```
41
Carbo_N1-2
 Pt                 0.00000000    0.00000000    0.00000000
 N                  0.00000000    0.00000000    2.10481469
 N                  2.09017768    0.00000000   -0.04803230
 H                  2.52826371    0.77187641    0.45026212
 H                  2.30052928    0.10267911   -1.07150952
 H                  2.50686571   -0.86871585    0.28080734
 H                  0.47563008    0.81970842    2.48077420
 H                  0.44270961   -0.82382118    2.51112608
 H                 -0.95497615    0.03146224    2.46139571
 O                 -0.19604585    0.08346509   -2.02424303
 C                  0.74670258    0.27827266   -2.90518087
 O                  1.95468452    0.27683121   -2.68334496
 C                 -0.45292711    1.89298118   -4.34435846
 O                 -0.81071448    2.25181345   -5.58708277
 O                 -0.65517205    2.60917042   -3.38587756
 C                  0.20040525    0.52582448   -4.31513981
 C                  1.23568465    0.19614853   -5.43970971
 C                 -0.67310240   -0.66927741   -4.85522986
 C                  0.55921154   -1.18395287   -5.64052670
 H                  2.27427397    0.21271915   -5.11640191
 H                  1.10314347    0.85197401   -6.29850619
 H                 -1.10805250   -1.31176139   -4.09075092
 H                 -1.46158593   -0.30570569   -5.51411336
 H                  1.08371266   -1.98187180   -5.11142634
 H                  0.38563847   -1.50296017   -6.66772620
 H                 -6.75155421   -0.81804048    0.56754616
 N                 -6.07444205   -0.14857149    0.23062547
 C                 -6.32842317    1.07510348   -0.35602289
 H                 -7.33547891    1.43953876   -0.49276716
 N                 -5.23995738    1.70982676   -0.71121608
 C                 -4.22214391    0.86488107   -0.33586097
 C                 -2.82582582    0.99489754   -0.48470633
 N                 -2.07320082   -0.03517418    0.01272000
 C                 -2.69500675   -1.13956305    0.53889055
 N                 -3.98125055   -1.33294056    0.69187135
 C                 -4.71497068   -0.29842951    0.24867929
 N                 -2.27450202    2.07372749   -1.04449610
 H                 -2.92186509    2.74711073   -1.43120534
 H                 -2.02738383   -1.93389112    0.85146283
 H                 -1.37012719    2.02529104   -1.50586380
 H                 -1.21477278    3.13145715   -5.53361519
```

41
Carbo_N1-3

Pt	0.00000000	0.00000000	0.00000000
N	0.00000000	0.00000000	2.10736333
N	2.08879617	0.00000000	-0.16958137
H	2.55657684	0.74777876	0.33942636
H	2.31946532	0.13149617	-1.17207264
H	2.51324037	-0.88080734	0.11661976
H	0.54652499	-0.75721809	2.51580529
H	-0.95148758	-0.09754380	2.46094649
H	0.36116261	0.87691425	2.48177231
O	0.05273435	-0.05872884	-2.02893398
C	0.18452050	1.06492653	-2.67867705
O	-0.04757090	2.18111548	-2.22959597
C	2.20699493	0.50607726	-3.96513891
O	2.79586511	0.40271160	-2.91120423
O	2.88945182	0.31187987	-5.10150374
C	0.72775133	0.85811604	-4.09793643
C	-0.17351670	-0.09038492	-4.96807126
C	0.34000880	2.02260227	-5.06729522
C	-0.83528757	1.16186583	-5.59958084
H	-0.79608225	-0.78594577	-4.40827721
H	0.41278850	-0.66346104	-5.69067926
H	0.10815544	2.96215241	-4.57027438
H	1.10290719	2.20901051	-5.82727049
H	-1.77863708	1.41700843	-5.11402485
H	-0.99438438	1.14767360	-6.67748834
H	-6.74401193	-0.85660463	0.49668200
N	-6.06232678	-0.13042486	0.32875104
C	-6.30630064	1.20738076	0.09038882
H	-7.30921891	1.60697002	0.07881892
N	-5.21418018	1.90020427	-0.11215189
C	-4.20412730	0.97390787	0.00273224
C	-2.80905327	1.11889860	-0.14081203
N	-2.06725748	-0.01724693	0.04835862
C	-2.69671711	-1.21386556	0.28719967
N	-3.98374200	-1.42088039	0.41440612
C	-4.70569908	-0.29667317	0.27351060
N	-2.23456899	2.29222446	-0.41753858
H	-2.87028472	3.05587942	-0.60543622
H	-1.31852816	2.32270205	-0.88185060
H	-2.03506440	-2.06726312	0.37341451
H	2.32918082	0.43635713	-5.87764221

41
Carbo_N1-4

Pt	0.00000000	0.00000000	0.00000000
N	0.00000000	0.00000000	2.07022954
N	2.09086728	0.00000000	-0.22563030
H	2.62695509	0.39266386	0.54571235
H	2.29051785	0.56995617	-1.05247912
H	2.44591660	-0.93900462	-0.40292178
H	-0.58149840	0.80465483	2.33886909
H	0.90845859	0.10280302	2.51754371
H	-0.42991489	-0.84470542	2.44544312
O	-0.17924464	0.14382182	-2.08219968
C	0.10414158	1.23562133	-2.63719358
O	1.38010986	1.61796933	-2.59758623
C	-1.50371427	2.85131912	-1.88265052
O	-2.64990966	3.24938530	-1.96010508
O	-0.67363580	2.89093719	-0.92235178
C	-0.91961472	2.10814363	-3.21496795
C	-0.53846558	3.05721910	-4.39546254
C	-1.98129387	1.43583094	-4.12904390
C	-1.36438804	2.15877860	-5.35542607
H	0.52227950	3.18229038	-4.63962112
H	-0.97686978	4.04488729	-4.25354352
H	-1.99289767	0.34601828	-4.13962640
H	-2.96604814	1.81630245	-3.85962718
H	-0.73266809	1.50329208	-5.95784799
H	-2.06025652	2.67742308	-6.01399385
H	-6.72568020	-0.95168066	0.29091561
N	-6.01808770	-0.29165737	0.58101683
C	-6.20752171	0.88774686	1.27688521
H	-7.18879952	1.20122442	1.60051307
N	-5.09810324	1.54797354	1.48443069
C	-4.12934133	0.76619948	0.89997025
C	-2.75083629	0.97393631	0.75391361
N	-2.05736989	-0.02301316	0.12336607
C	-2.72017648	-1.10055884	-0.40545994
N	-4.00796362	-1.33434143	-0.33838857
C	-4.67878827	-0.38543019	0.32939781
N	-2.09187488	2.06067688	1.23289426
H	-2.73527908	2.74100631	1.62606639
H	-2.09581345	-1.81644632	-0.92448913
H	-1.44454678	2.50221698	0.52515702
H	1.47022987	2.50634033	-2.97568289

41

Carbo_N3-1

Pt	0.00000000	0.00000000	0.00000000
N	0.00000000	0.00000000	2.10689589
N	2.09079027	0.00000000	-0.16817309
H	2.54237278	0.80668707	0.25932796
H	2.32240976	0.02921378	-1.17805928
H	2.52988213	-0.83702803	0.21147481
H	0.43196595	-0.83119654	2.50951486
H	-0.95625157	0.03797002	2.45994378
H	0.48246077	0.81230354	2.48967632
O	0.06258107	-0.07799007	-2.02833027
C	0.22824508	1.01115049	-2.72668897
O	0.00056787	2.15238681	-2.33405684
C	2.19523198	0.22033869	-3.98944045
O	2.82784406	0.12849312	-2.95239283
O	2.73638411	-0.10438801	-5.16764451
C	0.77458944	0.72376378	-4.12777898
C	-0.20930113	-0.13548200	-5.00475211
C	0.54918989	1.89926355	-5.13717251
C	-0.69989806	1.16551108	-5.68906862
H	-0.92820891	-0.73485818	-4.44781880
H	0.34438409	-0.77891308	-5.68732118
H	0.40779418	2.87114865	-4.66881150
H	1.35496873	1.95135989	-5.86825486
H	-1.62620691	1.54188592	-5.25085692
H	-0.81473898	1.13547183	-6.77195481
H	-1.46769259	2.41191432	-1.35703412
N	-2.41472420	2.22154881	-0.99220711
C	-3.58155733	2.91404828	-1.25476734
H	-3.56184475	3.87853442	-1.73929033
N	-4.66117076	2.28792991	-0.86072175
C	-4.19234889	1.10979000	-0.32691562
C	-4.85653757	-0.00106380	0.23262859
N	-4.11177112	-1.05346605	0.64913976
C	-2.80118479	-1.00763140	0.53752987
N	-2.05624964	0.01302848	0.04517342
C	-2.80023837	1.05723921	-0.40642840
N	-6.18360813	-0.06024802	0.36379892
H	-6.75901443	0.70176445	0.04116068
H	-6.60969395	-0.89122176	0.74277178
H	-2.24711724	-1.87962846	0.86684095
H	3.64899870	-0.39156440	-5.00885449

41
Carbo_N3-2

Pt	0.00000000	0.00000000	0.00000000
N	0.00000000	0.00000000	2.10417718
N	2.08978741	0.00000000	-0.15407581
H	2.55266585	0.75609360	0.34710272
H	2.33053600	0.11557023	-1.15643173
H	2.51350050	-0.87546093	0.14945570
H	0.44644301	-0.82399145	2.50594251
H	-0.95559959	0.02335098	2.46012956
H	0.47102886	0.82026261	2.48450116
O	0.06933562	-0.07473401	-2.03079030
C	0.21771304	1.01139499	-2.73341836
O	-0.03179114	2.15283547	-2.35728547
C	2.26785983	0.37862944	-3.94884527
O	2.83616138	0.32682193	-2.87976123
O	2.97129415	0.12372943	-5.05946863
C	0.79356068	0.72937109	-4.12783867
C	-0.09334915	-0.26270075	-4.96513540
C	0.43230811	1.84034271	-5.16787274
C	-0.73129553	0.95423098	-5.68403477
H	-0.73431867	-0.92101673	-4.38172905
H	0.50640603	-0.88041612	-5.63801248
H	0.19095238	2.80543698	-4.72767371
H	1.21467709	1.98617728	-5.91683939
H	-1.68831587	1.23770265	-5.24294270
H	-0.85855218	0.87969498	-6.76363884
H	-1.47305328	2.42692094	-1.33366199
N	-2.41465281	2.24023923	-0.95451323
C	-3.57994985	2.94498945	-1.19053303
H	-3.55984768	3.91724952	-1.65931270
N	-4.65865715	2.32001914	-0.79240537
C	-4.19132977	1.13009990	-0.28376819
C	-4.85593340	0.01484923	0.26668812
N	-4.11312373	-1.04976385	0.65465543
C	-2.80359917	-1.01051881	0.52798986
N	-2.05791300	0.01374940	0.04427362
C	-2.80082944	1.06919495	-0.38212757
N	-6.18135932	-0.03695387	0.41568983
H	-6.75546824	0.73594370	0.11756314
H	-6.60809394	-0.86998354	0.78942531
H	-2.25136219	-1.89134036	0.83628713
H	2.42756187	0.20833325	-5.85257698

41

Carbo_N3-3

Pt	0.00000000	0.00000000	0.00000000
N	0.00000000	0.00000000	2.10917660
N	2.09399123	0.00000000	-0.20523378
H	2.53330737	0.86663776	0.10175044
H	2.28342574	-0.09087107	-1.21289576
H	2.56547276	-0.77188403	0.26279610
H	0.42684579	-0.83264437	2.51433149
H	-0.95741792	0.04133149	2.45904553
H	0.48362600	0.81084095	2.49361342
O	0.06286892	-0.07913514	-2.02646599
C	0.20649703	1.01609566	-2.72869410
O	0.00096971	2.15370897	-2.31376868
C	1.77451215	-0.27165344	-4.22252044
O	2.72006470	-0.06592964	-3.23744502
O	1.93453493	-1.10874185	-5.06289641
C	0.63816461	0.72431555	-4.16032573
C	-0.58372034	0.40137993	-5.08464544
C	0.87564239	2.01818375	-5.01635512
C	-0.53895030	1.85184049	-5.62828770
H	-1.49159613	0.06654771	-4.58216621
H	-0.29235018	-0.34202308	-5.82605954
H	1.07209118	2.91617666	-4.43416858
H	1.66925311	1.86869788	-5.74956337
H	-1.26950287	2.50320954	-5.14675055
H	-0.62180500	1.96912527	-6.70820285
H	-1.47173931	2.42487194	-1.32985360
N	-2.41668591	2.22912760	-0.96514023
C	-3.58623052	2.92020733	-1.22168936
H	-3.56996687	3.88858018	-1.69850834
N	-4.66299734	2.28705360	-0.83212961
C	-4.19027996	1.10619527	-0.30820885
C	-4.85142892	-0.01209539	0.24054845
N	-4.10301699	-1.06582516	0.64833034
C	-2.79328456	-1.01575919	0.53777798
N	-2.05144581	0.01251747	0.05461462
C	-2.79817340	1.05888588	-0.38855249
N	-6.17777866	-0.07750611	0.36966884
H	-6.75612075	0.68442296	0.05175951
H	-6.60135550	-0.91441628	0.73858632
H	-2.23636637	-1.88895349	0.85890520
H	3.46041341	-0.65130323	-3.46406269

41
Carbo_N7-1

Pt	0.00000000	0.00000000	0.00000000
N	0.00000000	0.00000000	2.11313937
N	2.08210576	0.00000000	-0.20073875
H	2.55407007	0.75069851	0.30053689
H	2.29473316	0.13166180	-1.20667639
H	2.51167961	-0.87979120	0.08077413
H	0.86919376	-0.30362681	2.54810686
H	-0.73855776	-0.59601171	2.48475210
H	-0.18836275	0.94959834	2.43747587
O	0.01837476	0.02225536	-2.02402607
C	0.15944045	1.19996254	-2.58131935
O	-0.00709666	2.27344512	-2.01846321
C	2.07325255	0.62378779	-4.00221934
O	2.74149164	0.42390646	-3.00316478
O	2.58248041	0.46180409	-5.22746727
C	0.63439863	1.09579284	-4.03210191
C	-0.35959997	0.31814803	-4.96800319
C	0.33658331	2.37785109	-4.87930205
C	-0.91251719	1.67589422	-5.47146556
H	-1.03965537	-0.36410469	-4.45961447
H	0.18068852	-0.22274768	-5.74407803
H	0.18564946	3.27902983	-4.28838352
H	1.11071532	2.54720566	-5.62678193
H	-1.83033891	1.96798327	-4.95776171
H	-1.06850251	1.77257691	-6.54530714
H	-4.92991671	-1.33466803	-0.06048192
N	-4.13806366	-0.71122615	0.01491667
C	-2.83313807	-1.04441322	-0.12667926
H	-2.48954467	-2.04207857	-0.34459657
N	-2.04815563	0.01017298	0.03671043
C	-2.89018468	1.09721976	0.27660317
C	-2.70144537	2.48677937	0.47553757
N	-3.78451384	3.21477136	0.77772068
C	-4.99001429	2.63435155	0.80771206
N	-5.29547888	1.36023827	0.54614048
C	-4.20969490	0.64248112	0.29235501
N	-1.49067376	3.09719864	0.40261351
H	-0.84026587	2.75446336	-0.30868476
H	-1.55284750	4.10586479	0.46563930
H	-5.82050870	3.28472430	1.06006780
H	3.50711881	0.18496492	-5.13294122

41
Carbo_N7-2

Pt	0.00000000	0.00000000	0.00000000
N	0.00000000	0.00000000	2.10099123
N	2.08265095	0.00000000	-0.05210626
H	2.52201362	0.75061066	0.47719969
H	2.29723758	0.14429019	-1.06924954
H	2.49699602	-0.88294247	0.24033485
H	0.92203858	-0.12527297	2.51458004
H	-0.60541648	-0.75742024	2.45679059
H	-0.36344650	0.88443781	2.45458065
O	-0.17390369	0.11688420	-2.02248519
C	0.74917370	0.39776712	-2.90222786
O	1.95946589	0.41027938	-2.69473435
C	-1.26539675	0.36795510	-4.38221362
O	-2.21217065	1.01251769	-3.98822398
O	-1.40075282	-0.82385860	-4.98762225
C	0.18327693	0.79158263	-4.26749501
C	0.44567415	2.32285882	-4.52993563
C	1.15039048	0.46911757	-5.45073489
C	1.63799432	1.94071568	-5.44342827
H	0.65873949	2.93362432	-3.65220849
H	-0.39308605	2.76559650	-5.06750531
H	1.89449279	-0.29943718	-5.24969491
H	0.59455625	0.20388123	-6.34976793
H	2.59699086	2.05173364	-4.93926416
H	1.68211427	2.44369485	-6.40928417
H	-4.75003446	1.18173609	-1.33609311
N	-4.05153889	0.70305356	-0.78345854
C	-2.71507864	0.78189951	-0.96239957
H	-2.25495722	1.35241619	-1.75414086
N	-2.06295953	0.01924881	-0.09246466
C	-3.04013173	-0.61746735	0.67639985
C	-3.05655670	-1.61130531	1.66952528
N	-4.21774113	-1.94731076	2.22163840
C	-5.34934460	-1.37913688	1.77509621
N	-5.47088070	-0.50802930	0.77420765
C	-4.29944208	-0.16510810	0.26047280
N	-1.90370719	-2.24598127	2.13211186
H	-2.14321662	-2.98911012	2.78151061
H	-1.30099525	-2.58471685	1.38794748
H	-6.26347733	-1.68370581	2.27154084
H	-2.34867813	-1.02087572	-5.03929312

41
Carbo_N7-3

Pt	0.00000000	0.00000000	0.00000000
N	0.00000000	0.00000000	2.10267275
N	2.07681331	0.00000000	-0.14141826
H	2.46521146	0.94137446	-0.15104032
H	2.24338027	-0.44202369	-1.07788012
H	2.57435129	-0.54029142	0.56295923
H	0.90937448	0.13682639	2.53954510
H	-0.35440979	-0.91845248	2.39143709
H	-0.62131129	0.71026068	2.48737796
O	-0.20733700	-0.18924509	-2.01240220
C	0.55089790	-1.00860715	-2.69751090
O	1.75153171	-1.18998000	-2.52020346
C	-1.08976489	-2.79071318	-3.01756454
O	-1.89104763	-3.47464865	-3.85076778
O	-1.07008422	-3.00427611	-1.82265406
C	-0.23031071	-1.78181525	-3.75809889
C	-0.93513653	-0.86408237	-4.81959293
C	0.65903637	-2.34391882	-4.91700064
C	0.22424824	-1.15180372	-5.80766967
H	-1.10119116	0.16608383	-4.50622838
H	-1.87823250	-1.30035935	-5.14686467
H	1.71419882	-2.43741971	-4.66784767
H	0.27765577	-3.29676550	-5.28254315
H	0.96829808	-0.35338551	-5.82053525
H	-0.05628251	-1.38297456	-6.83471877
H	-4.90245258	1.30813520	-0.57210721
N	-4.12843189	0.72551810	-0.28376017
C	-2.81253482	0.97559303	-0.48891505
H	-2.44232574	1.84475941	-1.00712907
N	-2.05354758	0.02070549	0.02418836
C	-2.92340689	-0.91771645	0.57303822
C	-2.76877446	-2.16897329	1.20115917
N	-3.85828041	-2.76549021	1.68306658
C	-5.05962990	-2.19385151	1.50111263
N	-5.33405575	-1.06319392	0.84863261
C	-4.23449528	-0.46675723	0.40843225
N	-1.53895831	-2.76866654	1.36844160
H	-1.63349330	-3.70542233	1.74635869
H	-0.96180389	-2.74991053	0.52964287
H	-5.90528807	-2.72660960	1.92138446
H	-2.37370459	-4.12984566	-3.32425753

41
Carbo_N7-4

Pt	0.00000000	0.00000000	0.00000000
N	0.00000000	0.00000000	2.06003874
N	2.07849848	0.00000000	-0.10017582
H	2.57616100	0.35969130	0.71106133
H	2.41670516	0.52495647	-0.90449509
H	2.30598654	-1.02216791	-0.23271139
H	0.91720709	0.10063927	2.49020269
H	-0.38103819	-0.92912116	2.33023025
H	-0.59812182	0.72634069	2.45131074
O	-0.08328081	-0.09521853	-2.09200198
C	0.12988712	-1.05019906	-2.87863247
O	-0.21814165	-0.86249975	-4.13705710
C	0.78910483	-2.68496106	-1.08958756
O	-0.35443097	-2.95551912	-0.65178606
O	1.86873924	-2.63128645	-0.45582503
C	0.81708519	-2.34365101	-2.60454977
C	0.38756743	-3.51946468	-3.55573374
C	2.22834542	-2.36099681	-3.32769180
C	1.67526774	-3.33333449	-4.39719943
H	-0.56294167	-3.39239305	-4.07086303
H	0.35798265	-4.45205075	-2.99392062
H	2.61584136	-1.40414498	-3.68361372
H	2.94685974	-2.80431400	-2.63992138
H	1.48598072	-2.84830720	-5.35468998
H	2.26872647	-4.23012889	-4.57182450
H	-4.97011316	1.01573761	-0.60466135
N	-4.16364173	0.50348052	-0.27463448
C	-2.86735250	0.77624055	-0.54704429
H	-2.54677097	1.59514212	-1.16949366
N	-2.05510652	-0.07882371	0.05611708
C	-2.87162132	-0.96910484	0.76071106
C	-2.65480544	-2.09109278	1.59595832
N	-3.72096926	-2.63180170	2.19094650
C	-4.94513033	-2.14856569	1.93822773
N	-5.27280251	-1.14332034	1.12364335
C	-4.20272564	-0.58916325	0.57317990
N	-1.40327072	-2.59668199	1.85614029
H	-1.46809735	-3.41829591	2.44928257
H	-0.84336047	-2.78512891	0.98975200
H	-5.76401332	-2.64092083	2.45096010
H	-0.58522181	0.03015344	-4.24132735

41

Carbo_NH2

Pt	0.00000000	0.00000000	0.00000000
N	0.00000000	0.00000000	2.09458447
N	2.07484035	0.00000000	-0.14580969
H	2.59102675	-0.41633265	0.62539668
H	2.45208692	0.93130336	-0.31237851
H	2.21898004	-0.57325203	-1.00417205
H	-0.98951877	-0.05375239	2.39129159
H	0.40719286	0.84142070	2.49953272
H	0.48808374	-0.80336195	2.48709382
O	-0.19301640	-0.04851766	-2.02291103
C	0.44185622	-0.95585751	-2.72570284
O	1.55771100	-1.39135146	-2.46556980
C	-1.61714462	-2.13784467	-3.44515717
O	-2.28513121	-2.71298284	-4.45773649
O	-2.01884862	-2.19908394	-2.30041731
C	-0.36213981	-1.43345463	-3.93365890
C	-0.59256312	-0.28337481	-4.99048590
C	0.50913842	-2.15978455	-5.00742693
C	0.59717477	-0.83839495	-5.81324223
H	-0.52908103	0.73174860	-4.60007526
H	-1.54596989	-0.41619980	-5.50135522
H	1.43535843	-2.58184574	-4.62308518
H	-0.06322406	-2.92559427	-5.52797048
H	1.52433774	-0.29712298	-5.61713872
H	0.45448121	-0.90732089	-6.89113569
H	-6.69159670	2.88124045	1.07658412
N	-5.90297592	2.40733113	0.65897992
C	-5.56656974	2.36594068	-0.67922512
H	-6.15881728	2.87718357	-1.42402621
N	-4.49090611	1.66418543	-0.92966757
C	-4.08949081	1.21827200	0.30959746
C	-3.03581532	0.42479383	0.74021032
N	-2.88401897	0.15513272	2.03385857
C	-3.79371423	0.64763559	2.90352522
N	-4.84952381	1.40340217	2.61890154
C	-4.95990360	1.67068429	1.32418699
H	-3.64000602	0.39620540	3.94652388
N	-2.09703755	-0.12908018	-0.19897819
H	-2.26504420	0.27135752	-1.12704221
H	-2.25187917	-1.13462966	-0.33816609
H	-3.06944746	-3.14310841	-4.08417278

41
Carbo_2ndS-1

Pt	0.00000000	0.00000000	0.00000000
N	0.00000000	0.00000000	2.08919097
N	2.05127490	0.00000000	-0.40882405
H	2.53886690	0.85418572	-0.14466199
H	2.09934288	-0.08216468	-1.42724752
H	2.56225806	-0.78856389	-0.01578339
H	0.48318656	-0.78967853	2.51389989
H	-0.99054731	-0.08101146	2.33201655
H	0.35403048	0.85377146	2.51692385
O	-0.13134107	-0.12172392	-2.02391161
C	-1.25315938	0.12449686	-2.64004043
O	-1.46428642	-0.29764226	-3.77537855
C	-2.82385189	0.10126608	-0.71926008
O	-2.00536117	-0.11858268	0.27494019
O	-3.96561934	-0.34579264	-0.72477874
C	-2.30164477	0.95400238	-1.88290496
C	-1.78938214	2.39485276	-1.50022094
C	-3.37985662	1.61937212	-2.78264862
C	-2.64203805	2.97594816	-2.65608711
H	-0.71219077	2.55147554	-1.54886988
H	-2.14234200	2.69532404	-0.51248814
H	-3.47944920	1.21785879	-3.78973691
H	-4.35346995	1.61899629	-2.29308544
H	-2.04141625	3.20808931	-3.53668396
H	-3.25723642	3.84204725	-2.41418713
H	-2.88676389	-0.97937068	-4.33935097
N	-5.28930622	-1.47679031	-2.87150388
C	-6.56707042	-2.00425751	-2.79562863
H	-7.14419561	-1.92568853	-1.88692063
N	-6.96447382	-2.57847531	-3.90148288
C	-5.89997824	-2.43093733	-4.75922991
C	-5.70908473	-2.83912831	-6.09707486
N	-6.63966194	-3.49244101	-6.78853975
H	-6.45267461	-3.76215154	-7.74207438
H	-7.52534475	-3.71667919	-6.36168465
N	-4.52785660	-2.55732452	-6.71115061
C	-3.59848085	-1.91550095	-6.05298350
H	-2.65962591	-1.69624008	-6.54792012
N	-3.69417428	-1.48302907	-4.77204768
C	-4.86051065	-1.75163329	-4.12724275
H	-4.78484109	-0.99548362	-2.10226050

41
Carbo_2ndS-2

Pt	0.00000000	0.00000000	0.00000000
N	0.00000000	0.00000000	2.09150395
N	2.04762619	0.00000000	-0.41423877
H	2.54108341	0.84771057	-0.14064960
H	2.08545681	-0.06695332	-1.43454060
H	2.55635809	-0.79764595	-0.03705718
H	-0.99029026	-0.07357021	2.33641120
H	0.36106649	0.85139887	2.51786161
H	0.47821200	-0.79288986	2.51573129
O	-0.11947357	-0.11346064	-2.02371035
C	-1.24982443	0.12271994	-2.64041938
O	-1.48214697	-0.31943345	-3.75720590
C	-2.81118358	0.08948159	-0.73743508
O	-2.01151273	-0.12420610	0.27058424
O	-3.93873569	-0.39862063	-0.78556880
C	-2.28924273	0.96438852	-1.87863734
C	-1.77080636	2.39801221	-1.49003500
C	-3.35967570	1.63646333	-2.78151638
C	-2.60106682	2.98249617	-2.66101076
H	-0.69159302	2.54484562	-1.51985373
H	-2.13933633	2.70492541	-0.50974042
H	-3.46488651	1.22614937	-3.78455301
H	-4.33246036	1.65589795	-2.28937570
H	-1.98677866	3.19181651	-3.53788471
H	-3.20282679	3.86271404	-2.43655274
H	-4.61008583	-1.02418204	-2.24789113
N	-7.98938616	-3.46463553	-5.00505054
C	-7.28055529	-3.55572707	-6.18618365
H	-7.69313021	-4.03731319	-7.05976876
N	-6.09440241	-3.00663885	-6.11386093
C	-6.02263137	-2.53343033	-4.82710061
C	-4.98275692	-1.84723016	-4.16437611
N	-3.83101951	-1.53464336	-4.73552937
H	-3.04356551	-1.05131363	-4.28220651
H	-3.70923755	-1.82316397	-5.69635097
N	-5.27720873	-1.52927794	-2.86677355
C	-6.46634331	-1.85378358	-2.26237405
H	-6.53888674	-1.53912198	-1.22863228
N	-7.45190764	-2.48487620	-2.83110311
C	-7.19023881	-2.80602465	-4.11506786
H	-8.91956425	-3.81294091	-4.81969273

41
Carbo_2ndS-3

Pt	0.00000000	0.00000000	0.00000000
N	0.00000000	0.00000000	2.11548434
N	2.05028964	0.00000000	-0.37834677
H	2.55823203	0.81954460	-0.05106522
H	2.07641549	0.00391887	-1.40295644
H	2.54693842	-0.82970423	-0.05908862
H	-0.97991074	0.04058979	2.39763993
H	0.45741215	0.81633212	2.51758500
H	0.40946953	-0.82716172	2.54623289
O	-0.06095834	0.01009889	-1.99599790
C	-1.04763807	0.58672570	-2.68057904
O	-1.05564297	0.57376784	-3.88523169
C	-2.78460914	0.46299223	-0.80308497
O	-2.03181038	-0.08399233	0.13481450
O	-4.00180351	0.28107273	-0.78566084
C	-2.14348089	1.32914706	-1.87010659
C	-1.60366062	2.73066316	-1.36808974
C	-3.12326355	2.09518367	-2.79717426
C	-2.29563864	3.38876285	-2.58844470
H	-0.52104156	2.82715629	-1.27768054
H	-2.06384564	3.01726040	-0.41984768
H	-3.18835647	1.70656043	-3.81062588
H	-4.11572777	2.15104830	-2.35239913
H	-1.59733582	3.56421492	-3.40696222
H	-2.85080649	4.30595697	-2.39243346
H	-4.92666346	-0.82295827	-0.04080540
N	-7.29687775	-4.91734115	1.60545411
C	-6.19035641	-5.61458194	2.04723381
H	-6.27717150	-6.60036211	2.47874925
N	-5.07028681	-4.95675220	1.88322456
C	-5.46063297	-3.77387765	1.30474916
C	-4.71009059	-2.66082518	0.88585913
N	-3.38941414	-2.55018232	0.99271770
H	-2.88250342	-1.71827277	0.67764359
H	-2.88236651	-3.35064623	1.33853127
N	-5.45017027	-1.65273910	0.34292250
C	-6.81071862	-1.71318303	0.20814593
H	-7.26523939	-0.84340169	-0.25018649
N	-7.55609818	-2.71546046	0.58056596
C	-6.84484797	-3.72408948	1.12046670
H	-8.26073101	-5.22076552	1.62545019

41
Carbo_2ndS-4

Pt	0.00000000	0.00000000	0.00000000
N	0.00000000	0.00000000	2.09391071
N	2.04739905	0.00000000	-0.40757291
H	2.54422245	0.84155066	-0.12138849
H	2.08558318	-0.05278092	-1.42893569
H	2.55206140	-0.80531174	-0.04131503
H	0.47551715	-0.79533849	2.51671349
H	-0.98905190	-0.06807985	2.34369934
H	0.36741401	0.84908357	2.51947927
O	-0.10767669	-0.10638906	-2.01914464
C	-1.22137687	0.15613323	-2.66112472
O	-1.41241439	-0.22615611	-3.80338370
C	-2.80209924	0.08348717	-0.76004734
O	-2.01535078	-0.12372833	0.25541303
O	-3.92878107	-0.41508965	-0.81480875
C	-2.28538885	0.96490278	-1.89398844
C	-1.79066863	2.40613496	-1.49758035
C	-3.35803524	1.62564999	-2.80195627
C	-2.60821335	2.97683767	-2.68409461
H	-0.71315299	2.56751896	-1.50720215
H	-2.18217696	2.70899763	-0.52476765
H	-3.46184714	1.20971508	-3.80275489
H	-4.33052602	1.64535898	-2.30866785
H	-1.98284155	3.17892463	-3.55466180
H	-3.21632197	3.85687005	-2.47668965
H	-4.65106230	-0.99061334	-2.20981324
N	-7.34498315	-2.49228816	-2.87195815
C	-6.49150680	-1.86411315	-2.05029838
H	-6.64498202	-1.67570083	-1.00054463
N	-5.40844528	-1.50160898	-2.72089767
C	-5.54508785	-1.90982218	-4.04711809
C	-4.77187979	-1.84132508	-5.24014765
N	-3.56096244	-1.29044070	-5.36310511
H	-3.14276890	-1.34895431	-6.28029543
H	-2.97562138	-0.92368069	-4.61484617
N	-5.32891651	-2.37923300	-6.34285730
C	-6.53237441	-2.94577691	-6.28773819
H	-6.90572020	-3.35364221	-7.22096135
N	-7.33474736	-3.07581397	-5.22362604
C	-6.78722860	-2.54086659	-4.14573671
H	-8.24853276	-2.87195392	-2.62347323

41
Carbo_2ndS-5

Pt	0.00000000	0.00000000	0.00000000
N	0.00000000	0.00000000	2.09266677
N	2.03517163	0.00000000	-0.26852480
H	2.48922961	0.86661457	0.06371872
H	2.16118263	-0.07064270	-1.28139194
H	2.50533953	-0.79681411	0.15740970
H	-0.11291172	-0.95681260	2.42681977
H	-0.81636190	0.51598991	2.41931969
H	0.83506543	0.39828476	2.56425445
O	-0.00980246	-0.10580570	-2.01409930
C	-1.04742998	0.09804431	-2.79320468
O	-1.04001441	-0.13497415	-3.97513469
C	-2.77396829	0.10237023	-0.90116554
O	-2.05995280	-0.09502660	0.10234144
O	-4.02231771	-0.28779053	-0.78556647
C	-2.30703555	0.78060125	-2.15002036
C	-2.00564972	2.32904186	-1.97083958
C	-3.35397575	1.16194747	-3.23614683
C	-2.70956810	2.56999903	-3.32925895
H	-0.95360016	2.59424988	-1.87704908
H	-2.55832787	2.75214751	-1.12945678
H	-3.33582398	0.54018468	-4.12769373
H	-4.37790205	1.23899357	-2.85232398
H	-1.99991717	2.62679001	-4.15329474
H	-3.38956799	3.41883406	-3.37942085
H	-4.51029544	-0.10296946	-1.60222313
N	4.00771109	0.66489820	5.11309025
C	2.71672353	0.42625368	4.73470567
H	2.03738579	-0.13453367	5.35937618
N	2.41687451	0.94092035	3.56134195
C	3.58763513	1.56412120	3.14763385
C	3.95204080	2.31245431	2.01798625
N	3.06374176	2.58248646	0.97490559
H	3.45349412	3.27478584	0.34249160
H	2.13541348	2.84577341	1.28712605
N	5.20192199	2.74805190	1.90098538
C	6.07980624	2.48312726	2.88522526
H	7.08523081	2.86014754	2.73589222
N	5.85061690	1.82601694	4.02101335
C	4.59892212	1.39090918	4.10257537
H	4.45834152	0.36986858	5.96772039

[CarboPt+H+G]$^+$ complex

```
42
Carbo_G_N7
 Pt                 0.00000000    0.00000000    0.00000000
 N                  0.00000000    0.00000000    2.08288151
 N                  2.07949245    0.00000000   -0.00946377
 H                  2.48233497    0.86162656    0.35425257
 H                  2.32460917   -0.07427916   -1.02585640
 H                  2.50069470   -0.78451193    0.48384082
 H                  0.88369067    0.30010457    2.48872806
 H                 -0.19855215   -0.92917520    2.45065658
 H                 -0.73987760    0.64875963    2.42058498
 O                 -0.11455908   -0.05323220   -2.03926903
 C                  0.83647924   -0.19843641   -2.91328138
 O                  2.04287051   -0.19899780   -2.68368514
 C                 -0.56060856   -1.62539416   -4.37223375
 O                 -1.69045672   -1.71808836   -3.94333984
 O                  0.08212697   -2.66227044   -4.93251458
 C                  0.28724611   -0.36719537   -4.34388447
 C                 -0.42794516    0.93639027   -4.84275607
 C                  1.36241640   -0.16352673   -5.44524224
 C                  0.86139249    1.29434356   -5.62818718
 H                 -0.76883933    1.62282851   -4.06846095
 H                 -1.26979485    0.69636763   -5.49412410
 H                  2.39120165   -0.30883651   -5.12323346
 H                  1.16364259   -0.78872160   -6.31535653
 H                  1.48373642    2.01290244   -5.09220539
 H                  0.72687325    1.64333735   -6.65168139
 H                 -4.68938702   -0.58738183   -1.84382447
 N                 -4.01997357   -0.29498085   -1.14562123
 C                 -2.67939226   -0.50402438   -1.20049667
 H                 -2.19176058   -0.98510998   -2.03654234
 N                 -2.07489247    0.01895269   -0.14540032
 C                 -3.08328799    0.60189257    0.63161253
 C                 -3.07555773    1.32846226    1.86086442
 O                 -2.14447518    1.60392280    2.61644115
 N                 -4.37487419    1.75603568    2.20589617
 H                 -4.41211862    2.26824672    3.07919286
 C                 -5.52612383    1.50865729    1.49316942
 N                 -6.68812455    1.97526207    1.99047386
 H                 -6.73389036    2.59989589    2.77728889
 H                 -7.51831601    1.83669385    1.43679936
 N                 -5.52502912    0.82442832    0.36882080
 C                 -4.30776965    0.41017346   -0.00558259
 H                 -0.52106639   -3.42180491   -4.91705741
```

42
Carbo_G_2ndS-1

Pt	0.00000000	0.00000000	0.00000000
N	0.00000000	0.00000000	2.09774963
N	2.03993217	0.00000000	-0.36521062
H	2.53247495	0.83872475	-0.06365667
H	2.08197334	-0.03956800	-1.38857952
H	2.53872996	-0.81068272	-0.00275801
H	-0.97631317	-0.07845523	2.42295914
H	0.37581301	0.85521302	2.50282108
H	0.50847526	-0.78116887	2.50710478
O	-0.05011886	-0.06549716	-2.00532318
C	-1.12058094	0.16568238	-2.74816328
O	-1.12803238	-0.05640050	-3.93305448
C	-2.79931602	0.23471453	-0.78717588
O	-2.02552936	-0.07476127	0.16073208
O	-4.06578496	0.07876698	-0.61523102
C	-2.34617198	0.85316339	-2.07484115
C	-2.04006207	2.40831729	-1.92255776
C	-3.43597948	1.22880447	-3.11638018
C	-2.81582282	2.64390586	-3.24189609
H	-0.98429922	2.67563353	-1.88051565
H	-2.54974351	2.83064719	-1.05412749
H	-3.43415988	0.60812823	-4.00878097
H	-4.42819520	1.24540927	-2.66931095
H	-2.14756380	2.72059076	-4.09906553
H	-3.50583744	3.48713201	-3.24948937
H	-4.31009297	-0.28223986	0.32830459
N	-6.88825145	-1.55063842	2.66148677
C	-6.30036305	-1.22430916	1.46905306
H	-6.81973940	-1.29345610	0.52587997
N	-5.06167592	-0.82409383	1.62519223
C	-4.81764853	-0.88934328	2.98858511
C	-3.65674957	-0.58804648	3.76925061
N	-3.92083944	-0.80447730	5.14779284
C	-5.10310739	-1.25149347	5.69307973
N	-6.15549488	-1.54312627	4.96225723
C	-5.96094372	-1.34082913	3.64920922
H	-7.83123490	-1.88199024	2.80571531
N	-5.17302877	-1.35902124	7.03957920
O	-2.54571928	-0.19255765	3.43705959
H	-6.01347119	-1.76700270	7.41731997
H	-4.35120382	-1.35539387	7.61956382
H	-3.14438854	-0.56488658	5.75138814

42
Carbo_G_2ndS-2

Pt	0.00000000	0.00000000	0.00000000
N	0.00000000	0.00000000	2.09298962
N	2.03447620	0.00000000	-0.27371625
H	2.51746645	0.85794855	0.06036756
H	2.14413894	-0.05678597	-1.28946967
H	2.49781680	-0.81595415	0.12268170
H	0.80027477	0.47577954	2.53981094
H	-0.03595529	-0.95434088	2.45056802
H	-0.84977516	0.46135958	2.41714266
O	-0.01179916	-0.07275198	-2.01536625
C	-1.04070774	0.17836910	-2.79205626
O	-1.02492568	0.00131288	-3.98397002
C	-2.77139170	0.11212282	-0.90812936
O	-2.06348207	-0.10747521	0.09407570
O	-4.01516166	-0.29933010	-0.81955152
C	-2.30002664	0.83858456	-2.12742613
C	-1.99588316	2.37662508	-1.88265616
C	-3.34267948	1.26780560	-3.19960262
C	-2.69031950	2.67493744	-3.23474890
H	-0.94445097	2.63527384	-1.76607815
H	-2.55514372	2.76501775	-1.02909930
H	-3.32654794	0.68269809	-4.11574943
H	-4.36596414	1.33426530	-2.81257283
H	-1.97574168	2.75730260	-4.05240591
H	-3.36518052	3.52907586	-3.25637003
H	-4.49746312	-0.09424832	-1.63457469
N	3.44826652	4.75873919	0.41751863
C	3.56546361	3.52980071	-0.17113785
H	3.87259181	3.41948837	-1.20054462
N	3.26407007	2.53788351	0.63805574
C	2.93544542	3.15633085	1.83602859
C	2.56128382	2.69008728	3.10340960
N	2.45195456	1.32623020	3.40332072
H	2.39517116	1.19475813	4.40929475
H	3.20882828	0.77647045	3.01008384
N	2.26489467	3.56346167	4.05935373
C	2.36637325	4.87863426	3.79240729
H	2.10992124	5.54725502	4.60635389
N	2.75954748	5.44701101	2.65376669
C	3.03171179	4.54926164	1.71371870
H	3.63357229	5.65753263	-0.00460048

42

Carbo_G_2ndS-3

Pt	0.00000000	0.00000000	0.00000000
N	0.00000000	0.00000000	2.09313977
N	2.04782086	0.00000000	-0.39459953
H	2.53939464	0.84622758	-0.11241441
H	2.09722637	-0.06252881	-1.41493411
H	2.55249546	-0.79961397	-0.01559446
H	0.47832097	-0.79403514	2.51562722
H	-0.98705486	-0.06832409	2.35003639
H	0.36857501	0.85010297	2.51616855
O	-0.10216816	-0.10198682	-2.02130459
C	-1.20196565	0.18475216	-2.67114896
O	-1.37435658	-0.16628000	-3.82897893
C	-2.79906009	0.10978241	-0.76764468
O	-2.01981223	-0.12571020	0.24189775
O	-3.94125766	-0.36140870	-0.83334523
C	-2.27154915	0.98733797	-1.90225490
C	-1.76911679	2.42641752	-1.50082986
C	-3.33605680	1.65877665	-2.81273740
C	-2.57410201	3.00260707	-2.69288230
H	-0.69026993	2.57893074	-1.50083975
H	-2.16681459	2.73092570	-0.53114844
H	-3.44033647	1.24446331	-3.81408394
H	-4.30997635	1.68445844	-2.32349260
H	-1.94150272	3.19676586	-3.55988244
H	-3.17474954	3.88861522	-2.49043020
H	-4.36026784	-1.04425873	-2.42198161
N	-3.54632706	-1.49284692	-5.08140634
C	-3.73255419	-2.01190643	-6.35466377
H	-3.00825998	-1.84379889	-7.13639837
N	-4.83949773	-2.69213847	-6.47232060
C	-5.42298681	-2.63347415	-5.22863383
C	-6.65078491	-3.21977579	-4.75531637
N	-6.83696067	-2.90535621	-3.32386952
C	-6.03850208	-2.17627411	-2.52661687
N	-4.91824142	-1.64587028	-3.04705819
C	-4.62635732	-1.89504110	-4.37191880
H	-2.73801430	-0.96349597	-4.72959492
O	-7.48695751	-3.88474531	-5.29591625
N	-6.31247001	-1.95990808	-1.23683517
H	-5.64893903	-1.43520911	-0.67263081
H	-7.14967545	-2.32182953	-0.81207313
H	-7.67943882	-3.31714643	-2.94025454

42
Carbo_G_2ndS-4

Pt	0.00000000	0.00000000	0.00000000
N	0.00000000	0.00000000	2.09855302
N	2.03830095	0.00000000	-0.37372360
H	2.52653712	0.84633608	-0.08516449
H	2.09477449	-0.06121116	-1.39451433
H	2.53785525	-0.80110959	0.00973207
H	0.50597618	-0.77913890	2.51726666
H	-0.97922587	-0.09967443	2.37417160
H	0.34660727	0.86028456	2.52036899
O	-0.07425983	-0.10175561	-2.01596275
C	-1.14708364	0.17109169	-2.72521919
O	-1.19451558	-0.04539018	-3.92004131
C	-2.77514833	-0.00291655	-0.80422919
O	-2.03126248	-0.14260079	0.21221665
O	-3.93677619	-0.54077765	-0.75158809
C	-2.32279917	0.84536519	-1.97097917
C	-1.92141202	2.31917707	-1.53342555
C	-3.39483988	1.47456710	-2.90498707
C	-2.71282002	2.85544645	-2.74875064
H	-0.85331139	2.52853286	-1.48477990
H	-2.37727521	2.58816694	-0.57914095
H	-3.43881642	1.05512811	-3.90429087
H	-4.38970375	1.44916790	-2.46043406
H	-2.06171398	3.09075642	-3.59129713
H	-3.36621373	3.70585047	-2.55847127
H	-4.28172024	-0.77273994	-1.68481676
N	-6.78884352	-3.28116884	-6.30938305
C	-7.46776676	-3.24769034	-5.09952473
H	-8.46960393	-3.63867953	-5.00519175
N	-6.77106798	-2.70889889	-4.14006580
C	-5.57230806	-2.36703628	-4.73594706
C	-4.41079494	-1.75461266	-4.21015028
C	-3.51553370	-1.96659059	-6.49898232
N	-4.58666417	-2.54086325	-6.99973204
C	-5.56508805	-2.71685350	-6.09286446
H	-7.11767392	-3.64938583	-7.18939799
O	-4.15884395	-1.37075021	-3.05542302
N	-3.41423221	-1.58150685	-5.18323472
H	-2.56470827	-1.12943335	-4.84010525
N	-2.42774075	-1.75997988	-7.28406718
H	-2.55050264	-1.92078462	-8.27065793
H	-1.69505449	-1.13281928	-6.99558823

42
Carbo_G_2ndS-5

Pt	0.00000000	0.00000000	0.00000000
N	0.00000000	0.00000000	2.09690834
N	2.04061512	0.00000000	-0.37247653
H	2.52816570	0.84792498	-0.08728800
H	2.10197412	-0.06637326	-1.39247666
H	2.54069559	-0.79858239	0.01561964
H	0.49311179	-0.78784856	2.51475187
H	-0.98083511	-0.08313727	2.37207209
H	0.36126965	0.85408929	2.51902590
O	-0.08621349	-0.10896485	-2.01617400
C	-1.18672085	0.12494677	-2.69083805
O	-1.32538198	-0.22915140	-3.84533440
C	-2.79324273	0.06494011	-0.79399042
O	-2.03179716	-0.13545788	0.20567303
O	-3.95620394	-0.44566246	-0.74803315
C	-2.30920913	0.90378469	-1.95234895
C	-1.83192495	2.35814745	-1.55519271
C	-3.34501956	1.56328333	-2.90912446
C	-2.56088468	2.89397935	-2.81060566
H	-0.75550351	2.50802856	-1.48335728
H	-2.29872244	2.68726852	-0.62508581
H	-3.43631088	1.10989849	-3.89242877
H	-4.33343993	1.63138074	-2.45746553
H	-1.87914047	3.03196192	-3.65068276
H	-3.14950061	3.80079363	-2.67935071
H	-4.55259457	-0.46736961	-1.63423936
N	-4.30432976	-3.45965380	-6.19204218
C	-3.26523026	-2.73269241	-5.83027907
H	-2.29055758	-2.78292603	-6.29311106
N	-3.51093026	-1.88134319	-4.80024271
C	-4.83920177	-2.07379960	-4.46495493
C	-5.69609140	-1.51380951	-3.50697959
C	-7.38273612	-3.00163784	-4.50945901
N	-6.57657651	-3.52878435	-5.38859575
C	-5.30711377	-3.06459136	-5.35774001
O	-5.49202320	-0.63237175	-2.62808321
N	-6.98255976	-2.03983045	-3.59985813
H	-7.62662404	-1.69929213	-2.89694655
N	-8.67479755	-3.43114423	-4.41476942
H	-8.96047459	-4.04299130	-5.16494115
H	-9.38566957	-2.80659012	-4.06742303
H	-2.83796728	-1.21813456	-4.41700819

[OxaliPt+H+A]$^+$ complex

45

Oxal_N1-1

H	0.00000000	0.00000000	0.00000000
N	0.00000000	0.00000000	1.01003506
C	1.09901157	0.00000000	1.84634445
H	2.10613382	0.02060863	1.45782896
N	0.77880975	-0.02814667	3.11510403
C	-0.59602038	-0.04248759	3.11284702
C	-1.51006715	-0.10492890	4.18298187
N	-2.83787454	-0.09207711	3.84470977
C	-3.19374482	-0.11080945	2.52064532
N	-2.40286662	-0.07654591	1.47757339
C	-1.10399929	-0.02941959	1.81645558
O	-3.44877494	-1.45281710	6.32675626
O	-6.13475334	-2.51897517	5.97655949
C	-3.70479886	-2.72719783	6.37503111
C	-5.16715908	-3.22816477	6.13360682
O	-2.88534037	-3.58115562	6.65195441
O	-5.23464478	-4.54487814	6.15013425
N	-1.10873527	-0.15384081	5.45759951
H	-4.25912157	-0.17928993	2.33428643
H	-1.72948597	-0.57321959	6.14543406
H	-0.11250376	-0.23849753	5.60510907
H	-4.32846905	-4.86441141	6.34077015
C	-6.75029474	1.69773535	4.86527806
C	-7.48124586	2.99805813	4.53052200
C	-8.91254836	2.96165112	5.09726200
C	-8.92242412	2.66344397	6.60310808
C	-8.15678378	1.37107209	6.93595565
C	-6.73490672	1.43364727	6.37272194
H	-9.40442959	3.91607283	4.89776417
H	-6.93367642	3.84525721	4.96347607
H	-7.50353464	3.14911774	3.44699366
H	-7.24419071	0.85633965	4.36883152
H	-8.47315073	3.50232601	7.14740102
H	-9.95044880	2.57862501	6.96173166
H	-8.67605510	0.50646430	6.50500787
H	-6.18827316	2.25049049	6.85838982
H	-9.49125165	2.19726225	4.56627950
N	-5.93052262	0.19558570	6.61298599
H	-5.59642500	0.16432126	7.57524147
H	-6.46101477	-0.67187875	6.46087828
Pt	-4.35799733	0.04387432	5.26632633
N	-5.32018904	1.66274483	4.38408713
H	-5.30134198	1.64754547	3.36464936
H	-4.84413458	2.52384788	4.65982519
H	-8.12008989	1.21459030	8.01774505

45
Oxal_N1-2

H	0.00000000	0.00000000	0.00000000
N	0.00000000	0.00000000	1.00994484
C	1.09898354	0.00000000	1.84630718
H	2.10616042	0.01979243	1.45793516
N	0.77842876	-0.02737613	3.11492401
C	-0.59641937	-0.04113964	3.11259908
C	-1.51000506	-0.10270639	4.18416133
N	-2.83874605	-0.08887730	3.84485593
C	-3.19345686	-0.10556709	2.52091774
N	-2.40334413	-0.07295847	1.47731839
C	-1.10407961	-0.02825149	1.81654740
Pt	-4.35438954	0.04258198	5.27018360
O	-3.42440992	-1.39279230	6.37278129
O	-6.07962230	-2.59095481	6.14054148
C	-3.64608133	-2.68823701	6.47024578
C	-5.03691893	-3.20130123	6.01561156
O	-2.86487062	-3.46487109	6.95140736
O	-4.96206248	-4.43167027	5.52803530
N	-1.10742206	-0.15140632	5.45690019
H	-4.25899360	-0.16990348	2.33442676
H	-1.73177619	-0.55710273	6.15245225
H	-0.11108917	-0.23468381	5.60414021
H	-5.86287918	-4.73288165	5.32696977
C	-6.78689992	1.65170222	4.89990788
C	-7.54839061	2.93570204	4.56887794
C	-8.96273650	2.88684967	5.17517438
C	-8.92645877	2.61017458	6.68464986
C	-8.13237535	1.33421465	7.01337522
C	-6.72728855	1.40848358	6.41061854
H	-9.47436163	3.83094565	4.97610236
H	-7.00203839	3.79692756	4.97483569
H	-7.60236591	3.07166506	3.48444141
H	-7.28187954	0.79631219	4.42889867
H	-8.47447914	3.46306549	7.20431684
H	-9.94265314	2.51586039	7.07346404
H	-8.65083551	0.45643200	6.60847110
H	-6.18068220	2.24076086	6.86922549
H	-9.54453942	2.10648890	4.67129374
N	-5.89739841	0.18730893	6.65059971
H	-5.52316019	0.18525341	7.59875481
H	-6.41971122	-0.69188543	6.55041788
N	-5.37180241	1.63283511	4.37706797
H	-5.38337587	1.60148258	3.35808797
H	-4.90359497	2.50681314	4.62393871
H	-8.06363716	1.19288203	8.09567147

45
Oxal_N1-3

H	0.00000000	0.00000000	0.00000000
N	0.00000000	0.00000000	1.01026840
C	1.09917412	0.00000000	1.84619701
H	2.10619643	0.01874223	1.45723646
N	0.77993383	-0.02610171	3.11548280
C	-0.59460165	-0.04098686	3.11320037
C	-1.51054121	-0.09375910	4.17937666
N	-2.83457271	-0.09303970	3.84374199
C	-3.19522268	-0.10623688	2.51976922
N	-2.40228309	-0.06866244	1.47826670
C	-1.10399534	-0.02840177	1.81613185
O	-3.53775297	-1.79695438	6.04848227
O	-6.39996344	-2.43277789	5.53571545
C	-4.07299410	-2.92973205	6.07940239
C	-5.58851580	-3.33903539	5.78123622
O	-3.36847287	-3.95220171	6.41797673
O	-5.69156809	-4.56763266	5.87584560
N	-1.10592644	-0.11743171	5.45750190
H	-4.26142110	-0.17003973	2.33609292
H	-1.72126704	-0.48636699	6.16820026
H	-0.11087737	-0.20338752	5.61191781
H	-4.06277500	-4.68796749	6.33504802
C	-6.67183448	1.68998193	4.98931910
C	-7.37870834	3.02592516	4.76636626
C	-8.84491183	2.92384737	5.22817298
C	-8.95436120	2.44206088	6.68186866
C	-8.20582588	1.11654851	6.90618621
C	-6.75007567	1.24612425	6.45047617
H	-9.32699404	3.89722168	5.11499250
H	-6.86499952	3.81153489	5.33550804
H	-7.33045292	3.30797737	3.71012759
H	-7.12865516	0.91660282	4.36421866
H	-8.54900708	3.20741710	7.35416622
H	-10.00405448	2.31624298	6.95562353
H	-8.68693686	0.30948608	6.34161218
H	-6.24475945	2.00176540	7.06445285
H	-9.38204258	2.23234382	4.56928761
N	-5.95670753	-0.01280807	6.58916170
H	-5.67730643	-0.14952487	7.55900448
H	-6.46198701	-0.87563486	6.29076056
Pt	-4.35369103	-0.05528389	5.27211315
N	-5.20964710	1.70062851	4.60632101
H	-5.11545349	1.83425531	3.59939386
H	-4.74369521	2.49912181	5.04186131
H	-8.23941970	0.83095200	7.96127722

45
Oxal_N3-1

H	0.00000000	0.00000000	0.00000000
N	0.00000000	0.00000000	1.01756841
C	1.07815289	0.00000000	1.88125936
H	2.07848679	-0.20074404	1.52881723
N	0.74831674	0.26430648	3.11834887
C	-0.61084979	0.47224594	3.07171259
C	-1.53579205	0.83647604	4.07084280
N	-2.82584724	1.03203099	3.70732167
C	-3.17960167	0.84762528	2.45440387
N	-2.39698933	0.45974051	1.41877752
C	-1.08992010	0.31273461	1.77081709
O	-1.36785226	0.68622937	-1.26483501
O	-3.29356551	2.28549783	-2.75647681
C	-1.05833823	1.82179407	-1.81888977
C	-2.15663637	2.64886989	-2.56245903
O	0.06946805	2.27752113	-1.84422913
O	-1.68573641	3.80321710	-2.99196582
H	-4.21830779	1.04599463	2.21528286
N	-1.19187877	1.00603232	5.34882550
H	-1.88579178	1.30239259	6.01687762
H	-0.23195296	0.89027384	5.63391560
H	-0.73801511	3.82608137	-2.74766911
C	-5.91757801	-0.78938290	-0.98172797
C	-7.16283546	-1.64262303	-0.74049419
C	-8.08304760	-1.59514807	-1.97438911
C	-7.34129841	-2.00126736	-3.25567774
C	-6.07135769	-1.16032125	-3.47332623
C	-5.16802796	-1.23130392	-2.23974652
H	-8.94087683	-2.25078967	-1.81041255
H	-6.86130248	-2.67967164	-0.54387476
H	-7.69611924	-1.28854150	0.14695925
H	-6.20425284	0.26074475	-1.09685760
H	-7.07007774	-3.06217876	-3.20350514
H	-8.00001684	-1.89379247	-4.12005611
H	-6.34229052	-0.11417033	-3.66053287
H	-4.83314049	-2.26506904	-2.09470881
H	-8.48416236	-0.58139324	-2.08621690
N	-3.92704971	-0.40338405	-2.34550500
H	-3.23657073	-0.86465456	-2.93622592
H	-4.08929890	0.52360880	-2.75923044
Pt	-3.13971668	0.02448150	-0.47206583
N	-4.93459490	-0.81204307	0.16397985
H	-5.34631050	-0.34645540	0.97169724
H	-4.76760118	-1.77789419	0.45293757
H	-5.52499973	-1.51096996	-4.35346688

45
Oxal_N3-2

H	0.00000000	0.00000000	0.00000000
N	0.00000000	0.00000000	1.01928927
C	1.07437171	0.00000000	1.88652918
H	2.07766030	-0.18709834	1.53503038
N	0.73822736	0.24763912	3.12588517
C	-0.62242835	0.44310725	3.07655946
C	-1.55415664	0.78464572	4.07693175
N	-2.84605482	0.96701429	3.71313853
C	-3.19377583	0.79410708	2.45671032
N	-2.40353733	0.43252406	1.41793461
C	-1.09525865	0.29422506	1.77179305
O	-1.34599941	0.64388777	-1.25921788
O	-3.14643526	2.29398445	-2.85604629
C	-0.98776616	1.79169855	-1.79761720
C	-2.11388945	2.69614018	-2.36037215
O	0.16038574	2.12850621	-1.92770713
O	-1.78204561	3.97690944	-2.28929919
H	-4.23522456	0.97774470	2.21816321
N	-1.21468165	0.94422312	5.35773857
H	-1.91357772	1.22266062	6.02813189
H	-0.25370364	0.83754007	5.64270072
H	-2.48592165	4.49690805	-2.70982399
C	-5.10162045	-1.22695710	-2.31272655
C	-5.96410450	-1.16126067	-3.57557523
C	-7.23889999	-2.00431378	-3.39926058
C	-8.02418573	-1.59627033	-2.14492780
C	-7.14567746	-1.63901078	-0.88117679
C	-5.89346003	-0.78445385	-1.08007724
H	-7.86808416	-1.90122843	-4.28593094
H	-6.23149419	-0.11609466	-3.77405035
H	-5.38767535	-1.51207899	-4.43623192
H	-4.77070717	-2.25995496	-2.15422303
H	-8.42308186	-0.58344481	-2.27297188
H	-8.88608098	-2.25289217	-2.00835731
H	-6.84915293	-2.67510350	-0.67234345
H	-6.17840170	0.26488814	-1.20718654
H	-6.96697724	-3.06431906	-3.33454160
N	-4.95298464	-0.80307856	0.10010683
H	-5.39256934	-0.33059632	0.88865112
H	-4.80118485	-1.76752065	0.40153768
Pt	-3.12583705	0.02203818	-0.48494192
N	-3.85737332	-0.40006838	-2.38160218
H	-3.14198415	-0.86977812	-2.93532576
H	-3.99714131	0.51723524	-2.82232262
H	-7.70897763	-1.28460404	-0.01257620

45
Oxal_N3-3

H	0.00000000	0.00000000	0.00000000
N	0.00000000	0.00000000	1.01203450
C	1.09889594	0.00000000	1.85400294
H	2.09378702	-0.18491799	1.47794067
N	0.78993072	0.24063856	3.09929929
C	-0.57329293	0.42999410	3.08518931
C	-1.48343293	0.75920274	4.11166735
N	-2.78467357	0.93516325	3.77962930
C	-3.16322608	0.76989195	2.53159642
N	-2.39367381	0.41683016	1.47312062
C	-1.07944235	0.28571103	1.79417230
Pt	-3.16271410	0.04768869	-0.42189264
O	-1.51025502	1.05258289	-1.18278004
O	-3.85541480	2.38562712	-2.46381572
C	-1.50878045	2.12185339	-1.83907542
C	-2.72950576	2.90111610	-2.51639351
O	-0.39253729	2.71998956	-2.06495652
O	-2.29256604	3.94077254	-3.02551687
H	-4.21014934	0.95283580	2.31550501
N	-1.11534142	0.91307542	5.38380490
H	-1.80122448	1.18268160	6.07163091
H	-0.14793316	0.81057634	5.64837157
H	-0.72586490	3.52804350	-2.58867732
C	-5.88861787	-0.89936164	-0.84646572
C	-7.08172766	-1.82607957	-0.61958694
C	-8.09397822	-1.66275873	-1.76960740
C	-7.44031621	-1.87846515	-3.14207124
C	-6.21486467	-0.97004091	-3.34486973
C	-5.22042738	-1.15861375	-2.19642144
H	-8.91691924	-2.36653816	-1.62785033
H	-6.73456544	-2.86657046	-0.57655690
H	-7.55692918	-1.60381016	0.34077862
H	-6.21405725	0.14475941	-0.81107280
H	-7.13693288	-2.92710611	-3.24393131
H	-8.16662200	-1.68949674	-3.93539872
H	-6.52399328	0.08095058	-3.38170538
H	-4.85313119	-2.19224787	-2.20253689
H	-8.53160691	-0.65919726	-1.72345099
N	-4.01540070	-0.27919702	-2.28551711
H	-3.35303489	-0.65691955	-2.96076807
H	-4.21893898	0.70290994	-2.57433490
N	-4.81322934	-1.01804656	0.20953997
H	-5.17838961	-0.70653464	1.10923680
H	-4.55935504	-2.00017969	0.33410544
H	-5.72657659	-1.19192236	-4.29789407

45
Oxal_N7-1

H	0.00000000	0.00000000	0.00000000
N	0.00000000	0.00000000	1.01100936
C	1.09389998	0.00000000	1.80620958
H	2.10983465	-0.02664364	1.43812480
N	0.75733360	-0.00534910	3.09093490
C	-0.63594874	-0.04191088	3.12942677
C	-1.61209342	-0.17614410	4.13152042
N	-2.89459555	-0.12698049	3.78471022
C	-3.22311779	-0.01737380	2.48726159
N	-2.39306652	0.00849446	1.44516870
C	-1.12157387	-0.01003874	1.81647372
O	3.32163966	-0.85924270	3.29613321
O	6.28403231	-1.62627861	1.78979673
C	4.59539331	-0.64544719	3.16743582
C	5.06938329	-1.08487478	1.76652146
O	5.35790383	-0.10837033	3.95295622
O	4.40461365	-0.89603957	0.77894384
H	-4.28346767	0.03081090	2.26752398
N	-1.30464623	-0.34087606	5.47990181
H	-0.57722722	-1.02901430	5.64772091
H	-2.14364804	-0.54404985	6.01421644
H	6.53564628	-1.83905797	0.87665086
C	1.78351841	1.58792834	7.18685010
C	1.33039447	2.80104573	8.00036480
C	2.14108818	2.90281698	9.30515055
C	3.65312330	2.91435300	9.03960126
C	4.09686837	1.70283198	8.20125798
C	3.28852121	1.62948349	6.90439766
H	1.84581772	3.80504386	9.84523417
H	1.47751641	3.71058363	7.40342211
H	0.26062285	2.72984784	8.21759362
H	1.56397176	0.67145802	7.74626598
H	3.92772483	3.83695744	8.51523990
H	4.20047019	2.92266234	9.98465904
H	3.94463685	0.77954176	8.77542186
H	3.50516979	2.51096612	6.29126578
H	1.88789450	2.05665514	9.95454990
N	3.61315718	0.44764083	6.04990300
H	4.50870162	0.52077072	5.54111969
H	3.67942791	-0.39609266	6.62075758
Pt	2.16500097	0.20503630	4.59132423
N	1.06809061	1.44168592	5.87179782
H	0.11191571	1.08704571	6.00738046
H	0.98092360	2.35837686	5.43112551
H	5.16373776	1.76314476	7.96995431

45

Oxal_N7-2

H	0.00000000	0.00000000	0.00000000
N	0.00000000	0.00000000	1.01129308
C	1.09363529	0.00000000	1.80468834
H	2.10895807	-0.02973120	1.43679404
N	0.75964884	-0.01080978	3.09091969
C	-0.63435564	-0.05612791	3.13071289
C	-1.61102048	-0.21327742	4.12877061
N	-2.89320095	-0.16484289	3.78261747
C	-3.22282827	-0.03962786	2.48687837
N	-2.39182907	-0.00172262	1.44616111
C	-1.12078940	-0.01766836	1.81748031
Pt	2.17049531	0.19610202	4.59269184
O	3.35483683	-0.75205418	3.23522917
O	4.46105861	-1.03629123	0.77797076
C	4.63088999	-0.61505932	3.15679555
C	5.17380363	-0.94087435	1.73713777
O	5.41734195	-0.23773494	4.02340562
O	6.49984244	-1.04183495	1.70451163
H	-4.28317668	0.00862664	2.26742854
N	-1.30526934	-0.40570967	5.47641609
H	-0.59668545	-1.11736193	5.62779827
H	-2.15010606	-0.60913622	6.00205990
H	6.83409391	-0.84683521	2.59698666
C	1.75191572	1.45498182	7.24442298
C	1.27111885	2.62299778	8.10672318
C	2.07111961	2.68107292	9.42083416
C	3.58445586	2.73099194	9.16769198
C	4.05571118	1.56574970	8.27980541
C	3.25751526	1.53673547	6.97505351
H	1.75626533	3.55333245	9.99772013
H	1.40541872	3.56048190	7.55154442
H	0.20150272	2.52276996	8.31257870
H	1.54643733	0.51139765	7.76270961
H	3.84627454	3.68066412	8.68699383
H	4.12510390	2.70656197	10.11625735
H	3.91595952	0.61529867	8.81113402
H	3.46256143	2.44762198	6.40242000
H	1.82854801	1.80292325	10.03054214
N	3.60574369	0.39739127	6.07184735
H	4.51632970	0.48922705	5.59904818
H	3.66161289	-0.47078598	6.60666760
N	1.04715622	1.35149356	5.91978115
H	0.09514278	0.97509823	6.03328601
H	0.94745639	2.28420782	5.51662678
H	5.12307847	1.65544180	8.06027037

45

Oxal_N7-3

H	0.00000000	0.00000000	0.00000000
N	0.00000000	0.00000000	1.01039777
C	1.08107576	0.00000000	1.82162209
H	2.09475638	0.00476691	1.45664329
N	0.73006481	0.01437279	3.09989355
C	-0.66963867	0.05585051	3.12383046
C	-1.67531627	0.16280293	4.12456588
N	-2.95226722	0.06754078	3.72096489
C	-3.24048744	-0.03274394	2.42216395
N	-2.39005818	-0.02843880	1.39127309
C	-1.13167909	0.01386698	1.80493503
Pt	2.06465817	-0.14170725	4.66379772
O	1.19440431	1.54476345	5.38895507
O	-0.32231952	3.25268671	6.87133226
C	1.82214236	2.38447169	6.16865740
C	0.84689615	3.46454251	6.68746316
O	2.98263942	2.33993693	6.52318392
O	1.47740591	4.61219834	6.93432001
H	-4.29415946	-0.10762691	2.17530009
N	-1.43112170	0.34391034	5.43807228
H	-2.25956976	0.52047464	5.99019502
H	-0.59974912	0.86421496	5.70268714
H	0.82401446	5.23958615	7.28285142
C	4.30419475	-2.05934242	4.66261570
C	4.92631206	-3.44815859	4.51428311
C	6.28440209	-3.50085948	5.23819867
C	6.16428383	-3.07535734	6.70823300
C	5.50713798	-1.69158592	6.85202572
C	4.15639187	-1.66583837	6.13305206
H	6.69146575	-4.51188007	5.16958280
H	4.24903671	-4.19765925	4.94372190
H	5.04862880	-3.69487086	3.45520098
H	4.93488744	-1.31637402	4.16367314
H	5.57344229	-3.81641635	7.25899971
H	7.15099105	-3.05878667	7.17587555
H	6.15904022	-0.92183582	6.42050585
H	3.47808079	-2.38177050	6.61167400
H	6.99440636	-2.84493775	4.72145639
N	3.46191850	-0.34053840	6.19372189
H	3.02006019	-0.21095917	7.10278503
H	4.09830826	0.45670247	6.11384262
N	2.94570510	-1.92703541	4.02313351
H	3.03426772	-2.00171213	3.01040584
H	2.35400311	-2.70945433	4.30938665
H	5.37063468	-1.43878518	7.90714937

45
Oxal_N7-4

H	0.00000000	0.00000000	0.00000000
N	0.00000000	0.00000000	1.01051946
C	1.08014018	0.00000000	1.82153035
H	2.09372617	0.00322087	1.45626196
N	0.72902621	0.01685632	3.10044680
C	-0.67154459	0.06109910	3.12402878
C	-1.67972641	0.17644785	4.12178738
N	-2.95527078	0.07766068	3.71620899
C	-3.24201911	-0.02857899	2.41781041
N	-2.38939260	-0.02683225	1.38863870
C	-1.13226604	0.01607526	1.80443242
Pt	2.07269704	-0.14955208	4.65587327
O	1.20149308	1.51160709	5.43872144
O	-0.27187189	3.46575107	6.62058482
C	1.84855749	2.40348976	6.11579611
C	0.92186813	3.51731572	6.68020790
O	3.04831667	2.43242528	6.36364942
O	1.61254943	4.50086792	7.25418084
H	-4.29515006	-0.10574040	2.16961228
N	-1.43946573	0.36950728	5.43484827
H	-2.27180075	0.55425140	5.97892298
H	-0.61306791	0.90041120	5.69338676
H	2.55644112	4.27221955	7.19725223
C	4.34189369	-2.02331089	4.60613411
C	4.98832526	-3.39812507	4.43248142
C	6.35712077	-3.43319205	5.13706343
C	6.24928892	-3.02836071	6.61379401
C	5.56737586	-1.65948365	6.78399275
C	4.20671122	-1.65171091	6.08379279
H	6.78223286	-4.43529274	5.05034335
H	4.33153057	-4.16571897	4.86166574
H	5.10073985	-3.62935988	3.36886935
H	4.95153895	-1.26246787	4.10805173
H	5.68088324	-3.78745464	7.16350528
H	7.24183358	-2.99779673	7.06807405
H	6.19830682	-0.87196578	6.35328445
H	3.54930283	-2.38696738	6.56208605
H	7.04733350	-2.75767922	4.61880118
N	3.48493014	-0.34093811	6.17298187
H	3.04835543	-0.24255529	7.08864764
H	4.10472446	0.47010937	6.09478406
N	2.97243778	-1.91009056	3.98482811
H	3.04960870	-1.97443280	2.97037642
H	2.39833171	-2.70569153	4.27098664
H	5.44049626	-1.42316222	7.84418222

45
Oxal_N7-5

H	0.00000000	0.00000000	0.00000000
N	0.00000000	0.00000000	1.01090737
C	1.08801926	0.00000000	1.81571194
H	2.10282312	0.00048189	1.45334539
N	0.73810029	-0.00152546	3.09240672
C	-0.65805902	-0.01953737	3.12443819
C	-1.63922658	-0.06052064	4.14514321
N	-2.92039618	0.00747716	3.76374561
C	-3.23010192	0.05846216	2.46238014
N	-2.39570469	0.03807997	1.41989664
C	-1.12967209	0.00248934	1.80992763
O	2.51114725	-1.93691750	4.25468689
O	0.83095276	-1.84576394	6.63832033
C	2.07011098	-2.93512528	4.85918071
C	1.13996662	-2.95528226	6.14793584
O	2.37737298	-4.12144816	4.45285543
O	0.88104648	-4.11583327	6.46637603
H	-4.28828650	0.11075282	2.23088681
N	-1.36267926	-0.11461339	5.47963603
H	-0.56172350	-0.66129073	5.80327134
H	-2.19595599	-0.24149099	6.04168848
H	1.89079796	-4.69899225	5.11097209
C	1.70814915	2.07567128	6.80189150
C	1.68266689	3.52676887	7.28154403
C	1.99234578	3.59005648	8.78860279
C	3.31485255	2.88911458	9.12942903
C	3.34282326	1.43899690	8.61504489
C	3.04487173	1.40142473	7.11431921
H	2.02528638	4.63422149	9.10672764
H	2.43048182	4.10762068	6.72605369
H	0.70623815	3.97392674	7.07308991
H	0.90874496	1.50933014	7.28974322
H	4.15101178	3.44639865	8.69121044
H	3.47410961	2.89313026	10.20976138
H	2.59287510	0.83956984	9.14529224
H	3.83784088	1.92692696	6.57012119
H	1.17258633	3.12157303	9.34485371
N	2.99364934	0.01873700	6.54007560
H	3.92833649	-0.38314697	6.48523210
H	2.41240349	-0.61078228	7.11134986
Pt	1.95201587	0.00776221	4.74149456
N	1.44837242	1.92449910	5.32210008
H	0.46009793	2.10172472	5.13773947
H	1.98058946	2.62088825	4.79824389
H	4.31638082	0.98101471	8.81199577

45
Oxal_NH2

H	0.00000000	0.00000000	0.00000000
N	0.00000000	0.00000000	1.01040527
C	1.10039246	0.00000000	1.84477701
H	2.10595795	0.01230842	1.45033002
N	0.79560874	-0.01721255	3.11711211
C	-0.58035425	-0.02814624	3.12806645
C	-1.52651158	-0.06471788	4.14027475
N	-2.82505207	-0.07683508	3.86150310
C	-3.19228972	-0.07259962	2.56181189
N	-2.39300308	-0.04416342	1.49777529
C	-1.10604271	-0.02012394	1.81762008
O	0.10628893	1.02709777	7.64461776
O	-1.57262449	1.06418593	10.06138658
C	0.48328087	0.52781713	8.77984417
C	-0.46970895	0.56508056	10.02446703
O	1.57408771	0.02463337	8.97838365
O	0.08540733	-0.02157902	11.06466156
N	-1.13293309	-0.10888829	5.52750137
H	-4.25845246	-0.09770207	2.36808724
H	-1.49772690	-0.96445132	5.95064215
H	-0.11092995	-0.13861186	5.60368548
H	0.97363875	-0.31217360	10.76401819
C	-4.43183113	2.51242647	7.02696666
C	-5.73181513	3.11126645	6.48982854
C	-6.53080006	3.75291764	7.63915965
C	-5.69518070	4.78413424	8.41125115
C	-4.37077212	4.18704370	8.91944800
C	-3.59325958	3.56363639	7.75886853
H	-7.43096422	4.22261634	7.23674758
H	-5.49814746	3.87064099	5.73220793
H	-6.32585140	2.33648280	5.99630637
H	-4.65976269	1.69850958	7.72221657
H	-5.48109829	5.64259331	7.76379593
H	-6.26632158	5.17150102	9.25770024
H	-4.57129632	3.41593906	9.67296400
H	-3.32737612	4.34705118	7.03913594
H	-6.87043668	2.96749710	8.32396813
N	-2.30320036	2.92024099	8.16893200
H	-1.57458898	3.62306462	8.28569632
H	-2.36320882	2.41337280	9.06334695
Pt	-1.72061641	1.45049883	6.83265664
N	-3.54872951	1.91105941	5.97207169
H	-3.95476515	1.08075542	5.53388470
H	-3.41528002	2.58083143	5.21235543
H	-3.76410684	4.95713020	9.40428417

45
Oxal_2ndS-1

N	0.00000000	0.00000000	0.00000000
C	0.00000000	0.00000000	1.38058522
H	0.91859328	0.00000000	1.94757675
N	-1.20471439	-0.00025055	1.89212646
C	-2.03328889	-0.00012078	0.79649313
C	-3.44064694	0.00005937	0.70970787
N	-4.26167295	-0.00020254	1.74642852
H	-5.27602820	0.00228150	1.61734414
H	-3.85760471	0.00029533	2.67072329
N	-3.91654279	0.00017722	-0.57109082
C	-3.10516934	0.00024810	-1.67423138
H	-3.63688406	0.00027528	-2.61761225
N	-1.80238611	0.00022596	-1.64985166
C	-1.30755437	0.00004462	-0.39513080
H	0.80258764	0.00008374	-0.61345487
O	-9.08395123	0.00790336	0.38261780
O	-8.49737943	-0.00142136	-2.22789524
C	-7.81789107	0.00755233	0.09572622
C	-7.47876896	0.00010679	-1.42710097
O	-6.92099655	0.01231485	0.92063747
O	-6.31323908	-0.00341927	-1.79602099
H	-4.94157744	-0.00011471	-0.77349309
C	-12.92013337	-0.33766103	-2.54799813
C	-13.96641752	0.02387636	-3.60419174
C	-15.37926000	-0.32462260	-3.10432195
C	-15.68157364	0.33169350	-1.75009158
C	-14.61381787	-0.01440375	-0.69761269
C	-13.21852311	0.34802880	-1.21011200
H	-16.11533535	-0.01124255	-3.84788269
H	-13.90556018	1.09854026	-3.81967712
H	-13.74966329	-0.50254503	-4.53832179
H	-12.92068733	-1.42139699	-2.38902930
H	-15.73077524	1.42006115	-1.87122413
H	-16.66304192	0.01664478	-1.38905752
H	-14.64891584	-1.08889486	-0.47560950
H	-13.15186427	1.43178785	-1.35426202
H	-15.47318063	-1.41313313	-3.01510488
N	-12.11113688	-0.00172769	-0.25753052
H	-12.09384659	0.65004374	0.52616479
H	-12.27108797	-0.92653138	0.14459642
Pt	-10.28310278	0.00393187	-1.25687974
N	-11.51306477	0.01234998	-2.94014084
H	-11.16525758	-0.63865611	-3.64329189
H	-11.48698337	0.93769993	-3.37087664
H	-14.81464154	0.51266180	0.23971345

45
Oxal_2ndS-2

N	0.00000000	0.00000000	0.00000000
C	0.00000000	0.00000000	1.38328428
H	0.92317676	0.00000000	1.94239333
N	-1.20077845	0.00009831	1.90205466
C	-2.04235855	0.00002653	0.81237783
C	-3.45017945	-0.00007669	0.70437570
N	-4.25321660	-0.00018955	1.76699692
H	-5.25258084	-0.00028742	1.63360910
H	-3.86473760	-0.00018922	2.69727859
N	-4.01626499	-0.00006386	-0.53208651
C	-3.24822420	0.00001033	-1.59278909
H	-3.70590214	0.00002012	-2.57496974
N	-1.89520537	0.00003824	-1.58565543
C	-1.30287930	-0.00001026	-0.36695396
H	0.78029139	-0.00034550	-0.65859870
O	2.48599780	-0.00460059	-4.25336475
O	0.35686766	-0.00780769	-5.87746109
C	1.33173942	-0.00388289	-3.66229150
C	0.09185452	-0.00416582	-4.60936440
O	1.16428329	-0.00305331	-2.45434909
O	-1.03777667	-0.00110237	-4.14147824
H	-1.37431157	0.00010494	-2.49677144
C	3.52569286	0.32289041	-8.97365005
C	3.64107535	-0.04752110	-10.45363597
C	5.04148496	0.29968055	-10.98836154
C	6.14899562	-0.34889733	-10.14637986
C	6.01473366	0.00727666	-8.65553757
C	4.61986194	-0.35545876	-8.14204524
H	5.12256420	-0.02092142	-12.02926518
H	3.45687085	-1.12319869	-10.57194791
H	2.87088020	0.47424638	-11.02915672
H	3.62635483	1.40754679	-8.85918924
H	6.11014910	-1.43816182	-10.26301219
H	7.13048853	-0.03459073	-10.50796270
H	6.18227797	1.08318564	-8.51636946
H	4.47847638	-1.43989784	-8.20292741
H	5.16901974	1.38842168	-10.98877747
N	4.38985508	0.00154199	-6.70085386
H	4.88671186	-0.64500934	-6.08902653
H	4.76854014	0.92895939	-6.50322315
Pt	2.34855348	-0.00951859	-6.28285408
N	2.19964850	-0.02681081	-8.36063766
H	1.47805001	0.62137652	-8.67453072
H	1.90274665	-0.95407470	-8.66833780
H	6.77545526	-0.51394197	-8.06701792

45
Oxal_2ndS-3

N	0.00000000	0.00000000	0.00000000
C	0.00000000	0.00000000	1.34651642
H	0.86384519	0.00000000	1.99052463
N	-1.24720857	0.00008202	1.78391664
C	-2.10482471	0.00034080	0.69607455
C	-3.51095900	0.00096553	0.52263272
N	-4.38932966	0.00130609	1.53316651
H	-5.36930731	0.00174074	1.29716354
H	-4.09815240	0.00076568	2.50453142
N	-3.95780001	0.00137490	-0.74472729
C	-3.08594083	0.00116481	-1.75740362
H	-3.52186669	0.00156361	-2.75063891
N	-1.74783389	0.00062219	-1.70408058
C	-1.31810019	0.00026919	-0.45105384
H	0.81679479	-0.00006013	-0.59537403
O	-3.03934907	0.00246513	6.24748515
O	-0.37653834	0.00523114	6.59590524
C	-2.32929960	0.00151724	5.17095966
C	-0.78001586	0.00425303	5.35875305
O	-2.78618062	-0.00135950	4.03035161
O	-0.05971082	0.00530022	4.37926379
H	-1.52839737	0.00007898	2.78523783
C	-1.55450188	0.35620170	10.85231937
C	-0.90163560	-0.00425295	12.18811641
C	-1.83765610	0.34896127	13.35726941
C	-3.21800669	-0.30335141	13.19825329
C	-3.85986816	0.04231679	11.84311596
C	-2.91779875	-0.32585469	10.69515233
H	-1.37857780	0.03538412	14.29733230
H	-0.68096316	-1.07945281	12.20370579
H	0.05306997	0.52004243	12.28960849
H	-1.70104252	1.44023413	10.79726462
H	-3.12336979	-1.39185675	13.28661719
H	-3.88076817	0.01527350	14.00574985
H	-4.07733088	1.11747240	11.80108273
H	-2.76297972	-1.41010027	10.68286836
H	-1.94927218	1.43790124	13.41522771
N	-3.45131886	0.02139446	9.33400037
H	-4.18840331	-0.62891554	9.06398268
H	-3.88041935	0.94773719	9.35063636
Pt	-1.89875042	0.00645891	7.93971722
N	-0.72219074	-0.00031965	9.65342467
H	0.05871653	0.64753857	9.55321525
H	-0.30989049	-0.92683013	9.77323295
H	-4.81279413	-0.48193552	11.72567500

45
Oxal_2ndS-4

N	0.00000000	0.00000000	0.00000000
C	0.00000000	0.00000000	1.38014758
H	0.91906219	0.00000000	1.94647938
N	-1.20473231	0.00012914	1.89320788
C	-2.03305882	-0.00026193	0.79738784
C	-3.43680552	-0.00052216	0.70305467
N	-4.26557516	-0.00066660	1.74215073
H	-5.28539250	0.00356273	1.64165596
H	-3.84903424	-0.00594447	2.66046018
N	-3.91485153	-0.00063742	-0.57316017
C	-3.10579300	-0.00072667	-1.67649833
H	-3.62656394	-0.00167465	-2.62631057
N	-1.80232322	-0.00013562	-1.64844804
C	-1.30696164	-0.00010813	-0.39582852
H	0.80329168	-0.00016371	-0.61321432
Pt	-8.92702791	0.03447649	2.51824824
O	-9.78690804	-0.04790891	0.70901404
O	-7.19711970	0.01999147	1.39723026
C	-8.94868843	-0.06588551	-0.31070795
C	-7.44861789	-0.02274280	0.11004663
O	-9.26184157	-0.11256518	-1.46699789
O	-6.57031301	-0.03198505	-0.73932828
H	-4.96076048	-0.00668789	-0.69428560
C	-10.53991180	-0.26639738	4.97591446
C	-11.73897964	0.09529123	5.85534356
C	-11.43437307	-0.20710192	7.33259259
C	-10.14979078	0.48947569	7.80217391
C	-8.95167100	0.14292866	6.90074951
C	-9.27065509	0.45703787	5.43768851
H	-12.27964951	0.10585865	7.94930340
H	-11.96553037	1.16264355	5.73578851
H	-12.62260520	-0.45807948	5.52467589
H	-10.36224877	-1.34602458	5.02534878
H	-10.29923779	1.57541583	7.80356328
H	-9.92260831	0.20776204	8.83268252
H	-8.71515501	-0.92485960	6.99695198
H	-9.42653278	1.53486686	5.32046151
H	-11.33672750	-1.29050670	7.46797062
N	-8.16151167	0.10449583	4.48435383
H	-7.40411980	0.77875357	4.57810321
H	-7.77227279	-0.80568868	4.73260184
N	-10.73705607	0.04569377	3.52273954
H	-11.37016601	-0.62469049	3.08642490
H	-11.17288213	0.96252984	3.41063407
H	-8.06287980	0.69783370	7.21605415

[OxaliPt+H+G]$^+$ complex

```
Oxal_G_N7
46
 H                   0.00000000    0.00000000    0.00000000
 N                   0.00000000    0.00000000    1.01045960
 C                   1.10085498    0.00000000    1.80703216
 H                   2.11445918   -0.03232855    1.43603282
 N                   0.75944094   -0.02209998    3.08506609
 C                  -0.63657175   -0.04324185    3.11648767
 C                  -1.58658055   -0.10402613    4.18318146
 O                  -1.42128045   -0.11582152    5.40022622
 N                  -2.90522991   -0.15033574    3.67692526
 C                  -3.26759018   -0.13386198    2.34910160
 N                  -4.58278876   -0.15930492    2.05451060
 H                  -5.29177074   -0.34001733    2.74454751
 H                  -4.83705186   -0.20979349    1.08088100
 N                  -2.38292585   -0.07469726    1.37642301
 C                  -1.11555454   -0.03581093    1.80828438
 Pt                  2.19296656   -0.14563942    4.58578293
 O                   3.34914361   -0.90499439    3.08105124
 O                   4.43913982   -0.68838171    0.60380880
 C                   4.63144780   -0.73377334    3.01490268
 C                   5.11982119   -0.94814776    1.56395261
 O                   5.40160049   -0.39749405    3.89862996
 O                   6.37133677   -1.40152143    1.51828715
 H                   6.62379824   -1.47966446    0.58458357
 H                  -3.61357979   -0.18298267    4.40033213
 C                   1.79131760    0.44423665    7.44602661
 C                   1.32568949    1.35568194    8.58254977
 C                   2.10711623    1.04420951    9.87153663
 C                   3.62518139    1.12080166    9.65373144
 C                   4.08397080    0.22018624    8.49344382
 C                   3.30255800    0.55441694    7.22192776
 H                   1.80397539    1.73952569   10.65766660
 H                   1.49004790    2.40282271    8.29602601
 H                   0.25111829    1.23203636    8.74392524
 H                   1.55287270   -0.59592903    7.69402969
 H                   3.91423126    2.15701673    9.44254986
 H                   4.15127271    0.83415957   10.56709758
 H                   3.91585372   -0.83284190    8.75442367
 H                   3.53577961    1.57833440    6.90995360
 H                   1.83677642    0.04182818   10.22371506
 N                   3.63804598   -0.31598815    6.05473239
 H                   4.53945586   -0.10382124    5.60172335
 H                   3.69553214   -1.29185268    6.34928544
 N                   1.11347323    0.71515594    6.13739626
 H                   0.13990375    0.37051571    6.11537363
 H                   1.06911383    1.72286169    5.98184256
 H                   5.15617037    0.33794264    8.31386500
```

46
Oxal_G_2ndS-1

N	0.00000000	0.00000000	0.00000000
C	0.00000000	0.00000000	1.39166668
H	0.92279239	0.00000000	1.95230074
N	-1.19339952	0.00001252	1.90594801
C	-2.03511959	-0.00005051	0.80945702
C	-3.42148918	0.00019763	0.69062643
N	-3.90425131	-0.00000668	-0.58939037
C	-3.09308753	-0.00056143	-1.71308842
N	-1.77368178	-0.00042763	-1.64492752
C	-1.30297677	-0.00007428	-0.39561473
H	0.80072197	-0.00006285	-0.61472790
O	-8.60377232	-0.02644096	-2.04450822
O	-8.93909411	-0.00815956	0.61014699
C	-7.51988685	-0.01327366	-1.34365642
C	-7.71184073	0.00068890	0.20423549
O	-6.38643703	-0.01103725	-1.80900897
O	-6.73882157	0.01992873	0.94190859
H	-5.19064592	0.00475304	1.44344755
O	-4.23286042	0.00013371	1.70496084
N	-3.71783733	-0.00113492	-2.90041377
H	-4.72761034	-0.00435625	-2.96631427
H	-3.14568619	-0.00231674	-3.72799280
H	-4.92563611	0.00011649	-0.75593217
C	-13.21152079	0.28947375	-0.55769504
C	-14.54232470	-0.08061661	0.09981477
C	-15.71571764	0.25174779	-0.83865585
C	-15.55032478	-0.41033795	-2.21355021
C	-14.19967532	-0.05484905	-2.85983378
C	-13.04774295	-0.40212978	-1.91492729
H	-16.65185705	-0.06857378	-0.37633488
H	-14.54560015	-1.15398083	0.32960580
H	-14.64902767	0.45076319	1.04996479
H	-13.16860438	1.37285672	-0.71287344
H	-15.62644823	-1.49891329	-2.10991574
H	-16.36164295	-0.10621667	-2.87839090
H	-14.16992694	1.01882205	-3.08638332
H	-13.02194244	-1.48485062	-1.75146115
H	-15.78601404	1.33897565	-0.95915430
N	-11.69023240	-0.04259229	-2.45161472
H	-11.41311149	-0.69422916	-3.18509025
H	-11.71799149	0.88088208	-2.88665068
Pt	-10.29567375	-0.03122402	-0.90487496
N	-12.00699562	-0.04743337	0.27533709
H	-11.91357564	0.60721433	1.05157694
H	-12.11632743	-0.97210578	0.69493457
H	-14.07705560	-0.58605755	-3.80821898

46
Oxal_G_2ndS-2

N	0.00000000	0.00000000	0.00000000
C	0.00000000	0.00000000	1.38703545
H	0.92112845	0.00000000	1.94872763
N	-1.20237046	0.00006020	1.89296250
C	-2.04493804	0.00009361	0.80454518
C	-3.48392284	0.00012721	0.74044087
N	-3.92005942	0.00019713	-0.66794741
C	-3.15688005	0.00032379	-1.77760389
N	-1.82156785	0.00015814	-1.63572404
C	-1.30314919	0.00006525	-0.36203750
H	0.77928362	-0.00019438	-0.65255387
O	2.28718281	-0.00386382	-4.39904973
O	0.21290853	-0.00593696	-6.10340169
C	1.11279341	-0.00325267	-3.85457369
C	-0.09015917	-0.00289896	-4.84863268
O	0.88706147	-0.00289367	-2.65465935
O	-1.22944018	0.00028530	-4.39763934
H	-1.21546251	0.00026089	-2.47051219
O	-4.32860190	0.00007690	1.59048660
N	-3.68594119	0.00059021	-3.00308449
H	-4.68274484	0.00041637	-3.13860418
H	-3.06633212	0.00038288	-3.81016250
H	-4.92858180	0.00045115	-0.76224161
C	3.52073363	0.32775005	-9.06965199
C	3.70209460	-0.03920156	-10.54374609
C	5.12501421	0.31146918	-11.01328102
C	6.19433619	-0.33815191	-10.12414981
C	5.99294359	0.01355485	-8.63956251
C	4.57649661	-0.35180954	-8.19168771
H	5.25330607	-0.00616436	-12.05027554
H	3.52506948	-1.11482916	-10.67282279
H	2.95817561	0.48331965	-11.15218523
H	3.61463684	1.41225751	-8.94849988
H	6.16280988	-1.42709160	-10.24552747
H	7.19055906	-0.02119634	-10.43999141
H	6.15322066	1.08910703	-8.48973301
H	4.43924886	-1.43629259	-8.26046727
H	5.25069017	1.40035462	-11.00516335
N	4.27993964	0.00294234	-6.76140949
H	4.74871967	-0.64427720	-6.12815886
H	4.64924733	0.93019458	-6.54516466
N	2.16832266	-0.02486331	-8.51607262
H	1.46114752	0.62180636	-8.86418329
H	1.88834883	-0.95283079	-8.83750578
H	6.72657042	-0.50923299	-8.01901221
Pt	2.22633974	-0.00801366	-6.43468614

46
Oxal_G_2ndS-3

N	0.00000000	0.00000000	0.00000000
C	0.00000000	0.00000000	1.32460179
H	0.88979347	0.00000000	1.93706251
N	-1.22837686	-0.00000000	1.88944074
C	-2.12212182	0.00224812	0.83735804
C	-3.49734876	0.00763788	0.69774851
N	-3.93828844	0.00919530	-0.60331855
C	-3.09726883	-0.00168138	-1.70290576
N	-1.79590875	-0.00926838	-1.59534227
C	-1.31127246	0.00157576	-0.33922037
Pt	-3.25027734	0.06897970	7.87346302
O	-1.50892066	0.03826856	6.83273627
O	-4.06259990	0.05749136	5.99559420
C	-1.67787420	0.02781966	5.54214265
C	-3.16347892	0.03819137	5.07583296
O	-0.79182611	0.01139727	4.71155231
O	-3.38410232	0.02779936	3.86714984
H	-4.14008045	0.02193137	2.52633678
O	-4.46318404	0.01635892	1.57493518
N	-3.68743819	0.04457506	-2.92476823
H	-4.63277443	-0.27579297	-3.05991086
H	-3.05800613	-0.06100925	-3.70618250
H	-4.94267531	0.06361177	-0.72197577
H	-1.40968357	0.00307148	2.90074609
C	-4.74268303	-0.22111978	10.40885261
C	-5.86724182	0.16932336	11.36990466
C	-5.47684566	-0.16655915	12.82000975
C	-4.13838830	0.47439767	13.21228325
C	-3.01591409	0.09857988	12.22915185
C	-3.42052971	0.44993369	10.79616501
H	-6.26730398	0.16778350	13.49544402
H	-6.06180180	1.24592810	11.27983408
H	-6.79071321	-0.34731978	11.09234940
H	-4.60023004	-1.30682702	10.43480217
H	-4.24592278	1.56494265	13.23901073
H	-3.85483991	0.16749080	14.22133340
H	-2.81353935	-0.97838748	12.29355774
H	-3.54249348	1.53463971	10.70590081
H	-5.41153657	-1.25479701	12.93423781
N	-2.39543263	0.07144534	9.76517854
H	-1.60161076	0.71087008	9.79499378
H	-2.02007853	-0.85726341	9.96416493
N	-5.02549774	0.11473905	8.97145158
H	-5.71161288	-0.53188225	8.58396986
H	-5.44041830	1.04518615	8.90367425
H	-2.08790431	0.61493577	12.49134194

```
46
Oxal_G_2ndS-4
 N                  0.00000000    0.00000000    0.00000000
 C                  0.00000000    0.00000000    1.38885611
 H                  0.91854397    0.00000000    1.95480563
 N                 -1.20026680    0.00210838    1.89853652
 C                 -2.04655135    0.00419905    0.81269447
 C                 -3.48674492    0.00877032    0.74093578
 N                 -3.91358684    0.01365771   -0.67371575
 C                 -3.13920092    0.01453085   -1.77527650
 N                 -1.81006127    0.00689579   -1.62911738
 C                 -1.30737323    0.00296486   -0.35277508
 H                  0.78208493   -0.00461510   -0.66080981
 H                 -1.12672112    0.01327677   -2.44218639
 O                 -4.33519806    0.00949850    1.58520415
 N                 -3.66593943    0.02123444   -3.00667746
 H                 -4.66123815    0.03532346   -3.15645600
 H                 -3.06386436    0.02962564   -3.81715124
 H                 -4.92194228    0.01789144   -0.77431371
 O                  3.33896887    0.04101632   -4.56293437
 O                  1.84764071   -0.03459587   -2.33311111
 C                  2.03791534    0.06790533   -4.77139173
 C                  1.19179474    0.01947714   -3.46296367
 O                  1.50209642    0.12650851   -5.84277402
 O                 -0.03259837    0.03624856   -3.53471358
 C                  6.08294839   -0.54227795   -0.68760597
 C                  6.81825129   -0.25384851    0.62262794
 C                  8.30859316   -0.61652787    0.49609394
 C                  8.96574702    0.08761437   -0.69900155
 C                  8.20931212   -0.18868463   -2.00992667
 C                  6.73362993    0.18943800   -1.86519605
 H                  8.82590497   -0.35238471    1.42094337
 H                  6.71792297    0.81202170    0.86583366
 H                  6.35669085   -0.81457344    1.44098964
 H                  6.10878996   -1.61789490   -0.89278408
 H                  8.99617315    1.16846915   -0.51911399
 H                 10.00323467   -0.23694498   -0.80344987
 H                  8.28619603   -1.25383500   -2.26363251
 H                  6.64699970    1.26807935   -1.69628830
 H                  8.40844615   -1.70238401    0.38455301
 N                  5.91101957   -0.09731164   -3.08654462
 H                  6.10336178    0.58000123   -3.82484659
 H                  6.15161494   -1.01251399   -3.47113466
 Pt                 3.89331136   -0.06867630   -2.63774480
 N                  4.62371659   -0.17246254   -0.66051576
 H                  4.11400672   -0.84900394   -0.09498491
 H                  4.50968276    0.73294463   -0.20322595
 H                  8.65712464    0.37040398   -2.83648792
```

Complete citation for the Gaussian code of programs

Gaussian 16. Revision C.01.
Frisch, M. J., Trucks, G. W., Schlegel, H. B., Scuseria, G. E., Robb, M. A., Cheeseman, J. R., Scalmani, G., Barone, V., Petersson, G. A., Nakatsuji, H., Li, X., Caricato, M., Marenich, A. V., Bloino, J., Janesko, B. G., Gomperts, R., Mennucci, B., Hratchian, H. P., Ortiz, J. V., Izmaylov, A. F., Sonnenberg, J. L., Williams, Ding, F., Lipparini, F., Egidi, F., Goings, J., Peng, B., Petrone, A., Henderson, T., Ranasinghe, D., Zakrzewski, V. G., Gao, J., Rega, N., Zheng, G., Liang, W., Hada, M., Ehara, M., Toyota, K., Fukuda, R., Hasegawa, J., Ishida, M., Nakajima, T., Honda, Y., Kitao, O., Nakai, H., Vreven, T., Throssell, K., Montgomery Jr., J. A., Peralta, J. E., Ogliaro, F., Bearpark, M. J., Heyd, J. J., Brothers, E. N., Kudin, K. N., Staroverov, V. N., Keith, T. A., Kobayashi, R., Normand, J., Raghavachari, K., Rendell, A. P., Burant, J. C., Iyengar, S. S., Tomasi, J., Cossi, M., Millam, J. M., Klene, M., Adamo, C., Cammi, R., Ochterski, J. W., Martin, R. L., Morokuma, K., Farkas, O., Foresman, J. B. and Fox, D. J.. Wallingford CT.
